# On the Critical–Subcritical Moments of Moments of Random Characteristic Polynomials: A GMC Perspective

**DOI:** 10.1007/s00220-022-04429-3

**Published:** 2022-06-29

**Authors:** Jonathan P. Keating, Mo Dick Wong

**Affiliations:** 1 Mathematical Institute, University of Oxford, Woodstock Road, Oxford, OX2 6GG UK; 2 Department of Mathematical Sciences, Durham University, Stockton Road, Durham, DH1 3LE UK

## Abstract

We study the ‘critical moments’ of subcritical Gaussian multiplicative chaos (GMCs) in dimensions $d \le 2$. In particular, we establish a fully explicit formula for the leading order asymptotics, which is closely related to large deviation results for GMCs and demonstrates a similar universality feature. We conjecture that our result correctly describes the behaviour of analogous moments of moments of random matrices, or more generally structures which are asymptotically Gaussian and log-correlated in the entire mesoscopic scale. This is verified for an integer case in the setting of circular unitary ensemble, extending and strengthening the results of Claeys et al. and Fahs to higher-order moments.

## Introduction

### Moments of moments of random characteristic polynomials

There has been a good deal of interest in recent years in understanding asymptotically log-correlated Gaussian fields and their extremal processes. In the context of random matrix theory, these structures arise from the study of the logarithms of characteristic polynomials of different ensembles, such as Haar-distributed random matrices from the classical compact groups, the unitary ensemble of random Hermitian matrices and the complex Ginibre ensemble. This has led to a line of investigation into the so-called moments of moments, which capture multifractal properties of the underlying characteristic polynomial as well as non-trivial information about its maxima.

To motivate our discussion, let us consider the circular unitary ensemble (CUE). Suppose $U_N$ is an $N \times N$ Haar-distributed unitary matrix and $P_N(\theta ) = \det (I - U_N e^{-i\theta })$ is the associated characteristic polynomial. For $k, s > 0$, we denote by

$$\begin{aligned} {\mathrm{MoM}}_{\mathrm {U}(N)}(k, s) := \mathbb {E}_{\mathrm {U}(N)} \left[ \left( \frac{1}{2\pi }\int _0^{2\pi } |P_N(\theta )|^{2s}d\theta \right) ^{k}\right] \end{aligned}$$

the (absolute) moments of moments (MoMs) of $P_N(\theta )$. We shall be interested in the behaviour of ${\mathrm{MoM}}_{\mathrm {U}(N)}(k, s)$ as $N \rightarrow \infty$. Due to the multifractality of the random characteristic polynomials, this quantity exhibits a phase transition as one varies the values of (*k*, *s*), and it was conjectured in [25] that

1 $$\begin{aligned} {\mathrm{MoM}}_{\mathrm {U}(N)}(k, s) \overset{N \rightarrow \infty }{\sim } {\left\{ \begin{array}{ll} \left( \frac{G(1+s)^2}{G(1+2s) \Gamma (1-s^2)}\right) ^k \Gamma (1-ks^2) N^{ks^2}, &{} \text {if } k < 1/s^2, \\ c(k, s) N^{k^2s^2 - k+1}, &{} \text {if } k > 1/s^2, \end{array}\right. } \end{aligned}$$

where *G*(*s*) is the Barnes *G*-function, and *c*(*k*, *s*) is some non-explicit constant depending on (*k*, *s*).

Much progress has been made in establishing the asymptotics (1), especially when *k*, *s* are both positive integers, in which case combinatorial approaches are possible. In this setting, the pair (*k*, *s*) always^1^ lies in the supercritical regime $k > 1/s^2$, and the corresponding asymptotics was first verified in [6]. An alternative combinatorial proof was also given in [2], where a connection to the Painlevé V equation was established for *c*(2, *s*), $s \in \mathbb {N}$.

For positive integers *k* and general *s* satisfying $s > -1/4$, most results have been established with the help of the asymptotics of Toeplitz determinants obtained by the Riemann–Hilbert approach.^2^ The case where $k=2$ was treated in [14], where the asymptotic formulae in both regimes in (1) were established alongside the connection of *c*(2, *s*) to the Painlevé V equation. As for general $k \in \mathbb {N}$, the subcritical case $s^2 < 1/k$ was fully established in [23], but only a weaker result ${\mathrm{MoM}}_{U(N)}(k, s) = \Omega (N^{k^2 s^2 - k+1})$ was obtained in the supercritical regime.

Common to all the aforementioned works is the crucial assumption that *k* is a positive integer, so that one can expand the moments and obtain

2 $$\begin{aligned} {\mathrm{MoM}}_{\mathrm {U}(N)}(k, s) = \frac{1}{(2\pi )^k} \int _0^{2\pi } \dots \int _0^{2\pi }\mathbb {E}_{\mathrm {U(N)}} \left[ \prod _{j=1}^k |P_N(\theta _j)|^{2s}\right] d\theta _1 \dots d\theta _k. \end{aligned}$$

From there, the problem becomes the analysis of the asymptotics for the cross moment in the integrand as well as its behaviour as one integrates it over $[0, 2\pi ]^k$. It is not clear, however, how one may analyse the cross moment when $\theta _j$’s are arbitrarily close to each other (except when $k = 2$ [14]), let alone extend the investigation of moments of moments to non-integer values of *k*. Our goal here is to address this situation through the lens of Gaussian multiplicative chaos (GMC).

Before we continue, let us briefly mention the works [1, 7, 8] where analogous moments of moments are studied in the context of classical compact groups, an approximate model for Riemann zeta function, and the branching random walk via the combinatorial approach, as well as the article [13] where the results in [23] are translated to Toeplitz$+$Hankel determinants, yielding similar weak asymptotics for classical compact groups.

### Perspective from the theory of multiplicative chaos

The theory of GMCs studies the construction and properties of a random multifractal measure formally defined as the exponentiation of log-correlated Gaussian fields. First proposed as a model of turbulence in the 60’s, GMCs have attracted a lot of attention in the last two decades due to their central role in the mathematical formulation of Polyakov’s path integral theory of random surfaces, also known as Liouville quantum gravity [19, 22]. The significance of this theory lies in the fact that it describes (at least conjecturally) the scaling limits of a wide class of models in statistical mechanics, and is closely related to other ‘canonical’ geometric objects in the continuum with conformal symmetries, see e.g. [21, 44] and the references therein. Thanks to techniques from conformal field theory and complex analysis, finer results have been obtained in recent years including various exact formulae for Liouville correlation functions and related quantities [31, 36, 41, 42].

In a different direction, GMCs have been used as a tool to study the geometry of the underlying fields, and new applications in other areas of mathematics have also been studied over the last couple of years. For example, it is now known that the characteristic polynomials of many different random matrix ensembles behave asymptotically like GMC measures as the size of the matrix goes to infinity [11, 12, 15, 26, 34, 35, 45]. We would like to exploit such connections to GMCs, and explore the implications of seemingly unrelated exact integrability results from Liouville quantum gravity in the context of random matrix theory.

For illustration, let us revisit our CUE example. It has been shown in [35] that when $s \in (0, 1)$, the sequence of random measures

3 $$\begin{aligned} \frac{|P_N(\theta )|^{2s}}{\mathbb {E}_{\mathrm {U}(N)}|P_N(\theta )|^{2s}} d\theta \end{aligned}$$

converges in distribution (and in the weak$^*$ topology) to the GMC measure $M_{\gamma }(d\theta ) = e^{\gamma X(e^{i\theta }) - \frac{\gamma ^2}{2} \mathbb {E}[X(e^{i\theta })^2]}d\theta$, where $\gamma = \sqrt{2}s$ and *X* is the Gaussian free field

4 $$\begin{aligned} \mathbb {E}\left[ X(x) X(y) \right] = -\log |x-y|, \qquad x, y \in \partial \mathbb {D}= \text {unit circle}, \end{aligned}$$

as the size of the matrix *N* goes to infinity. When $k < 1/s^2 = 2/\gamma ^2$, the *k*-th moment of GMC exists and is given by the Fyodorov–Bouchaud formula [24, 36]

$$\begin{aligned} \mathbb {E}\left[ \left( \frac{1}{2\pi }M_{\gamma }([0, 2\pi ])\right) ^k\right] = \frac{\Gamma (1 - k \frac{\gamma ^2}{2})}{\Gamma (1 - \frac{\gamma ^2}{2})^k}. \end{aligned}$$

It follows from the work of Keating and Snaith [32] that

5 $$\begin{aligned} \mathbb {E}_{\mathrm {U}(N)}|P_N(\theta )|^{2s} \overset{N \rightarrow \infty }{\sim } \frac{G(1+s)^2}{G(1+2s)} N^{s^2}. \end{aligned}$$

Combining both results, we arrive at

6 $$\begin{aligned} \nonumber {\mathrm{MoM}}_{\mathrm {U}(N)}(k, s)&= \mathbb {E}_{\mathrm {U}(N)} \left[ \left( \frac{1}{2\pi }\int _0^{2\pi } |P_N(\theta )|^{2s}d\theta \right) ^{k}\right] \\ \nonumber&= \left( \mathbb {E}_{\mathrm {U}(N)} |P_N(\theta )|^{2s}\right) ^k \mathbb {E}_{\mathrm {U}(N)} \left[ \left( \frac{1}{2\pi } \int _0^{2\pi } \frac{|P_N(\theta )|^{2s}}{\mathbb {E}_{\mathrm {U}(N)}|P_N(\theta )|^{2s}} d\theta \right) ^{k}\right] \\&\overset{N \rightarrow \infty }{\sim } \frac{\Gamma (1 - k s^2)}{\Gamma (1 - s^2)^k} \left[ \frac{G(1+s)^2}{G(1+2s)}\right] ^k N^{ks^2} \end{aligned}$$

which matches the first asymptotics in (1), i.e. the regime $k < 1/s^2$, $0< s < 1$ is essentially resolved.^3^

The focus of this article is to extend the analysis beyond the regime where GMC moments exist, particularly when *k* takes the critical value. In the CUE example this would correspond to the situation where $k\gamma ^2/2 = ks^2 = 1$ with $s^2 < 1$. A naive substitution of $k = 1/s^2$ into (6) results in the blow-up of the factor $\Gamma (1-ks^2)$, justifying the need for a different asymptotic formula in this setting. It is widely conjectured that

7 $$\begin{aligned} {\mathrm{MoM}}_{\mathrm {U}(N)}\left( k, s = \frac{1}{\sqrt{k}}\right) \sim \alpha (k, \frac{1}{\sqrt{k}}) N \log N, \end{aligned}$$

but nothing is known rigorously except for $k=1/s^2 = 2$ where it was shown in [14, Theorem 1.15] that

$$\begin{aligned} \alpha \left( 2, \frac{1}{\sqrt{2}}\right) = \frac{1}{\pi } \frac{G(1+\frac{1}{\sqrt{2}})^2}{G(1+\sqrt{2})}. \end{aligned}$$

In general, the best known result to date is

$$\begin{aligned} \log {\mathrm{MoM}}_{\mathrm {U}(N)}\left( k, \frac{1}{\sqrt{k}}\right) = \log N + \log \log N + \mathcal {O}(1) \end{aligned}$$

which was established for positive integers *k* in [23], and to our knowledge there have been no conjectures giving an explicit formula for the $\mathcal {O}(1)$-term.

While one can no longer directly rely on the convergence of GMC in [35] to derive an asymptotic formula for ${\mathrm{MoM}}_{\mathrm {U}(N)}(k, s)$ in the regime where $ks^2 \ge 1$, one may still consider the following GMC heuristic: if $(X_{\epsilon }(\cdot ))_{\epsilon > 0}$ is a collection of continuous Gaussian fields such that

$$\begin{aligned} M_{\gamma , \epsilon }(dx) := e^{\gamma X_{\epsilon }(x) - \frac{\gamma ^2}{2}\mathbb {E}[X_\epsilon (x)^2]}dx \xrightarrow {\epsilon \rightarrow 0^+} M_{\gamma }(dx) \end{aligned}$$

and the covariance profiles of the log-characteristic polynomials are comparable to those of $X_{\epsilon }$ for suitably chosen $\epsilon = \epsilon (N)$, then perhaps

8 $$\begin{aligned} \mathbb {E}_{\mathrm {U}(N)}\left[ \left( \int _0^{2\pi } \frac{|P_N(\theta )|^{2s}}{\mathbb {E}_{\mathrm {U}(N)}|P_N(\theta )|^{2s}} d\theta \right) ^k\right] \overset{\epsilon \rightarrow 0^+}{\sim } \mathbb {E}\left[ \left( \int _0^{2\pi } M_{\gamma , \epsilon }(dx)\right) ^k\right] . \end{aligned}$$

We shall therefore consider ‘critical moments’ of (regularised) subcritical GMCs (8) in this article, and study their asymptotics as $\epsilon \rightarrow 0^+$.

### Setting and main result

For generality we consider $d \le 2$ so that our result is potentially relevant to not only one-dimensional models like the classical compact groups, but also two-dimensional ones such as complex Ginibre ensemble [48] as well as other asymptotically log-correlated structures in other probability problems.

Let *X* be a log-correlated Gaussian field on some bounded open domain $D \subset \mathbb {R}^d$ with covariance

9 $$\begin{aligned} \mathbb {E}[X(x) X(y)] = -\log |x-y| + f(x, y) \qquad \forall x, y \in D \end{aligned}$$

where *f* is some continuous function on ${\overline{D}} \times {\overline{D}}$.

For $\gamma ^2 < 2d$, the Gaussian multiplicative chaos associated to *X* is defined as the weak$^*$ limit

10 $$\begin{aligned} M_{\gamma }(dx) := \lim _{\epsilon \rightarrow 0^+} M_{\gamma , \epsilon }(x) = \lim _{\epsilon \rightarrow 0^+} e^{\gamma X_\epsilon (x) - \frac{\gamma ^2}{2} \mathbb {E}[X_\epsilon (x)^2]}dx \end{aligned}$$

where $\left( X_{\epsilon }\right) _{\epsilon > 0}$ is some sequence of continuous Gaussian fields on *D* that converges to *X* as $\epsilon \rightarrow 0^+$ in a suitable sense. The covariance structure of such approximate fields are typically of the form

$$\begin{aligned} \mathbb {E}[X_\epsilon (x) X_\epsilon (y)] = -\log \left( |x-y| \vee \epsilon \right) + f_{\epsilon }(x, y) \xrightarrow {\epsilon \rightarrow 0} \mathbb {E}[X(x) X(y)] \qquad \forall x, y \in D \end{aligned}$$

and it is an established fact in the GMC literature that the limiting measure $M_{\gamma }$ is independent of the choice of approximation (see Theorem 5 in Section 2.3 for a version for the mollification construction, or [27] for a generalisation).

It is also well-known that the total mass of $M_\gamma$ possesses moments of all orders strictly less than $p_c := 2d/\gamma ^2$, and for convenience let us call the pair $(p_c, \gamma )$ critical–subcritical. In particular, if $g \ge 0$ is a non-trivial^4^ continuous function on ${\overline{D}}$, then the sequence

11 $$\begin{aligned} \mathbb {E}\left[ \left( \int _D g(x) M_{\gamma , \epsilon }(dx)\right) ^{p_c}\right] \end{aligned}$$

blows up as $\epsilon \rightarrow 0^+$ by Fatou’s lemma. Our first theorem provides a precise description of this behaviour when the underlying approximate fields $X_\epsilon$ are constructed via mollification.

#### Theorem 1

For $d \le 2$ and $\gamma \in (0, \sqrt{2d})$, consider

$$\begin{aligned} M_{\gamma , \epsilon }(dx) = e^{\gamma X_\epsilon (x) - \frac{\gamma ^2}{2}\mathbb {E}[X_\epsilon (x)^2]}dx \end{aligned}$$

with $X_\epsilon := X *\nu _\epsilon$, where *X* is the log-correlated Gaussian field in (9) and $\nu _\epsilon (\cdot ):= \epsilon ^{-d} \nu (\cdot /\epsilon )$ for some mollifier $\nu \in C_c^\infty (\mathbb {R}^d)$.^5^ Suppose $g \ge 0$ is a non-trivial continuous function on ${\overline{D}}$. Writing $p_c = 2d/\gamma ^2$, we have

12 $$\begin{aligned} \begin{aligned}&\mathbb {E}\left[ \left( \int _D g(x) M_{\gamma , \epsilon }(dx)\right) ^{p_c}\right] \\&\qquad \overset{\epsilon \rightarrow 0^+}{\sim } \left( \int _D e^{d(p_c-1) f(u, u)} g(u)^{p_c} du\right) \frac{\gamma ^2}{2}(p_c-1)^2 {\overline{C}}_{\gamma , d}\log \frac{1}{\epsilon }. \end{aligned} \end{aligned}$$

The constant ${\overline{C}}_{\gamma , d}$ is the reflection coefficient of GMC, which is explicitly given by

13 $$\begin{aligned} {\overline{C}}_{\gamma , d} = {\left\{ \begin{array}{ll} \frac{(2\pi )^{\frac{2}{\gamma ^2}-1}}{(1-\frac{\gamma ^2}{2})\Gamma (1-\frac{\gamma ^2}{2})^{\frac{2}{\gamma ^2}}}, &{} d = 1,\\ -\frac{\left( \pi \Gamma (\frac{\gamma ^2}{4}) / \Gamma (1- \frac{\gamma ^2}{4})\right) ^{\frac{4}{\gamma ^2}- 1}}{\frac{4}{\gamma ^2}-1} \frac{\Gamma (\frac{\gamma ^2}{4} - 1)}{\Gamma (1-\frac{\gamma ^2}{4}) \Gamma (\frac{4}{\gamma ^2}-1)}, &{} d = 2. \end{array}\right. } \end{aligned}$$

The expression (12) is reminiscent of the tail expansion of subcritical GMCs, as it was shown in [47] (under extra regularity condition on *f*) that

14 $$\begin{aligned} \mathbb {P}\left( \int _D g(x) M_{\gamma }(dx) > t\right) \overset{t \rightarrow \infty }{\sim } \left( \int _D e^{d(p_c-1) f(u, u)} g(u)^{p_c} du\right) \left( 1 -\frac{1}{p_c}\right) \frac{{\overline{C}}_{\gamma , d}}{t^{p_c}}. \end{aligned}$$

This should not be entirely surprising given that the blow-up of the $p_c$-th moment is intrinsically related to the power law asymptotics of GMCs, and these results together seem to suggest that (14) may also hold for the approximate GMC $M_{\gamma , \epsilon }$ for $t = t(\epsilon )$ tending to infinity not too quickly with respect to $\epsilon \rightarrow 0^+$. We also see the recurring theme of universality for these large-deviation type problems, i.e. the asymptotics only depends on the diagonal of the covariance *f*(*u*, *u*) but not any further specification of the underlying log-correlated field.

The asymptotic formula (12) also looks relatively robust and should hold beyond the mollification setting, e.g. when $X_\epsilon$ is constructed by truncating the series expansion of *X*. We have the following corollary.

#### Corollary 1

Under the same setting as Theorem 1, except that $(X_{\epsilon })_{\epsilon > 0}$ is any collection of continuous Gaussian fields satisfying the following two conditions:

- There exists some $C>0$ such that
  $$\begin{aligned} \limsup _{\epsilon \rightarrow 0^+} \sup _{x, y \in D} \left| \mathbb {E}[X_\epsilon (x) X_\epsilon (y)] + \log (|x-y| \vee \epsilon ) \right| \le C. \end{aligned}$$
- For any $$\delta > 0$$,
  $$\begin{aligned} \lim _{\epsilon \rightarrow 0^+} \sup _{|x-y| \ge \epsilon ^{1-\delta }} \left| \mathbb {E}[X_\epsilon (x) X_\epsilon (y)] - \mathbb {E}[X(x) X(y)]\right|&= 0. \end{aligned}$$

Then the asymptotics in (12) remains true.

*On circular unitary ensemble.* We are able to establish the following asymptotics for Haar-distributed unitary matrices.

#### Theorem 2

Let $k\ge 2$ be any integer. As $N \rightarrow \infty$, we have

15 $$\begin{aligned} {\mathrm{MoM}}_{\mathrm {U}(N)}\left( k, \frac{1}{\sqrt{k}}\right) \overset{N \rightarrow \infty }{\sim } \frac{k-1}{\Gamma (1-\frac{1}{k})^k} \left[ \frac{G(1+\frac{1}{\sqrt{k}})^{2}}{G(1+\frac{2}{\sqrt{k}})}\right] ^k N \log N. \end{aligned}$$

Let us quickly explain how (15) follows from the GMC heuristics. Since the log-characteristic polynomial behaves asymptotically like a log-correlated Gaussian field in the entire mesoscopic regime, we anticipate that the GMC approximation would be accurate, and hence expect

$$\begin{aligned} {\mathrm{MoM}}_{\mathrm {U}(N)}(k, s)&\overset{N \rightarrow \infty }{\sim } \frac{1}{(2\pi )^k} \left( \mathbb {E}_{\mathrm {U}(N)} |P_N(\theta )|^{2s}\right) ^k \mathbb {E}\left[ \left( \int _0^{2\pi } M_{\gamma , \epsilon (N)}(d\theta )\right) ^{k}\right] \end{aligned}$$

to hold for any $k = 1/s^2 > 1$. Based on the discussion in Section 1.2, it is reasonable to believe that the suitable log-correlated field for approximation is given by the Gaussian free field

16 $$\begin{aligned} \mathbb {E}\left[ X(e^{i\theta })X(e^{i\phi })\right] = -\log |e^{i(\theta - \phi )} - 1| \overset{\theta \rightarrow \phi }{=} -\log |\theta - \phi | + \mathcal {O}(\theta - \phi ), \end{aligned}$$

in which case the *f*-function appearing in (9) is simply zero on the diagonal. Meanwhile, the comparison

$$\begin{aligned} \Omega (\epsilon ^{-\frac{k\gamma ^2}{2}}) = \mathbb {E}\left[ e^{\frac{\gamma ^2}{2}\mathbb {E}[X_\epsilon (x)^2]}\right] ^{k} \approx \left( \mathbb {E}|P_N(\theta )|^{2s}\right) ^k = \Omega (N^{ks^2}) \end{aligned}$$

with $\gamma = \sqrt{2}s$ seems to suggest the choice $\epsilon = \epsilon (N) =\Omega (N^{-1})$.The asymptotics (15) then follows by combining all these considerations with Theorem 1. When $k \ge 2$ is an integer, this heuristic argument can be made rigorous, since we may consider the expanded moments (2) and show that

$$\begin{aligned} \mathbb {E}_{\mathrm {U(N)}} \left[ \prod _{j=1}^k |P_N(\theta _j)|^{2s}\right] \qquad \text {and} \qquad \mathbb {E}\left[ \prod _{j=1}^k e^{\gamma X_{\epsilon }(e^{i\theta _j})}\right] \end{aligned}$$

behave similarly as we integrate over $[0, 2\pi )^k$. This will be verified carefully in Section 3.4. This strategy does not work for non-integers *k* unfortunately, but we nevertheless conjecture that

#### Conjecture 1

The asymptotics (15) hold for any $k > 1$.

### Interpretation of results: mesoscopic statistics

Our main result of course does not cover the asymptotics for $\mathbb {E}\left[ \left( \int _D g(x) M_{\gamma , \epsilon }(dx)\right) ^{p}\right]$ in the ‘supercritical’ cases where

- $$\gamma ^2 >2d$$ and $$p = \frac{2d}{\gamma ^2}$$; or
- $$\gamma \in \mathbb {R}$$ but $$p > \frac{2d}{\gamma ^2}$$.

We would like to emphasise that this restriction is not due to any technical reason, but rather is largely due to our motivation for (11) as an accurate toy model that provides the correct leading order.

As we shall see in the proofs, the GMC heuristic (8) is highly plausible in the critical-subcritical setting as long as the original model behaves asymptotically like a log-correlated Gaussian field in the entire mesoscopic scale. Going beyond this phase, the leading order will depend on the microscopic behaviour, and there is no hope to identify the correct leading coefficients even if our GMC toy model still successfully captures the correct order. In the context of CUE, the microscopic statistics is described by the Sine kernel, and it should not be surprising that the present Gaussian analysis does not provide the right answer to the leading order coefficients. The interested readers may find more discussion in Appendix C where we sketch some of the calculations in the supercritical case and explain the connections between the leading order asymptotics and the microscopic regime.

The same GMC heuristic may be applied to other models in probability, and we would like to mention two other examples in random matrix theory.

*Circular beta ensemble* ($C \beta E$). For $\beta > 0$ and $N \in \mathbb {N}$, the C$\beta$E$_N$ is a point process of *N* particles $\theta _1, \dots , \theta _N$ on the unit circle with joint density

$$\begin{aligned} d\mathbb {P}_{\mathrm {C\beta E}(N)}(\theta _1, \dots , \theta _N) \propto \prod _{j < k} |e^{i\theta _j} - e^{i \theta _k}|^\beta \prod _{1 \le k \le N} d\theta _k. \end{aligned}$$

When $\beta = 1, 2, 4$ this exactly corresponds to the eigenvalue distributions of random matrices sampled from the circular orthogonal, unitary and symplectic ensembles respectively, but a general construction is also available for arbitrary $\beta > 0$ in [30]. If we define the ‘characteristic polynomial’ $P_N(\theta ) = \prod _{j=1}^N (1 - e^{i(\theta _j-\theta )})$ as before, then we know from [33] that

$$\begin{aligned} \log |P_N(\theta )| \rightarrow \frac{1}{\sqrt{\beta }} X(e^{i\theta }) \end{aligned}$$

in the sense of finite dimensional distribution as $N\rightarrow \infty$, where $X(\cdot )$ is again the Gaussian free field on the unit circle in (4). Partial progress has been made towards proving the convergence to GMCs in the same article [33]; see also [15] where a different connection between C$\beta$E-characteristic polynomials and GMCs was established.

Not much is known about the asymptotics for moments of moments of C$\beta$E models in general, since the underlying point process is no longer determinantal for $\beta \ne 2$ and powerful techniques such as Riemann-Hilbert analysis no longer apply. The only work in this direction is the recent article [3] where the asymptotics are established for part of the supercritical regime $\beta < 2ks^2$ with integral parameters *k* and *s*, based on symmetric function theory.

We consider the ‘critical-subcritical’ regime based on the GMC heuristics and our main theorem. Let us recall from the work of Keating and Snaith [32] that

$$\begin{aligned} \mathbb {E}_{\mathrm {C\beta E}(N)}\left[ |P_N(\theta )|^{2s}\right]&= \prod _{k=0}^{N-1} \frac{\Gamma (1+\frac{\beta }{2}k) \Gamma (1+2s + \frac{\beta }{2}k)}{\Gamma (1+s+\frac{\beta }{2}k)^2}. \end{aligned}$$

Using the leading order asymptotics derived in [5, Lemma 4.17], we arrive at the following conjecture.

#### Conjecture 2

For any $s> 0$ and $\beta > 0$ satisfying $2s^2 < \beta$ and $k = \frac{\beta }{2s^2}$,

17 $$\begin{aligned} \mathrm {MoM}_{\mathrm {C\beta E}(N)}(k, s)&:= \mathbb {E}_{\mathrm {C\beta E}(N)}\left[ \left( \frac{1}{2\pi } \int _0^{2\pi } |P_N(\theta )|^{2s}d\theta \right) ^{k}\right] \nonumber \\&\overset{N \rightarrow \infty }{\sim } \frac{k-1}{\Gamma (1-\frac{1}{k})^k} \left[ \frac{e^{\Upupsilon ^\beta (1- \frac{\beta }{2}) + \Upupsilon ^\beta (1+2s - \frac{\beta }{2})}}{e^{2\Upupsilon ^\beta (1 + s - \frac{\beta }{2})}}\right] ^k N \log N \end{aligned}$$

where

$$\begin{aligned} \Upupsilon ^\beta (x)&= \frac{\beta }{2} \log G\left( 1+\frac{2x}{\beta }\right) - \left( x - \frac{1}{2}\right) \log \Gamma \left( 1+\frac{2x}{\beta }\right) \\&\qquad + \int _0^\infty \left[ \frac{1}{2t} - \frac{1}{t^2} + \frac{1}{t(e^t-1)}\right] \frac{e^{-xt - 1}}{e^{\frac{\beta }{2}t}-1} dt + \frac{x^2}{\beta } + \frac{x}{2} \end{aligned}$$

and *G* is the Barnes G-function.

#### Remark 1

At first glance it is not obvious how (17) coincides with our earlier conjecture regarding the CUE when $\beta = 2$, but this may be deduced from the equivalent and simpler representation of $\Upupsilon ^\beta (\cdot )$ for rational values of $\beta$, see [5, Lemma 7.1].

*Classical compact groups.* Following the spirit of previous works on unitary matrices, the convergence of characteristic polynomials to GMC measures has recently been established in [26] for other classical compact groups, namely the orthogonal group $\mathrm {O}(N)$ and symplectic group $\mathrm {Sp}(2N)$.^6^ More precisely, if $\mathcal {G}(N) = \mathrm {O}(N)$ or $\mathrm {Sp}(N)$ (the latter case only makes sense when *N* is even), and $P_N(\theta ) = \det (I - U_N e^{-i\theta })$ where $U_N \in \mathcal {G}(N)$ is Haar-distributed, then

$$\begin{aligned} \frac{|P_N(\theta )|^{2s}}{\mathbb {E}_{\mathcal {G}(N)} |P_N(\theta )|^{2s}} ds \xrightarrow [N\rightarrow \infty ]{d} e^{\gamma X(e^{i\theta }) - \frac{\gamma ^2}{2} \mathbb {E}[X(e^{i\theta })^2]}d\theta \end{aligned}$$

where $\gamma = \sqrt{2}s$ as before but now the Gaussian field *X* has covariance

18 $$\begin{aligned} \mathbb {E}[X(e^{i\theta }) X(e^{i\phi })] = -\log \left| \frac{e^{i\theta } - e^{i\phi }}{e^{i\theta } - e^{-i\phi }} \right| , \qquad \forall \theta , \phi \in [0, 2\pi ). \end{aligned}$$

While this log-correlated field has an extra singularity at the complex conjugate and does not exactly satisfy (9), we believe that the GMC heuristic would work equally well here,^7^ provided that the leading order is not dominated by the contribution from the neighbourhood of $\theta = 0$ or $\theta = \pi$ where the behaviour of the field *X* is different.

To apply the GMC heuristic, we again extract the value of the *f*-function on the diagonal: as $\theta \rightarrow \phi$,

$$\begin{aligned} -\log \left| \frac{e^{i\theta } - e^{i\phi }}{e^{i\theta } - e^{-i\phi }} \right| \rightarrow -\log |\theta - \phi | + \log |1-e^{2i\phi }| + \mathcal {O}(\theta - \phi ) \end{aligned}$$

everywhere except for $\phi \in \{0, \pi \}$, which is a set of measure zero and hence may be neglected, i.e. we may just take $f(\theta , \theta ) = \log |1- e^{2i\theta }|$. Unlike the unitary case, we also need to pick a suitable *g*-function for (12), since $\log |P_N(\theta )|$ converges to a non-stationary Gaussian field with non-zero mean (see [26, Theorem 3]). For orthogonal matrices, it is known (e.g. [26, equations (4.32)]) that

$$\begin{aligned} \mathbb {E}_{\mathrm {O}(N)}|P_N(\theta )|^{2s} \overset{N \rightarrow \infty }{=} (1+o(1)) N^{s^2} \frac{G(1+s)^2}{G(1+2s)} |1-e^{2i\theta }|^{-s^2 +s}, \qquad \text {for } \theta \not \in \{0, \pi \} \end{aligned}$$

and so we may want to pick $g(\theta ) = N^{s^2} \frac{G(1+s)^2}{G(1+2s)} |1-e^{2i\theta }|^{-s^2 +s}$. Note that this is a continuous function without any singularity when $s \le 1$. Therefore,

#### Conjecture 3

For any $k>1$ and $s = \frac{1}{\sqrt{k}}$,

$$\begin{aligned}&\mathrm {MoM}_{\mathrm {O}(N)}(k, s) := \mathbb {E}_{\mathrm {O}(N)} \left[ \left( \frac{1}{2\pi }\int _0^{2\pi } |P_N(\theta )|^{2s}\right) ^k\right] \\&\qquad \overset{N \rightarrow \infty }{\sim } \frac{k-1}{\Gamma (1-\frac{1}{k})^k} \left( \frac{1}{2\pi }\int _0^{2\pi } |1-e^{2i\theta }|^{k+\sqrt{k}-2}d\theta \right) \left[ \frac{G(1+\frac{1}{\sqrt{k}})^{2}}{G(1+\frac{2}{\sqrt{k}})}\right] ^k N \log N. \end{aligned}$$

For the symplectic case, we have (see e.g. [26, equations (4.18)])

19 $$\begin{aligned} \mathbb {E}_{\mathrm {Sp}(2N)}|P_{2N}(\theta )|^{2s} \overset{N \rightarrow \infty }{=} (1+o(1)) (2N)^{s^2} \frac{G(1+s)^2}{G(1+2s)} |1-e^{2i\theta }|^{-s^2 -s} \end{aligned}$$

for $\theta \not \in \{0, \pi \}$. Naturally one would like to pick $g(\theta ) = (2N)^{s^2} \frac{G(1+s)^2}{G(1+2s)} |1-e^{2i\theta }|^{-s^2 -s}$, but unlike the orthogonal case the ‘density function’ now has singularities at $\theta = 0$ or $\pi$. This violates our assumption on *g* in Theorem 1, but perhaps the GMC heuristic may be extended to the current case provided that the integral on the RHS of (12) remains finite, i.e. the leading order asymptotics is not dominated by local contributions near $\theta \in \{0, \pi \}$, so that the non-uniformity of (19) or the different behaviour of the covariance kernel (18) near these singularities are irrelevant. Interestingly this gives rise to a ‘singularity threshold’ that is determined by the golden ratio:

#### Conjecture 4

For any $k>(\frac{1+\sqrt{5}}{2})^2$ and $s = \frac{1}{\sqrt{k}}$,

$$\begin{aligned}&\mathrm {MoM}_{\mathrm {Sp}(2N)}(k, s) := \mathbb {E}_{\mathrm {Sp}(2N)} \left[ \left( \frac{1}{2\pi }\int _0^{2\pi } |P_{2N}(\theta )|^{2s}\right) ^k\right] \\&\quad \overset{N \rightarrow \infty }{\sim } \frac{k-1}{\Gamma (1-\frac{1}{k})^k} \left( \frac{1}{2\pi }\int _0^{2\pi } |1-e^{2i\theta }|^{k-\sqrt{k}-2}d\theta \right) \left[ \frac{G(1+\frac{1}{\sqrt{k}})^{2}}{G(1+\frac{2}{\sqrt{k}})}\right] ^k 2N \log N. \end{aligned}$$

#### Remark 2

The leading order of the critical-subcritical moments of moments is conjectured to be $\Omega (N\log N)$ for the three classical compact groups $\mathrm {U}(N)$, $\mathrm {O}(N)$ or $\mathrm {Sp}(N)$. This is in stark contrast to the results in [1] where the exponent of *N* depends on the underlying group, but is not surprising because of different singularity behaviour of the ‘density function’ *g*, which plays a role in determining the size of the leading order in these supercritical cases.

*A note on Gaussian branching random walk.* In [8], the asymptotics for moments of moments were considered for a toy model based on Gaussian branching random walk. For instance, if we take $\gamma = \sqrt{2} \beta \ne 0$, $k= 1/\beta ^2 \in \mathbb {N}$ and $\epsilon = 2^{-n}$, then [8, Theorem 2.1] says that

$$\begin{aligned} \mathbb {E}\left[ \left( \int _0^1 e^{\gamma {\widetilde{X}}_\epsilon (x) - \frac{\gamma ^2}{2}\mathbb {E}[{\widetilde{X}}_\epsilon (x)^2]} dx\right) ^k \right] \sim \frac{\sigma (k)}{\log 2} \log \frac{1}{\epsilon } \qquad \text {as } n \rightarrow \infty \end{aligned}$$

where ${\widetilde{X}}_\epsilon$ is a centred Gaussian field on $D = (0,1)$ with covariance

$$\begin{aligned} \mathbb {E}[{\widetilde{X}}_{2^{-n}}(x) {\widetilde{X}}_{2^{-n}}(y)] = -\log \left( d(x, y) \vee 2^{-n}\right) \end{aligned}$$

and $d(x, y) = 2^{-m}$ if the binary expansions of *x* and *y* only agree up to the first *m* digits.^8^ Neither Theorem 1 nor Corollary 1 would apply to this field^9^ since *d*(*x*, *y*) is not continuous with respect to the Euclidean distance (e.g. consider $x< 1/2 < y$ with *x*, *y* arbitrarily close to 1/2) and $-\log d(x, y)$ is not of the form (9). A one-sided bound may still be obtained, however, if we use Theorem 1 with a different Gaussian field $X_\epsilon = X *\nu _\epsilon$ where

$$\begin{aligned} \mathbb {E}[X(x)X(y)] = -\log |x-y| \qquad \forall x, y \in D. \end{aligned}$$

Indeed, as $|x-y| \le d(x, y)$, the comparison principle from Lemma 2 suggests that

$$\begin{aligned} \frac{\sigma (k)}{\log 2} \le \left( \int _D du\right) \frac{\gamma ^2}{2}(p_c-1)^2 {\overline{C}}_{\gamma , 1} = (2\pi )^{k-1} \frac{k-1}{\Gamma (1-\frac{1}{k})^k} \end{aligned}$$

and this inequality is most likely to be a strict inequality.

### Main idea

Despite the connections to large deviation of GMC, our proof is orthogonal to that in [47]. As we shall see later, the analysis here is closely related to the asymptotics for

20 $$\begin{aligned} \mathbb {E}\left[ \left( \int _0^T e^{\gamma (B_t - \mu t)} dt\right) ^{p_c-1}\right] \qquad \text {as} \qquad T \rightarrow \infty , \end{aligned}$$

where $(B_t)_{t \ge 0}$ is a standard Brownian motion and $\mu = \frac{\gamma }{2}(p_c-1)> 0$. This guiding toy model has been used previously in the literature, such as the renormalisability of Liouville CFT at the Seiberg bound [20] and fusion estimates for GMC [9], but these papers deal with negative moments of exponential functionals of Brownian motion with non-negative drift, the behaviours of which are drastically different from those in the current article.

Going back to (20), we note that $\int _0^\infty e^{\gamma (B_t - \mu t)}dt$ is actually almost surely finite. To understand what causes the blow-up when we compute the $(p_c-1)$-th moment, we make use of Williams’ path decomposition theorem (Theorem 4), which says that $(B_t - \mu t)_{t \ge 0}$

- behaves like a Brownian motion with positive drift $\mu$ (which is denoted by $(B_s^\mu )_{s \ge 0}$) until it hits a height $\mathcal {M}$ that is independently distributed according to $\mathrm {Exp}(2\mu )$;
- runs like $\mathcal {M}- \mathcal {B}_{t - \tau _\mathcal {M}}^\mu$ when one goes beyond the first hitting time $\tau _{\mathcal {M}}$, where $(\mathcal {B}_s^\mu )_{s \ge 0}$ is a Brownian motion with drift $\mu$ conditioned to stay positive.

With these ideas in mind, we can rewrite our exponential functional as

$$\begin{aligned} \mathbb {E}\left[ e^{\gamma (p_c-1)\mathcal {M}} \left( \int _{-\log r}^{T \wedge \tau _\mathcal {M}} e^{\gamma (B_t^\mu -\mathcal {M}) } dt + \int _0^{(T-\tau _\mathcal {M})_+} e^{- \gamma \mathcal {B}_{t }^\mu } dt\right) ^{p_c-1} \right] . \end{aligned}$$

and attribute the blow-up behaviour to the presence of the term $e^{\gamma (p_c-1)\mathcal {M}} = e^{2\mu \mathcal {M}}$ in the expectation.

The use of Williams’ result is inspired by an earlier paper [39] on the tail expansions of 2-dimensional GMC measures, but the analysis there concerns negative moments and is closer in spirit to our “continuity from below” discussion in Lemma 10 or Appendix B. Our main theorem, however, concerns positive moments which require a different treatment, and it is crucial to identify the range of $\mathcal {M}$ that contributes to the leading order of the exponential functional. Not surprisingly, this is given by $\{\mathcal {M}\le \mu T\}$ as supported by the intuition of law of large numbers.

*Organisation of the paper.* The remainder of this article is organised as follows.

- In Section 2, we compile a list of results that will be used in our proofs. This includes a collection of methods for the analysis of Gaussian processes, as well as various facts about path distribution of Brownian motions with drift.
- Our main proofs are presented in Section 3. We first study a slightly unrelated Lemma 10 to illustrate how the path decomposition results may be used in our analysis. We then go back to Theorem 1, showing how the general problem may be reduced to one concerning the exactly log-correlated field from which we obtain our main result. After establishing a technical estimate, we then extend our result to Corollary 1. The section concludes with the proof of Theorem 2 on CUE moments of moments.
- For completeness, we explain in the appendices the current status of the subcritical-subcritical asymptotics (6), and how the leading order coefficient in Theorem 1 may be seen as the renormalised limit of GMC moments from below. We also discuss the reason why GMC heuristics (8) for random matrix models should fail completely as one goes beyond the critical-subcritical regime, and comment briefly on the validity of the strong asymptotics for Toeplitz determinants with slowly merging singularities which is used in Section 3.4.

## Preliminaries

### Gaussian processes

We start with our Gaussian toolbox. The first result we need is a standard change-of-measure lemma.

#### Lemma 1

(Cameron–Martin). Let $N, X(\cdot )$ be centred and jointly Gaussian. Then

21 $$\begin{aligned} \mathbb {E}\left[ e^{N - \frac{1}{2}\mathbb {E}[N^2]} F(X(\cdot ))\right] =\mathbb {E}\left[ F(X(\cdot ) + \mathbb {E}[N X(\cdot )])\right] . \end{aligned}$$

Next, we state Kahane’s interpolation formula.

#### Lemma 2

Let $\rho$ be a Radon measure on *D* and $F: \mathbb {R}_+ \rightarrow \mathbb {R}$ some smooth function with at most polynomial growth at infinity, and suppose $G_0, G_1$ are two centred continuous Gaussian fields on *D*. For $t \in [0,1]$, define $G_t(x) := \sqrt{t} G_1(x) + \sqrt{1-t} G_0(x)$ and

$$\begin{aligned} \varphi (t) := \mathbb {E}\left[ F(W_t)\right] \qquad \text {where}\qquad W_t := \int _D e^{G_t(x) - \frac{1}{2} \mathbb {E}[G_t(x)^2]} \rho (dx). \end{aligned}$$

Then the derivative of $\varphi$ is given by

22 $$\begin{aligned} \varphi '(t)&= \frac{1}{2} \int _D \int _D\left( \mathbb {E}[G_0(x) G_0(y)] - \mathbb {E}[G_1(x) G_1(y)]\right) \nonumber \\&\quad \times \mathbb {E}\left[ e^{G_t(x) + G_t(y) - \frac{1}{2} \mathbb {E}[G_t(x)^2] - \frac{1}{2} \mathbb {E}[ G_t(y)^2] }F''(W_t)\right] \rho (dx) \rho (dy). \end{aligned}$$

In particular, if

$$\begin{aligned} \mathbb {E}[G_0(x) G_0(y)] \le \mathbb {E}[G_1(x) G_1(y)] \qquad \forall x, y \in D, \end{aligned}$$

then for any (integrable) convex function *F* and (non-negative) measure $\mu$ on *D*, we have

23 $$\begin{aligned} \mathbb {E}\left[ F\left( \int _D e^{G_0(x) - \frac{1}{2}\mathbb {E}[G_0(x)^2]} \rho (dx) \right) \right] \le \mathbb {E}\left[ F\left( \int _D e^{G_1(x) - \frac{1}{2}\mathbb {E}[G_1(x)^2]} \rho (dx)\right) \right] . \end{aligned}$$

A simple corollary to Lemma 2 is as follows:

#### Corollary 2

Suppose $G_0, G_1$ are two continuous centred Gaussian fields on *D* such that

$$\begin{aligned} \mathbb {E}[G_0(x) G_0(y)] - \mathbb {E}[G_1(x) G_1(y)] \le C \qquad \forall x, y \in D \end{aligned}$$

for some $C > 0$, then for any (non-negative) measure $\mu$, we have

$$\begin{aligned}&\mathbb {E}\left[ \left( \int _D e^{\gamma G_0(x) - \frac{\gamma ^2}{2}\mathbb {E}[G_0(x)^2]} \mu (dx) \right) ^k\right] \\&\qquad \ge e^{\frac{\gamma ^2}{2}k(k-1)C} \mathbb {E}\left[ \left( \int _D e^{\gamma G_1(x) - \frac{\gamma ^2}{2}\mathbb {E}[G_1(x)^2]} \mu (dx) \right) ^k\right] \end{aligned}$$

for $k \in [0, 1]$, and the inequality is reversed for $k \not \in [0,1]$. In particular, if the two-sided inequality

$$\begin{aligned} |\mathbb {E}[G_0(x) G_0(y)] - \mathbb {E}[G_1(x) G_1(y)]| \le C \qquad \forall x, y \in D \end{aligned}$$

is satisfied instead, then for any $k \in \mathbb {R}$ we have

$$\begin{aligned} \frac{\mathbb {E}\left[ \left( \int _D e^{\gamma G_0(x) - \frac{\gamma ^2}{2}\mathbb {E}[G_0(x)^2]} \mu (dx) \right) ^k\right] }{\mathbb {E}\left[ \left( \int _D e^{\gamma G_1(x) - \frac{\gamma ^2}{2}\mathbb {E}[G_1(x)^2]} \mu (dx) \right) ^k\right] } \in [ e^{-\frac{\gamma ^2}{2}|k(k-1)| C}, e^{\frac{\gamma ^2}{2}|k(k-1)| C}]. \end{aligned}$$

#### Proof

Suppose $k \in [0,1]$ such that $x \mapsto x^k$ is concave. Let $\mathcal {N}_C$ be an independent Gaussian random variable with variance *C*, then

$$\begin{aligned} \mathbb {E}\left[ (G_1(x) + \mathcal {N}_C)(G_1(y) + \mathcal {N}_C)\right] = \mathbb {E}\left[ G_1(x) G_1(y)\right] + C \ge \mathbb {E}[G_0(x) G_0(y)]. \end{aligned}$$

By Kahane’s inequality, we have

$$\begin{aligned}&\mathbb {E}\left[ \left( \int _D e^{\gamma G_0(x) - \frac{\gamma ^2}{2} \mathbb {E}[G_0(x)^2]}\mu (dx)\right) ^k\right] \\&\qquad \ge \mathbb {E}\left[ \left( \int _D e^{\gamma (G_1(x) + \mathcal {N}_C) - \frac{\gamma ^2}{2} \mathbb {E}[(G_1(x)+\mathcal {N}_C)^2]}\mu (dx)\right) ^k\right] \\&\qquad = e^{\frac{\gamma ^2}{2}k(k-1)C} \mathbb {E}\left[ \left( \int _D e^{\gamma G_1(x) - \frac{\gamma ^2}{2} \mathbb {E}[G_1(x)^2]}\mu (dx)\right) ^k\right] . \end{aligned}$$

The case where $k \not \in [0,1]$ is similar. $\square$

### On Brownian motions and 3-dimensional Bessel processes

This subsection collects a few important results regarding the path distributions of Brownian motions as well as 3-dimensional Bessel processes starting from 0 (or $\mathrm {BES}_0(3)$-processes in short). We shall be using these notations throughout the entire paper.

- $(B_t)_{t \ge 0}$ is a standard Brownian motion.
- $$(\beta _t)_{t \ge 0}$$ is a $$\mathrm {BES}_0(3)$$-process.
- For $$\mu \ge 0$$:
  - $(B_t^\mu )_{t \ge 0}$ is a Brownian motion with drift $\mu$.
  - $(\mathcal {B}_t^\mu )_{t \ge 0}$ is a Brownian motion with drift $\mu$ conditioned to stay non-negative, and $(\mathcal {B}_t^\mu )_{t \in \mathbb {R}}$ a two-sided version of it, i.e. $(\mathcal {B}_t^\mu )_{t \ge 0}$ and $(\mathcal {B}_{-t}^\mu )_{t \ge 0}$ are two independent copies.

To be more precise:

#### Definition 1

$(\mathcal {B}_t^\mu )_{t \ge 0}$ is a Markov process starting from the origin with infinitesimal generator

$$\begin{aligned} \frac{1}{2}\frac{d^2}{dx^2} + \mu \coth (\mu x) dx, \end{aligned}$$

or equivalently solution to the singular stochastic differential equation

24 $$\begin{aligned} d\mathcal {B}_t^\mu = \mu \coth (\mu \mathcal {B}_t^\mu ) dt + dW_t, \qquad \mathcal {B}_0^\mu = 0 \end{aligned}$$

where $(W_t)_{t \ge 0}$ is the Wiener process. These definitions extend to the case $\mu = 0$, where $(\mathcal {B}_t^0)_{t \ge 0}$ should be interpreted as a $\mathrm {BES}_0(3)$-process $(\beta _t)_{t \ge 0}$ which solves

$$\begin{aligned} d\beta _t = \frac{1}{\beta _t} dt + dW_t, \qquad \beta _0 = 0. \end{aligned}$$

(In particular, despite its colloquial name, the process $(\mathcal {B}_t^\mu )_{t \ge 0}$ and similarly its two-sided analogue have discontinuous drift at $t = 0$.)

Note that $(\mathcal {B}_t^\mu )_{t \ge 0}$ is known under different names in the literature, such as *the radial part of a* 3 *-dimensional Brownian motion with drift* $\mu \ge 0$. We prefer to call it a conditioned drifting Brownian motion because this is the more commonly known name in the GMC–related literature. Indeed $(\mathcal {B}_t^\mu )_{t \ge 0}$ can be constructed via such conditioning procedure in the sense of Doob’s theory of *h*-transform, see e.g. [46, Section 2.4] and [37, Section 3] and the references therein.

Let us begin with an elementary lemma on stochastic dominance.

#### Lemma 3

$(\mathcal {B}_t^{\mu _1})_{t \ge 0}$ is stochastically dominated by $(\mathcal {B}_t^{\mu _2})_{t \ge 0}$ whenever $\mu _1 \le \mu _2$. This comparison may be extended to $\mu _1 = 0$, in which case we interpret $(\mathcal {B}_t^{0})_{t \ge 0}$ as a $\mathrm{BES}_0(3)$-process $(\beta _t)_{t \ge 0}$.

#### Proof

The stochastic order should immediately follow from a suitable conditioning construction, but we nevertheless check it by showing that the drift in (24) is monotonically increasing in $\mu$: if we write

$$\begin{aligned} m_x(\mu ) := \mu \coth (\mu x) = \mu \frac{e^{\mu x} + e^{- \mu x}}{e^{\mu x} - e^{-\mu x}} \end{aligned}$$

for any fixed $x > 0$, then

$$\begin{aligned} \frac{\partial }{\partial \mu } m_x(\mu )&= \frac{e^{\mu x} + e^{- \mu x}}{e^{\mu x} - e^{-\mu x}} + \mu x \frac{(e^{\mu x} - e^{- \mu x})^2 - (e^{\mu x} + e^{- \mu x})^2}{(e^{\mu x} - e^{-\mu x})^2}\\&= \frac{e^{2\mu x} - e^{-2\mu x} - 4\mu x}{(e^{\mu x} - e^{- \mu x})^2} \ge 0 \end{aligned}$$

since $$\partial _\mu (e^{2\mu x} - e^{-2\mu x} - 4\mu x) \ge 0$$ for $$\mu x \ge 0$$. $$\square $$

We now mention the generalised “2M-B" theorem due to Rogers and Pitman, which provides a duality between $(B_t^\mu )_{t \ge 0}$ and $(\mathcal {B}_t^\mu )_{t \ge 0}$.

#### Theorem 3

(cf. [37, Theorem 1]). Let $\mu \ge 0$, and write

$$\begin{aligned} S_t^\mu := \max _{s \le t} B_s^\mu , \qquad J_t^\mu := \inf _{s \ge t} \mathcal {B}_s^\mu . \end{aligned}$$

Then we have

25 $$\begin{aligned} (2S_t^\mu - B_t^\mu , S_t^\mu )_{t \ge 0} \overset{d}{=} (\mathcal {B}_t^\mu , J_t^\mu )_{t \ge 0} \end{aligned}$$

where the $\mu = 0$ case also holds if we interpret $(\mathcal {B}_t^{\mu =0})_{t \ge 0}$ as a $\mathrm {BES}_0(3)$-process $(\beta _t)_{t \ge 0}$ accordingly.

A simple but useful corollary of the above result is the following.

#### Corollary 3

Let $\mu \ge 0$ and $x > 0$. If we define

$$\begin{aligned} T_x := \sup \{t > 0: \mathcal {B}_t^\mu = x\}, \end{aligned}$$

then the process $(\mathcal {B}_{T_x + t}^\mu - \mathcal {B}_{T_x}^\mu )_{t \ge 0}$ is independent of $(\mathcal {B}_t^\mu )_{t \le T_x}$ and again distributed as $(\mathcal {B}_t^\mu )_{t \ge 0}$.

#### Proof

Given $(\mathcal {B}_t^\mu )_{t \ge 0}$, we define $J_t^\mu = \inf _{s \ge t} \mathcal {B}_s^\mu$ and set

$$\begin{aligned} B_t^\mu := 2J_t^\mu - \mathcal {B}_t^\mu , \qquad S_t^\mu = \max _{s \le t} B_s^\mu . \end{aligned}$$

Then according to Theorem 3, $(B_t^\mu )_{t \ge 0}$ is a Brownian motion with drift $\mu$, and $J_t^\mu = S_{t}^\mu$ for any $t>0$. We then observe that

$$\begin{aligned} T_x = \inf \{t> 0: J_t^\mu = x\} = \inf \{t > 0: S_t^\mu = x\} \end{aligned}$$

is a stopping time for $(B_t^\mu )_{t \ge 0}$. By the strong Markov property, the process

$$\begin{aligned} ({\widetilde{B}}_t^\mu )_{t \ge 0} = (B_{T_x + t}^\mu - B_{T_x}^\mu )_{t \ge 0} \end{aligned}$$

is independent of $(B_t^\mu )_{t \le T_x}$, or equivalently $(\mathcal {B}_t^\mu )_{t \le T_x}$, and it is clear that $({\widetilde{B}}_t^\mu )_{t \ge 0} \overset{d}{=} (B_t^\mu )_{t \ge 0}$. Combining this with the fact that $\mathcal {B}_{T_x}^\mu = x = J_{T_x}^\mu = B_{T_x}^\mu$, we obtain

$$\begin{aligned} \mathcal {B}_{T_x+t}^\mu - \mathcal {B}_{T_x}^\mu&= (2J_{T_x + t}^\mu - B_{T_x + t}^\mu ) - (2J_{T_x}^\mu - B_{T_x}^\mu )\\&= 2(S_{T_x + t}^\mu - S_{T_x}^\mu ) - (B_{T_x + t}^\mu -B_{T_x}^\mu ) = 2{\widetilde{S}}_{t}^\mu - {\widetilde{B}}_t^\mu \end{aligned}$$

where ${\widetilde{S}}_t^\mu := S_{T_x + t}^\mu - S_{T_x}^\mu = \max _{s \le t} {\widetilde{B}}_{s}^\mu$. The result follows from one final application of Theorem 3. $\square$

Note that Theorem 3 is intrinsically related to the work of Williams [46] (see Remarks in [37, Section 5] for a discussion of their connections), and we would like to highlight two important consequences that will play an indispensable role in our main proof. The first one concerns the time reversal of Brownian motion from its first hitting time.

#### Lemma 4

(cf. [37, Corollary 1]). Let $\mu > 0$ and suppose $\tau _x= \inf \{t > 0: B_t^\mu = x\}$ is the first hitting time of $x>0$ by $(B_t^\mu )_{t \ge 0}$. Then

26 $$\begin{aligned} (B_{\tau _x - t}^\mu )_{t \in [0, \tau _x]} \overset{d}{=} (x - \mathcal {B}_t^\mu )_{t \in [0, L_{x}]} \end{aligned}$$

where $L_{x} = \sup \{t > 0: \mathcal {B}_t^{\mu } = x\}$ is the last hitting time of $(\mathcal {B}_t^\mu )_{t \ge 0}$.

The second one is the celebrated result of Williams on the path decomposition of Brownian motions with drift.^10^

#### Theorem 4

(cf. [46, Theorem 2.2], [37, Corollary 2]). Let $\mu > 0$ and consider the following independent objects:

- $(B_t^\mu )_{t \ge 0}$ is a Brownian motion with drift $\mu$.
- $\mathcal {M}$ is an $\mathrm {Exp}(2\mu )$ variable.
- $(\mathcal {B}_t^\mu )_{t \ge 0}$ is a Brownian motion with drift $\mu$ conditioned to stay non-negative.

Then the process $(R_t)_{t \ge 0}$ defined by

27 $$\begin{aligned} R_t = {\left\{ \begin{array}{ll} B_t^\mu &{} t \le \tau _\mathcal {M}\\ \mathcal {M}- \mathcal {B}_{t - \tau _\mathcal {M}}^{\mu } &{} t \ge \tau _\mathcal {M}, \end{array}\right. } \end{aligned}$$

where $\tau _x = \inf \{t > 0: B_t^\mu = x\}$ as usual, is a Brownian motion with drift $-\mu$.

### Gaussian multiplicative chaos

In this subsection we collect a few facts and fix some notations about multiplicative chaos. Before we begin, let us recall that $X(\cdot )$ is said to be a log-correlated Gaussian field on a bounded open domain $D \subset \mathbb {R}^d$ if it is a (centred) Gaussian field on *D* with covariance of the form

$$\begin{aligned} \mathbb {E}[X(x) X(y)] = - \log |x-y| + f(x, y) \qquad \forall x, y \in D \end{aligned}$$

where (for the purpose of this paper) the function *f* is continuous on ${\overline{D}} \times {\overline{D}}$. The logarithmic singularity on the diagonal of the covariance kernel means that $X(\cdot )$ is not defined pointwise and has to be interpreted as a generalised function: for any two test functions $\phi _1, \phi _2 \in C_c^\infty (D)$, the pair of random variables (formally written as)

$$\begin{aligned} \left( \int _D X(x) \phi _1(x)dx, \int _D X(x) \phi _2(x)dx\right) \end{aligned}$$

is jointly Gaussian with mean 0 and covariance given by

$$\begin{aligned} \int _{D \times D} \phi _1(x) \underbrace{\Bigg [ - \log |x-y| + f(x, y)\Bigg ]}_{=\mathbb {E}[X(x)X(y)]} \phi _2(y) dxdy, \end{aligned}$$

and the set of test “functions" may be extended to the set of finite (signed) measures

$$\begin{aligned} \left\{ \phi (d\cdot ): \mathrm {supp}(\phi ) \subset D \text { and }~ \int _{D \times D} \mathbb {E}[X(x)X(y)] \phi (dx) \phi (dy) < \infty \right\} . \end{aligned}$$

More concretely, the log-correlated field $X(\cdot )$ may be constructed via Karhunen–Loève expansion and viewed as an abstract random variable in the negative Sobolev space $H^{-\epsilon }(\mathbb {R}^d)$ for any $\epsilon > 0$; we refer the interested readers to [28, Section 2.1] for a self-contained proof of the Sobolev regularity.

Given a log-correlated field $X(\cdot )$ on *D* and $\gamma \in (0, \sqrt{2d})$, the associated Gaussian multiplicative chaos (GMC) is formally defined as the random measure

$$\begin{aligned} M_\gamma (dx) = e^{\gamma X(x) - \frac{\gamma ^2}{2}\mathbb {E}[X(x)^2]}dx. \end{aligned}$$

While the exponentiation of $X(\cdot )$ is not well-defined, the GMC measure may be constructed via various methods, such as martingale limits [29], smoothing procedures [4, 38], and a generic approach based on the abstract language of Cameron–Martin space is also available [43]. The main takeaway is that all these constructions lead to the same random measure and we are free to choose the most convenient one to work with. The following formulation is based on [4].

#### Theorem 5

Let $X(\cdot )$ be a log-correlated Gaussian field on $D \subset \mathbb {R}^d$ and $X_\epsilon := X *\nu _\epsilon$ where $\nu \in C_c^\infty (D)$ is a mollifier. For any fixed $\gamma \in (0, \sqrt{2d})$, the sequence of random measures

$$\begin{aligned} M_{\gamma , \epsilon }(dx) := e^{\gamma X_\epsilon (x) - \frac{\gamma ^2}{2}\mathbb {E}[X_\epsilon (x)^2]}dx, \qquad x \in D \end{aligned}$$

converges in probability, as $\epsilon \rightarrow 0^+$, to a non-trivial and non-atomic random measure $M_{\gamma }(dx)$ in the space of Radon measures on *D* equipped with the weak$^*$ topology. Moreover, the limit $M_{\gamma }(dx)$ is independent of the choice of $\nu \in C_c^\infty (D)$.

We will need to deal with GMC measures associated to *exact fields* and related objects; these are summarised in the definition below.

#### Definition 2

Let $d \le 2$. By exact field, we mean the log-correlated Gaussian field ${\overline{X}}$ defined on the (closed) unit ball with covariance

28 $$\begin{aligned} \mathbb {E}\left[ {\overline{X}}(x) {\overline{X}}(y)\right] = -\log |x-y| \qquad \forall x, y \in {\overline{B}}(0,1). \end{aligned}$$

The field ${\overline{X}}$ can be decomposed into two independent Gaussian components

29 $$\begin{aligned} \mathbb {E}\left[ {\overline{X}}(x) {\overline{X}}(y)\right]&= -\log |x| \vee |y| + \log \frac{|x| \vee |y|}{|x-y|}\nonumber \\&=: \mathbb {E}[B_{-\log |x|} B_{-\log |y|}] + \mathbb {E}\left[ {\widehat{X}}(x) {\widehat{X}}(y)\right] \end{aligned}$$

where $(B_t)_{t \ge 0}$ is a standard Brownian motion. We also denote by^11^

30 $$\begin{aligned} Z_\gamma (dt) :={\left\{ \begin{array}{ll} e^{\gamma {\widehat{X}}(e^{-t}) - \frac{\gamma ^2}{2} \mathbb {E}[{\widehat{X}}(e^{-t})^2]} dt + e^{\gamma {\widehat{X}}(-e^{-t}) - \frac{\gamma ^2}{2} \mathbb {E}[{\widehat{X}}(-e^{-t})^2]} dt &{} d = 1 \\ \left( \int _0^{2\pi } e^{\gamma {\widehat{X}}(e^{-t} e^{i\theta }) - \frac{\gamma ^2}{2} \mathbb {E}[{\widehat{X}}(e^{-t} e^{i\theta })^2]}d\theta \right) dt &{} d = 2 \end{array}\right. } \end{aligned}$$

the GMC measure associated to ${\widehat{X}}$.

#### Remark 3

We elaborate on a few things in Definition 2 for those who may not be familiar with the theory of log-correlated fields and multiplicative chaos.

- The fact that the covariance kernel (29) of ${\overline{X}}$ can be split into two independent parts follows from the radial-lateral decomposition of Gaussian free field in $d=2$ (the case $d=1$ may be seen as a one-dimensional restriction). Indeed one can construct the Brownian motion $B_{-\log |x|}$ by considering the circle averaging
  31 $$\begin{aligned} B_{-\log |x|} := \frac{1}{2\pi } \int _0^{2\pi } X(|x| e^{i\theta }) d\theta . \end{aligned}$$
  One can then define ${\widehat{X}}(x) := X(x) - B_{-\log |x|}$ and verify (using rotational symmetry) that
  $$\begin{aligned} \mathbb {E}\left[ {\widehat{X}}(x) B_{-\log |y|}\right] = 0 \qquad \forall x, y \in {\overline{B}}(0, 1) \end{aligned}$$
  which implies independence due to Gaussianity.
- A priori, the field ${\widehat{X}}$ (often known as the lateral part of ${\overline{X}}$) is defined on ${\overline{B}}(0,1)$ in the sense of a generalised Gaussian field, but its scale invariance (see Lemma 5 below) implies that it may be extended to the entire $\mathbb {R}^2$ (on a suitable probability space).^12^ Since $B_{-\log |\cdot |}$ has a continuous version (as a Brownian motion), ${\widehat{X}}$ inherits the regularity of ${\overline{X}}$ and may be seen as an abstract random variable living in a (local) Sobolev space of negative exponent.
- $Z_{\gamma }(\cdot )$ (and its formal expression) should be interpreted in the sense of Theorem 5, i.e. the weak$^*$ limit (in probability) of
  $$\begin{aligned} Z_{\gamma , \epsilon }(dt) := {\left\{ \begin{array}{ll} e^{\gamma {\widehat{X}}_\epsilon (e^{-t}) - \frac{\gamma ^2}{2} \mathbb {E}[{\widehat{X}}_\epsilon (e^{-t})^2]} dt + e^{\gamma {\widehat{X}}_\epsilon (-e^{-t}) - \frac{\gamma ^2}{2} \mathbb {E}[{\widehat{X}}_\epsilon (-e^{-t})^2]} dt &{} d = 1 \\ \left( \int _0^{2\pi } e^{\gamma {\widehat{X}}_\epsilon (e^{-t} e^{i\theta }) - \frac{\gamma ^2}{2} \mathbb {E}[{\widehat{X}}_\epsilon (e^{-t} e^{i\theta })^2]}d\theta \right) dt &{} d = 2 \end{array}\right. } \end{aligned}$$
  where ${\widehat{X}}_{\epsilon } (\cdot ):= ({\widehat{X}} *\nu _\epsilon )(\cdot )$, and again the limit is independent of the choice of mollifiers $\nu \in C_c^\infty (\mathbb {R}^d)$. Similar to ${\widehat{X}}$, the domain of $Z_{\gamma }(d\cdot )$ can be extended to the entire real line $\mathbb {R}$.

For future reference, we record the invariance property of ${\widehat{X}}$ and $Z_{\gamma }$ in the following lemma.

#### Lemma 5

For any $c > 0$, we have

$$\begin{aligned} \left( {\widehat{X}}(cx)\right) _{x \in \mathbb {R}^2} \overset{d}{=} \left( {\widehat{X}}(x)\right) _{x \in \mathbb {R}^2} \qquad \text {and} \qquad \left( Z_{\gamma } (dx)\right) _{x \in \mathbb {R}} \overset{d}{=}\left( Z_{\gamma }\circ \phi _c (dx)\right) _{x \in \mathbb {R}} \end{aligned}$$

where $\phi _c: x \mapsto x+c$ is the shift operator.

#### Proof

The scale invariance of ${\widehat{X}}$ follows from a simple covariance check that^13^

$$\begin{aligned} \mathbb {E}\left[ {\widehat{X}}(cx) {\widehat{X}}(cy)\right] = \log \frac{|cx| \vee |cy|}{|cx-cy|} =\log \frac{|x| \vee |y|}{|x-y|}= \mathbb {E}\left[ {\widehat{X}}(x) {\widehat{X}}(y)\right] . \end{aligned}$$

As for the shift invariance of $Z_{\gamma }$, let us start by considering $Z_{\gamma , \epsilon }$ associated to ${\widehat{X}}_{\epsilon } := {\widehat{X}} *\nu _\epsilon$ for some mollifier $\nu \in C_c^\infty (\mathbb {R}^d)$. Since

32 $$\begin{aligned} \left( {\widehat{X}}_{\epsilon }(e^{-c}x) \right) _{x \in \mathbb {R}^d}&= \left( \int {\widehat{X}}(e^{-c}x - \epsilon u) \nu (u) du\right) _{x \in \mathbb {R}^d}\nonumber \\&\overset{d}{=} \left( \int {\widehat{X}}(x - e^c \epsilon u) \nu (u) du\right) _{x \in \mathbb {R}^d} = \left( {\widehat{X}}_{e^c\epsilon }(x) \right) _{x \in \mathbb {R}^d} \end{aligned}$$

by the scale invariance of ${\widehat{X}}$, we see that

33 $$\begin{aligned} \left( Z_{\gamma , \epsilon }\circ \phi _c(dt)\right) _{t \in \mathbb {R}} \overset{d}{=} \left( Z_{\gamma , e^c\epsilon }(dt)\right) _{t \in \mathbb {R}} \end{aligned}$$

and sending $\epsilon \rightarrow 0^+$ on both sides this implies $Z_{\gamma } \circ \phi _c \overset{d}{=} Z_{\gamma }$. $\square$

Let us also recall from [47, Appendix A] the various probabilistic representations of the reflection coefficient ${\overline{C}}_{\gamma , d}$.

#### Lemma 6

Let $d \le 2, \gamma \in (0, \sqrt{2d})$ and $p_c = 2d/\gamma ^2$. The reflection coefficient ${\overline{C}}_{\gamma , d}$ appearing in Theorem 1 is equal to

34 $$\begin{aligned} {\overline{C}}_{\gamma , d} = \lim _{t \rightarrow \infty } t^{p_c - 1} \mathbb {P}\left( \int _{|x| \le r} |x|^{-\gamma ^2} {\overline{M}}_{\gamma }(dx) > t \right) \end{aligned}$$

where ${\overline{M}}_\gamma (dx) = e^{\gamma {\overline{X}}(x) - \frac{\gamma ^2}{2}\mathbb {E}[{\overline{X}}(x)^2]}dx$ is the Gaussian multiplicative chaos associated to the exact field ${\overline{X}}(\cdot )$, and this limit is independent of the value of $r \in (0, 1]$. In particular, it has the alternative probabilistic representation

35 $$\begin{aligned} {\overline{C}}_{\gamma , d} = \mathbb {E}\left[ \left( \int _{-\infty }^\infty e^{-\gamma \mathcal {B}_t^\mu } Z_\gamma (dt)\right) ^{p_c-1}\right] \end{aligned}$$

with $\mu = \frac{\gamma }{2}(p_c-1) > 0$ where $(\mathcal {B}_t^\mu )_{t \ge 0}$ is independent of $Z_{\gamma }(\cdot )$.

#### Remark 4

The fact that the limit (34) is independent of the value $r > 0$ is a consequence of the fact that $\mathbb {E}\left[ {\overline{M}}_\gamma (B(0, 1))^{p_c-1}\right] < \infty$; see [47, Theorem 1] for finer results regarding the tail asymptotics of subcritical GMCs.

As for the fact that the probabilistic representations in Lemma 6 coincide with the expressions in (13), this requires a long detour to the exact integrability of Liouville conformal field theory which is beyond the scope of this paper. The interested readers may consult [39, Section 1.1 and 4.2] and [47, Appendix A] for a brief discussion, and [31, 36] for the derivations of these remarkable formulae.

### Some estimates

In this subsection we collect some basic estimates. The first one concerns the construction of GMC measures via mollifications.

#### Lemma 7

Using the notations in Theorem  5, the following are true:

- (cf. [4, Lemma 3.5]) The covariance of $X_\epsilon$ is of the form
  $$\begin{aligned} \mathbb {E}[X_{\epsilon }(x)X_{\epsilon }(y)] = -\log \left( |x-y| \vee \epsilon \right) + f_\epsilon (x, y) \end{aligned}$$
  where $$\sup _{\epsilon > 0} \sup _{x, y \in D} |f_\epsilon (x, y)| < \infty $$.
- (cf. [38, Proposition 3.5] or [10, Section 3.7]) Let $S \subset D$ be a bounded open subset and $\alpha \in (0, \frac{2d}{\gamma ^2})$. Then the collection of random variables $(M_{\gamma , \epsilon }(S)^\alpha )_{\epsilon > 0}$ is uniformly integrable. In particular,
  $$\begin{aligned} \mathbb {E}\left[ M_{\gamma , \epsilon }(S)^\alpha \right] \xrightarrow {\epsilon \rightarrow 0^+} \mathbb {E}\left[ M_{\gamma }(S)^\alpha \right] < \infty . \end{aligned}$$

We now state the multifractality of (regularised) GMC measures.

#### Lemma 8

Let $\gamma \in (0, \sqrt{2d})$, and consider a collection of continuous Gaussian fields^14^$\{X_\epsilon (\cdot )\}_{\epsilon > 0}$ on *D* with covariance of the form

$$\begin{aligned} \mathbb {E}\left[ X_\epsilon (x) X_\epsilon (y)\right] = -\log \left( |x-y| \vee \epsilon \right) + f_\epsilon (x, y) \qquad \forall x, y \in D \end{aligned}$$

where $C_f:= \sup _{\epsilon > 0} \sup _{x, y \in D} |f_\epsilon (x, y)| < \infty$. Suppose *D* contains the ball *B*(0, *r*) centred at origin with radius $r \in [\epsilon , 1/2]$. Then for any $\alpha \in [0, \frac{2d}{\gamma ^2})$ and $\kappa \ge 0$, there exists some $C>0$ possibly depending on $C_f, \gamma , \alpha , \kappa$ but independent of $\epsilon , r$ such that the random measure

$$\begin{aligned} M_{\gamma , \epsilon }(dx) := e^{\gamma X_\epsilon (x) - \frac{\gamma ^2}{2} \mathbb {E}[X_\epsilon (x)^2]}dx \end{aligned}$$

satisfies the moment estimates

36 $$\begin{aligned} \mathbb {E}\left[ M_{\gamma , \epsilon }(B(0, r))^\alpha \right]&\le C r^{\alpha d+ \frac{\gamma ^2}{2}\alpha (1-\alpha )}, \end{aligned}$$

37 $$\begin{aligned} \mathbb {E}\left[ \left( \int _{\epsilon \le |x| \le r} |x|^{-\kappa \gamma }M_{\gamma , \epsilon }(dx)\right) ^\alpha \right]&\le C\left( 1 \vee \epsilon ^{\alpha (d-\kappa \gamma ) + \frac{\gamma ^2}{2}\alpha (1-\alpha )}\right) . \end{aligned}$$

#### Proof

Both of these estimates are standard in the literature but for completeness we give a sketch of the claim for $\alpha \in (0, \frac{2d}{\gamma ^2})$ (the case $\alpha = 0$ is trivial). For convenience, the constant $C > 0$ below may vary from line to line but its uniformity should be evident from the proof.

Let us start with (36): writing

$$\begin{aligned} M_{\gamma , \epsilon }(B(0, r)) \le M_{\gamma , \epsilon }(B(0, 2\epsilon )) + \sum _{n=0}^{\log \lfloor r/\epsilon \rfloor - 1} M_{\gamma , \epsilon } (A(2^{-n-1}r, 2^{-n}r)) \end{aligned}$$

where

38 $$\begin{aligned}&M_{\gamma , \epsilon } (A(2^{-n-1}r, 2^{-n}r)) := \int _{2^{-n-1}r \le |x| \le 2^{-n}r} e^{\gamma X_\epsilon (x) - \frac{\gamma ^2}{2}\mathbb {E}[X_\epsilon (x)^2]}dx\nonumber \\&\quad = (2^{-n+1}r)^d \int _{\frac{1}{4}\le |u| \le \frac{1}{2}} e^{\gamma X_\epsilon (2^{-n+1}ru) - \frac{\gamma ^2}{2}\mathbb {E}[X_\epsilon (2^{-n+1}ru)^2]}du. \end{aligned}$$

For $C>0$ sufficiently large (but independent of $\epsilon , r, n$), we have

$$\begin{aligned}&\mathbb {E}\left[ X_\epsilon (2^{-n+1}ru)X_\epsilon (2^{-n+1}rv)\right] \\&\quad = -\log \left( |2^{-n+1}r (u-v)| \vee \epsilon \right) + f_\epsilon (2^{-n+1} ru, 2^{-n+1}rv)\\&\quad = -\log (2^{-n+1}r) -\log \left( |u-v| \vee (2^{n-1}\epsilon /r)\right) + f_\epsilon (2^{-n+1} ru, 2^{-n+1}rv)\\&\quad {\left\{ \begin{array}{ll} \le -\log (2^{-n+1}r) + \mathbb {E}\left[ {\overline{X}}_\epsilon (u) {\overline{X}}_\epsilon (v)\right] + C\\ \ge -\log (2^{-n+1} r) - C \end{array}\right. } \qquad \forall u, v \in \left\{ x: \frac{1}{4} \le |x| \le \frac{1}{2}\right\} \end{aligned}$$

where ${\overline{X}}_\epsilon = {\overline{X}} *\nu _\epsilon$ is a mollified exact field (see the first claim in Lemma 7). If we write ${\overline{M}}_{\gamma , \epsilon }$ as the GMC measure associated to ${\overline{X}}_\epsilon$ and also interpret $-\log (2^{-n+1}r)$ as the variance of an independent Gaussian random variable $\mathcal {N}_{n, r}$, then Corollary 2 implies thatwhere the last inequality follows from the uniform integrability of ${\overline{M}}_{\gamma , \epsilon }({\overline{B}}(0, \frac{1}{2}))^\alpha$ (see the second claim in Lemma 7). Substituting this back to (38), we obtain

$$\begin{aligned} \mathbb {E}\left[ M_{\gamma , \epsilon } (A(2^{-n-1}r, 2^{-n}r))^\alpha \right] \le C \left( 2^{-n}r\right) ^{\alpha d + \frac{\gamma ^2}{2}\alpha (1-\alpha )}. \end{aligned}$$

The same principle also shows thatSince $\alpha \in (0, \frac{2d}{\gamma ^2})$ implies $\alpha d + \frac{\gamma ^2}{2} \alpha (1-\alpha ) > 0$, the moment estimates we saw just now are summable: for $\alpha \in (0, 1]$ we have

$$\begin{aligned}&\mathbb {E}\left[ M_{\gamma , \epsilon }(B(0, r))^\alpha \right] \\&\quad \le \mathbb {E}\left[ M_{\gamma , \epsilon }(B(0, 2\epsilon ))^\alpha \right] + \sum _{n=0}^{\log \lfloor r/\epsilon \rfloor - 1} \mathbb {E}\left[ M_{\gamma , \epsilon } (A(2^{-n-1}r, 2^{-n}r))^\alpha \right] \\&\quad \le C \sum _{n \ge 0} \left( 2^{-n}r\right) ^{\alpha d + \frac{\gamma ^2}{2}\alpha (1-\alpha )} \le C r^{\alpha d + \frac{\gamma ^2}{2}\alpha (1-\alpha )} \end{aligned}$$

and the proof for $\alpha \in (1, \frac{2d}{\gamma ^2})$ is similar by considering $\mathbb {E}\left[ M_{\gamma , \epsilon }(B(0, r))^\alpha \right] ^{1/\alpha }$ instead. Thus we have obtained (36).

Next we derive (37) using (36). For $\alpha \in (0, 1]$, we have

$$\begin{aligned} \mathbb {E}\left[ \left( \int _{\epsilon \le |x| \le r} |x|^{-\kappa \gamma }M_{\gamma , \epsilon }(dx)\right) ^\alpha \right]&\le \sum _{n = 0}^{\lfloor \log r/\epsilon \rfloor } \mathbb {E}\left[ \left( \int _{2^n\epsilon \le |x| \le 2^{n+1}\epsilon } |x|^{-\kappa \gamma }M_{\gamma , \epsilon }(dx)\right) ^\alpha \right] \\&\le \sum _{n = 0}^{\lfloor \log r/\epsilon \rfloor } (2^n \epsilon )^{-\kappa \gamma \alpha } \mathbb {E}\left[ M_{\gamma , \epsilon }(B(0, 2^{n+1}\epsilon ))^\alpha \right] \\&\le C \sum _{n = 0}^{\lfloor \log r/\epsilon \rfloor } (2^n \epsilon )^{-\kappa \gamma \alpha } (2^{n+1}\epsilon )^{\alpha d + \frac{\gamma ^2}{2}\alpha (1-\alpha )}\\&\le C' \epsilon ^{\alpha (d-\kappa \gamma ) + \frac{\gamma ^2}{2}\alpha (1-\alpha )} \frac{(r / \epsilon )^{\alpha (d-\kappa \gamma ) + \frac{\gamma ^2}{2}\alpha (1-\alpha )} - 1}{2^{\alpha (d-\kappa \gamma ) + \frac{\gamma ^2}{2}\alpha (1-\alpha )} - 1} \end{aligned}$$

which leads to the bound (37). As for $\alpha > 1$ the inequality again follows from similar arguments applied to $\mathbb {E}\left[ \left( \int _{\epsilon \le |x| \le r} |x|^{-\kappa \gamma }M_{\gamma , \epsilon }(dx)\right) ^\alpha \right] ^{1/\alpha }$. $\square$

The next lemma is an elementary estimate that allows us to remove irrelevant mass from our asymptotic analysis in Section 3.2.1.

#### Lemma 9

Let $k > 0$, and *A*, *B* be two non-negative random variables with finite *k*-th moment.

(i) Suppose there exists some $\eta > 0$ such that $\mathbb {E}\left[ (A+B)^k\right] \le (1+\eta ) \mathbb {E}[B^k]$, then
  $$\begin{aligned} \mathbb {E}[A^k] \le \max \left( \eta , \left( \eta / k\right) ^k\right) \mathbb {E}[B^k]. \end{aligned}$$
(ii) Suppose there exists some $\eta > 0$ such that $\mathbb {E}[A^k] \le \eta \mathbb {E}[B^k]$, then
  $$\begin{aligned} \mathbb {E}\left[ (A+B)^k\right] \le \left[ 1 + \max \left( \eta , k2^k \eta ^{\frac{1}{k}}\right) \right] \mathbb {E}[B^k]. \end{aligned}$$

#### Proof

We begin with the first statement. For $k \ge 1$, one can easily check (say by computing the first derivative) that

$$\begin{aligned} (A+B)^k - B^k - A^k \ge 0 \qquad \forall A, B \ge 0 \end{aligned}$$

and so $\mathbb {E}[A^k + B^k] \le (1+\eta ) \mathbb {E}[B^k]$ by assumption and hence $\mathbb {E}[A^k] \le \eta \mathbb {E}[B^k]$. As for $k \in (0, 1)$,

$$\begin{aligned} (A+B)^k - B^k = k \int _B^{A+B} x^{k-1}dx \ge kAB^{k-1} \end{aligned}$$

and thus

$$\begin{aligned} \eta \mathbb {E}[B^k] \ge \mathbb {E}[(A+B)^k] - \mathbb {E}[B^k] \ge k \mathbb {E}[AB^{k-1}] \ge k \mathbb {E}[A^k]^{\frac{1}{k}} \mathbb {E}[B^k]^{\frac{k-1}{k}} \end{aligned}$$

by (reverse) Hölder’s inequality. Rearranging terms, we arrive at $\mathbb {E}[A^k] \le (\eta / k)^k \mathbb {E}[B^k]$.

Now for the second statement, we start with $k \in (0, 1)$ where

$$\begin{aligned} \mathbb {E}[(A+B)^k] \le \mathbb {E}[A^k] + \mathbb {E}[B^k] \le (1+\eta ) \mathbb {E}[B^k] \end{aligned}$$

by our assumption. As for $k \ge 1$, we have

$$\begin{aligned} (A+B)^k - B^k = k \int _B^{A+B} x^{k-1} dx \le kA(A+B)^{k-1} \le k2^{k-1} \left( A^k + AB^{k-1}\right) \end{aligned}$$

from which we obtain

$$\begin{aligned} \mathbb {E}[(A+B)^k]&\le \mathbb {E}[B^k] + k2^{k-1}\left( \mathbb {E}[A^k] + \mathbb {E}[AB^{k-1}]\right) \\&\le \mathbb {E}[B^k] + k2^{k-1}\mathbb {E}[A^k] + k2^{k-1} \mathbb {E}[A^k]^{\frac{1}{k}}\mathbb {E}[B^{k}]^{\frac{k-1}{k}}\\&\le \left[ 1 + k2^{k-1} \left( \eta + \eta ^{\frac{1}{k}} \right) \right] \mathbb {E}[B^k] \end{aligned}$$

by an application of Hölder’s inequality. This concludes the proof. $\square$

## Main Proofs

For convenience, we shall write $p = p_c = 2d/\gamma ^2$ throughout the entire Section 3.

### Warm-up: a limit from below

As advertised in Section 1.5, the proof of Theorem 1 largely boils down to the analysis of positive moments of the exponential functional of Brownian motion with negative drift, with the help of Williams’ path decomposition.

To offer a first glance at how some of the results in Section 2.2 may be applied, we make a digression and study the limit of a much simpler but related quantity. The following result, while not needed for the proof of Theorem 1, will be used when we sketch a renormalisation result in Appendix B.

#### Lemma 10

Let ${\overline{X}}$ be the exact field on the closed unit ball. If $\gamma \in (0, \sqrt{2d})$ and $p = \frac{2d}{\gamma ^2}$, then for any $r \in (0, 1]$,

39 $$\begin{aligned}&\lim _{\alpha \rightarrow (p-1)^-} (p-1 - \alpha )\mathbb {E}\left[ \left( \int _{|x|\le r} e^{\gamma {\overline{X}}(x) - \frac{\gamma ^2}{2} \mathbb {E}[{\overline{X}}(x)^2]}\frac{dx}{|x|^{\gamma ^2}} \right) ^{\alpha } \right] \nonumber \\&\qquad = (p-1) \mathbb {E}\left[ \left( \int _{-\infty }^\infty e^{-\gamma \mathcal {B}_t^\mu } Z_\gamma (dt)\right) ^{p-1} \right] \end{aligned}$$

where $Z_{\gamma }(dt)$ is the GMC associated to ${\widehat{X}}$ as defined in (29) and (30).

The fact that (39) is independent of $r \in (0, 1)$ follows from the same reasoning as in Remark 4. Indeed for $0< \alpha < p-1 \le 1$, we have

$$\begin{aligned}&\mathbb {E}\left[ \left( \int _{|x|\le r} e^{\gamma {\overline{X}}(x) - \frac{\gamma ^2}{2} \mathbb {E}[{\overline{X}}(x)^2]}\frac{dx}{|x|^{\gamma ^2}} \right) ^{\alpha } \right] \\&\qquad \le \mathbb {E}\left[ \left( \int _{|x|\le 1} e^{\gamma {\overline{X}}(x) - \frac{\gamma ^2}{2} \mathbb {E}[{\overline{X}}(x)^2]}\frac{dx}{|x|^{\gamma ^2}} \right) ^{\alpha } \right] \\&\qquad \le \mathbb {E}\left[ \left( \int _{|x|\le r} e^{\gamma {\overline{X}}(x) - \frac{\gamma ^2}{2} \mathbb {E}[{\overline{X}}(x)^2]}\frac{dx}{|x|^{\gamma ^2}} \right) ^{\alpha } \right] + r^{-\gamma ^2 \alpha } \mathbb {E}\left[ {\overline{M}}_{\gamma }({\overline{B}}(0, 1))^\alpha \right] \end{aligned}$$

where ${\overline{M}}_{\gamma }$ is the GMC measure associated to ${\overline{X}}$. The last term on the RHS can be bounded uniformly in $\alpha \le p-1$ by the existence of GMC moments (Lemma 7), and hence does not affect the limit in (39). The case $p - 1 > 1$ is similar. For this reason, we shall assume for simplicity that $r=1$ in the proof below.

#### Proof

Starting with the radial decomposition of our exact field

$$\begin{aligned} \mathbb {E}\left[ {\overline{X}}(x) {\overline{X}}(y)\right] =: \mathbb {E}[B_{-\log |x|} B_{-\log |y|}] + \mathbb {E}\left[ {\widehat{X}}(x) {\widehat{X}}(y)\right] , \end{aligned}$$

we consider the change of variable $x = e^{-t}$ for $d=1$ and $x = e^{-t} e^{i\theta }$ for $d=2$ and rewrite the $\alpha$-th moment as

$$\begin{aligned} \mathbb {E}\left[ \left( \int _{|x|\le 1} e^{\gamma {\overline{X}}(x) - \frac{\gamma ^2}{2} \mathbb {E}[{\overline{X}}(x)^2]}\frac{dx}{|x|^{\gamma ^2}} \right) ^{\alpha } \right] = \mathbb {E}\left[ \left( \int _0^\infty e^{\gamma \left( B_t -\mu t\right) }Z_\gamma (dt)\right) ^{\alpha }\right] \end{aligned}$$

with $\mu = \frac{\gamma }{2}(p-1)$. The next step is to re-express $(B_t - \mu t)_{t \ge 0}$ using Lemma 4 and Theorem 4. If $\phi _c: x \mapsto x + c$ is the shift operator, we have

$$\begin{aligned} \mathbb {E}\left[ \left( \int _0^\infty e^{\gamma \left( B_t -\mu t\right) }Z_\gamma (dt)\right) ^{\alpha }\right]&= \mathbb {E}\left[ \left( \int _{-L_{\mathcal {M}}}^\infty e^{\gamma (\mathcal {M}-\mathcal {B}_t^\mu )}Z_\gamma \circ \phi _{L_{\mathcal {M}}} (dt)\right) ^{\alpha }\right] \\&= \mathbb {E}\left[ \left( \int _{-L_{\mathcal {M}}}^\infty e^{\gamma (\mathcal {M}-\mathcal {B}_t^\mu )}Z_\gamma (dt)\right) ^{\alpha }\right] \end{aligned}$$

where $(\mathcal {B}_{\pm t}^\mu )_{t \ge 0}$ are two independent Brownian motions with drift $\mu$ conditioned to stay positive, $\mathcal {M}$ is an independent $\mathrm {Exp}(2\mu )$ variable, and $L_{\mathcal {M}} := \sup \{t > 0: \mathcal {B}_{-t}^\mu = \mathcal {M}\}$. Note that the removal of $\phi _{\mathcal {M}}$ in the last equality above follows from the translation invariance of $Z_{\gamma }(dt)$, which is inherited from the scale invariance of ${\widehat{X}}(x)$ (see Lemma 5).

Now for any $x > 0$, we have

$$\begin{aligned} \mathbb {E}\left[ \left( \int _{-L_{x}}^\infty e^{\gamma (\mathcal {M}-\mathcal {B}_t^\mu )}Z_\gamma (dt)\right) ^{\alpha } 1_{\{\mathcal {M}\ge x\}}\right]&\le \mathbb {E}\left[ \left( \int _{-L_{\mathcal {M}}}^\infty e^{\gamma (\mathcal {M}-\mathcal {B}_t^\mu )}Z_\gamma (dt)\right) ^{\alpha }\right] \\&\le \mathbb {E}\left[ \left( \int _{-\infty }^\infty e^{\gamma (\mathcal {M}-\mathcal {B}_t^\mu )}Z_\gamma (dt)\right) ^{\alpha }\right] . \end{aligned}$$

It is enough to focus on the lower bound since along the way we will see that the upper bound is tight as $\alpha \rightarrow (p-1)^-$. By independence, we compute

$$\begin{aligned}&\mathbb {E}\left[ \left( \int _{-L_{x}}^\infty e^{\gamma (\mathcal {M}-\mathcal {B}_t^\mu )}Z_\gamma (dt)\right) ^{\alpha } 1_{\{\mathcal {M}\ge x\}}\right] \\&\qquad = \mathbb {E}\left[ \left( \int _{-L_{x}}^\infty e^{-\gamma \mathcal {B}_t^\mu }Z_\gamma (dt)\right) ^{\alpha } \right] \int _x^\infty e^{\gamma \alpha m} 2\mu e^{-2\mu m} dm\\&\qquad = \mathbb {E}\left[ \left( \int _{-L_{x}}^\infty e^{-\gamma \mathcal {B}_t^\mu }Z_\gamma (dt)\right) ^{\alpha } \right] \frac{2\mu }{2\mu - \gamma \alpha } e^{-(2\mu - \gamma \alpha ) x}. \end{aligned}$$

Substituting $2 \mu = \gamma (p-1)$ back to our expression, we have

$$\begin{aligned}&\liminf _{\alpha \rightarrow (p-1)^-} (p-1 - \alpha ) \mathbb {E}\left[ \left( \int _{-L_{x}}^\infty e^{\gamma (\mathcal {M}-\mathcal {B}_t^\mu )}Z_\gamma (dt)\right) ^{\alpha } 1_{\{\mathcal {M}\ge x\}}\right] \\&\qquad \ge (p-1) \mathbb {E}\left[ \left( \int _{-L_{x}}^\infty e^{-\gamma \mathcal {B}_t^\mu }Z_\gamma (dt)\right) ^{p-1} \right] . \end{aligned}$$

As $x > 0$ is arbitrary, we let $x \rightarrow \infty$ so that $L_{x} \rightarrow \infty$ almost surely and the lower bound matches the upper bound. This concludes the proof. $\square$

### Proof of Theorem 1

Recall that $X_\epsilon = X *\nu _\epsilon$ for some mollifier $\nu \in C_c^\infty (\mathbb {R}^d)$, and we shall assume without loss of generality that $\mathrm {supp}(\nu ) \subset B(0, \frac{1}{4})$. The proof of our main theorem consists of three parts:

1. We first remove part of the mass in $M_{\gamma , \epsilon }(D)$ which does not contribute to the blow-up.
2. We then argue that the problem may be reduced to one regarding the exact field (28).
3. Finally, we prove that the *p*-th moment, after suitable renormalisation, converges to the probabilistic representation (35) of the reflection coefficient of GMC as $\epsilon \rightarrow 0^+$.

According to Lemma 7, the covariance structure of $X_\epsilon$ is of the form

40 $$\begin{aligned} \mathbb {E}[X_\epsilon (x) X_\epsilon (y)] = -\log \left( |x-y| \vee \epsilon \right) + f_\epsilon (x, y) \end{aligned}$$

where $\sup _{\epsilon > 0} \sup _{x, y \in D} |f_\epsilon (x, y)| < \infty$. In the following we shall assume without loss of generality that $f, f_\epsilon \ge 0$ everywhere in the domain. Indeed:

$$\begin{aligned} \mathbb {E}[X(x) X(y)]&= -\log |x-y| + f(x, y) \\&= -\log |r^{-1}x- r^{-1}y| - \log r + f(x, y) \end{aligned}$$

and since $-\log r + f(x, y) \ge 0$ uniformly for *r* sufficiently small, we can always rewrite our expression in terms of another log-correlated field (by scaling our coordinate with *r*) with an ‘*f*’-function that satisfies the non-negativity condition, i.e. $X^{(r)}(\cdot ) := X(r \cdot )$ on the new domain *D*/*r* with

$$\begin{aligned} \mathbb {E}\left[ X^{(r)}(x) X^{(r)}(y)\right] = \mathbb {E}\left[ X(rx) X(ry)\right] = -\log |x-y| + \underbrace{f(rx, ry) - \log r}_{=:f_r(x, y)}. \end{aligned}$$

The same argument also applies to $f_\epsilon$, and moreover we have

$$\begin{aligned} X_\epsilon (rx)&:= \int X(rx - \epsilon u) \nu (u) du = \int X^{(r)}(x - \tfrac{\epsilon }{r}u) \nu (u) du = X^{(r)}_{\epsilon / r}(u). \end{aligned}$$

The reduction to the case where $f, f_\epsilon \ge 0$ everywhere is only reasonable if Theorem 1 is invariant under the rescaling of space, which we quickly verify now. If we rewrite

$$\begin{aligned}&\mathbb {E}\left[ \left( \int _D g(x) M_{\gamma , \epsilon }(dx)\right) ^{p}\right] = \mathbb {E}\left[ \left( \int _D g(x) e^{\gamma X_{\epsilon }(x) - \frac{\gamma ^2}{2}\mathbb {E}[X_\epsilon (x)^2]} dx\right) ^{p}\right] \\&\qquad = \mathbb {E}\left[ \left( \int _{D/r} r^dg(ru) e^{\gamma X_{\epsilon }(ru) - \frac{\gamma ^2}{2}\mathbb {E}[X_\epsilon (ru)^2]} du\right) ^{p}\right] \\&\qquad = \mathbb {E}\left[ \left( \int _{D/r} g_r(u) e^{\gamma X_{\epsilon /r}^{(r)}(u)- \frac{\gamma ^2}{2}\mathbb {E}[X_{\epsilon /r}^{(r)}(u)]} du\right) ^{p}\right] \end{aligned}$$

where $g_r(u) := r^d g(ru)$ still satisfies the non-negativity and continuity assumptions, then Theorem 1 leads to the asymptotics (as $\epsilon \rightarrow 0^+$)

$$\begin{aligned}&\left( \int _{D/r} e^{d(p-1) f_r(u, u)} g_r(u)^{p} du\right) \frac{\gamma ^2}{2}(p-1)^2 {\overline{C}}_{\gamma , d}\log \frac{1}{\epsilon /r}\\&\quad =\left( \int _{D} e^{d(p-1) [f(x, x) - \log r]} (r^dg(x))^{p} d(x/r)\right) \frac{\gamma ^2}{2}(p-1)^2 {\overline{C}}_{\gamma , d}\log \frac{1}{\epsilon /r}\\&\quad =\left( \int _{D} e^{d(p-1) f(x, x)]} g(x)^{p} dx\right) \frac{\gamma ^2}{2}(p-1)^2 {\overline{C}}_{\gamma , d}\log \frac{r}{\epsilon } \end{aligned}$$

which is the desired result (since $r > 0$ is fixed and its appearance in the logarithm does not affect the leading order behaviour as $\epsilon \rightarrow 0$).

For simplicity our proof below treats the case where $g \equiv 1$ on ${\overline{D}}$, even though the analysis can easily be adapted to treat continuous $g \ge 0$ (see Remark 5 before Section 3.3).

#### Pre-processing: removal of irrelevant mass

Let us recall that $p = 2d/\gamma ^2 > 1$. Then

$$\begin{aligned} \mathbb {E}\left[ M_{\gamma , \epsilon }(D)^p\right]&= \mathbb {E}\left[ \left( \int _D e^{\gamma X_{\epsilon }(x) - \frac{\gamma ^2}{2} \mathbb {E}[X_\epsilon (x)^2]} dx\right) ^p\right] \\&= \int _D du\mathbb {E}\left[ e^{\gamma X_{\epsilon }(u) - \frac{\gamma ^2}{2} \mathbb {E}[X_\epsilon (u)^2]}\left( \int _D e^{\gamma X_{\epsilon }(x) - \frac{\gamma ^2}{2} \mathbb {E}[X_\epsilon (x)^2]} dx\right) ^{p-1}\right] \\&= \int _D du\mathbb {E}\left[ \left( \int _D \frac{e^{\gamma ^2 f_\epsilon (x, u)}}{\left( |x-u|\vee \epsilon \right) ^{\gamma ^2}} e^{\gamma X_{\epsilon }(x) - \frac{\gamma ^2}{2} \mathbb {E}[X_\epsilon (x)^2]} dx\right) ^{p-1}\right] \\ \end{aligned}$$

where the last equality follows from Cameron–Martin theorem (see Lemma 1) and the fact that

$$\begin{aligned} e^{\gamma ^2 \mathbb {E}[X_\epsilon (x) X_\epsilon (u)]} = \frac{e^{\gamma ^2f_\epsilon (x, u)}}{(|x-u| \vee \epsilon )^{\gamma ^2}} \end{aligned}$$

based on the expression (40). We shall therefore fix $u \in D$ and consider the integrand

41 $$\begin{aligned} \mathbb {E}\left[ \left( \int _D \frac{e^{\gamma ^2 f_\epsilon (x, u)}}{\left( |x-u|\vee \epsilon \right) ^{\gamma ^2}} e^{\gamma X_{\epsilon }(x) - \frac{\gamma ^2}{2} \mathbb {E}[X_\epsilon (x)^2]} dx\right) ^{p-1}\right] . \end{aligned}$$

To proceed, we use the fact (which is a consequence of Lemma 9) that we can remove any mass inside the integral in (41) provided that they do not contribute to the leading order blow-up. The part we want to remove is

$$\begin{aligned} B_\epsilon := \int _{D \cap B(u, K\epsilon )} \frac{e^{\gamma ^2 f_\epsilon (x, u)}}{\left( |x-u|\vee \epsilon \right) ^{\gamma ^2}} e^{\gamma X_{\epsilon }(x) - \frac{\gamma ^2}{2} \mathbb {E}[X_\epsilon (x)^2]} dx \end{aligned}$$

for some $K > 1$. For this we need to show that the $(p-1)$-th moment remains bounded when $\epsilon \rightarrow 0$, and this is true because

$$\begin{aligned} \mathbb {E}\left[ B_\epsilon ^{p-1} \right]&\le C\epsilon ^{-\gamma ^2(p-1)} \mathbb {E}\left[ M_{\gamma , \epsilon }(D \cap B(u, K \epsilon ))^{p-1} \right] \\&\le C \epsilon ^{-\gamma ^2(p-1)} (K\epsilon )^{d(p-1) + \frac{\gamma ^2}{2}[(p-1)-(p-1)^2]} \le C K^{\gamma ^2(p-1)} \end{aligned}$$

where the first inequality follows from the fact that $f_\epsilon$ may be uniformly bounded (see Lemma 7), and the second inequality from the first estimate in Lemma 8.

Actually we will remove more mass from our analysis. Let $r > 0$ be some fixed number, then of course we have

$$\begin{aligned}&\mathbb {E}\left[ \left( \int _{D \cap \{|x-u| > r\}} \frac{e^{\gamma ^2 f_\epsilon (x, u)}}{\left( |x-u|\vee \epsilon \right) ^{\gamma ^2}} e^{\gamma X_{\epsilon }(x) - \frac{\gamma ^2}{2} \mathbb {E}[X_\epsilon (x)^2]} dx\right) ^{p-1}\right] \\&\qquad \le C r^{-(p-1)\gamma ^2} \mathbb {E}\left[ \left( M_{\gamma , \epsilon }(D)\right) ^{p-1}\right] \end{aligned}$$

where the RHS stays bounded as $\epsilon \rightarrow 0$, by the convergence and existence of $(p-1)$-th moment of Gaussian multiplicative chaos (see Lemmas 7 and 8). Summarising the work so far, we have

##### Lemma 11

As $$\epsilon \rightarrow 0^+$$,

$$\begin{aligned} \mathbb {E}\left[ M_{\gamma , \epsilon }(D)^p\right]&= \int _D du\mathbb {E}\left[ \left( \int _{D \cap \{K\epsilon \le |x-u| \le r\}} \frac{e^{\gamma ^2 f_\epsilon (x, u)}}{|x-u|^{\gamma ^2}} e^{\gamma X_{\epsilon }(x) - \frac{\gamma ^2}{2} \mathbb {E}[X_\epsilon (x)^2]} dx\right) ^{p-1}\right] \\&\qquad + \mathcal {O}(1). \end{aligned}$$

#### Reduction to exact fields

The next step is to rewrite

42 $$\begin{aligned} \mathbb {E}\left[ \left( \int _{D \cap \{K\epsilon \le |x-u| \le r\}} \frac{e^{\gamma ^2 f_\epsilon (x, u)}}{|x-u|^{\gamma ^2}} e^{\gamma X_{\epsilon }(x) - \frac{\gamma ^2}{2} \mathbb {E}[X_\epsilon (x)^2]} dx\right) ^{p-1}\right] \end{aligned}$$

in terms of something more analytically tractable. We claim that

##### Lemma 12

There exist some constants $C_{K, r}(\epsilon ) > 0$ independent of $u \in D$ such that

$$\begin{aligned} \limsup _{r \rightarrow 0} \limsup _{K \rightarrow \infty } \limsup _{\epsilon \rightarrow 0} C_{K, r}(\epsilon ) = 0 \end{aligned}$$

and that

43 $$\begin{aligned}&\mathbb {E}\left[ \left( \int _{D \cap \{K\epsilon \le |x-u| \le r\}} \frac{e^{\gamma ^2 f_\epsilon (x, u)}}{|x-u|^{\gamma ^2}} e^{\gamma X_{\epsilon }(x) - \frac{\gamma ^2}{2} \mathbb {E}[X_\epsilon (x)^2]} dx\right) ^{p-1}\right] \nonumber \\&\qquad = \left( 1+ \mathcal {O}(C_{K, r}(\epsilon ))\right) e^{d(p-1) f(u,u)}\nonumber \\&\qquad \qquad \times \mathbb {E}\left[ \left( \int _{(D- u)\cap \{K\epsilon \le |x| \le r\}} e^{\gamma {\overline{X}}_{\epsilon }(x)- \frac{\gamma ^2}{2} \mathbb {E}[{\overline{X}}_{\epsilon }(x)^2]}\frac{dx}{|x|^{\gamma ^2}} \right) ^{p-1}\right] . \end{aligned}$$

##### Proof

Recall our earlier reduction to the case where $f, f_\epsilon \ge 0$ everywhere, and without loss of generality suppose $r \le 1/4$. We consider a log-correlated random field ${\overline{X}}^{(u)}(\cdot )$ on *B*(*u*, 4*r*) with covariance

$$\begin{aligned} \mathbb {E}\left[ {\overline{X}}^{(u)}(x) {\overline{X}}^{(u)}(y)\right] = - \log |(x-u) - (y-u)| = -\log |x-y| \quad \forall x, y \in B(u, 4r). \end{aligned}$$

This field can be easily realised as the exact field in (28) up to a shift of domain, i.e. ${\overline{X}}^{(u)}(\cdot ) := {\overline{X}}(\cdot - u)$. If we write ${\overline{X}}^{(u)}_{\epsilon } = {\overline{X}}^{(u)} *\nu _\epsilon$, then we have

$$\begin{aligned} \mathbb {E}\left[ {\overline{X}}^{(u)}_{\epsilon }(x) {\overline{X}}^{(u)}_{\epsilon } (y) \right]&= \int \int \left( - \log |(x-u -\epsilon s) - (y-u - \epsilon t)|\right) \nu (s) \nu (t) ds dt\\&= \int \int \left( - \log |(x -\epsilon s) - (y - \epsilon t)|\right) \nu (s) \nu (t) ds dt. \end{aligned}$$

Similarly, if $X_{\epsilon } = X *\nu _\epsilon$, then

$$\begin{aligned} \mathbb {E}\left[ X_\epsilon (x) X_\epsilon (y)\right] {=} \int \int \left[ - \log |(x-\epsilon s) {-} (y-\epsilon t)| {+} f(x - \epsilon s, y - \epsilon t) \right] \nu (s) \nu (t) ds dt. \end{aligned}$$

Since *f* is continuous (and hence uniformly continuous) on the compact set ${\overline{D}} \times {\overline{D}}$, it has a modulus of continuity, say $\omega (\cdot )$. If $\mathcal {N}_{u} \sim \mathcal {N}(0, f(u,u))$ is independent of everything else, then

$$\begin{aligned}&\left| \mathbb {E}\left[ ({\overline{X}}^{(u)}_\epsilon (x)+\mathcal {N}_u)({\overline{X}}^{(u)}_\epsilon (y)+\mathcal {N}_u)\right] - \mathbb {E}\left[ X_\epsilon (x) X_\epsilon (y)\right] \right| \\&=\left| \mathbb {E}\left[ {\overline{X}}^{(u)}_\epsilon (x){\overline{X}}^{(u)}_\epsilon (y)\right] + f(u, u) - \mathbb {E}\left[ X_\epsilon (x) X_\epsilon (y)\right] \right| \\&= \left| \int \int [f(u, u) - f(x-\epsilon s, y - \epsilon t)] \nu (s) \nu (t) ds dt \right| \le 2\omega (\epsilon ) + 2\omega (r) \end{aligned}$$

for all $x, y \in D \cap \{v: K\epsilon \le |v-u| \le r\}$, and this upper bound goes to 0 as $\epsilon , r \rightarrow 0^+$. Applying Corollary 2, we can perform a Gaussian comparison and obtain the estimate

$$\begin{aligned}&\frac{\mathbb {E}\left[ \left( \int _{D \cap \{K\epsilon \le |x-u| \le r\}} \frac{e^{\gamma ^2 f_\epsilon (x, u)}}{|x-u|^{\gamma ^2}} e^{\gamma ({\overline{X}}_{\epsilon }^{(u)}(x)+\mathcal {N}_u) - \frac{\gamma ^2}{2} \mathbb {E}[({\overline{X}}^{(u)}_{\epsilon }(x)+\mathcal {N}_u)^2]} dx\right) ^{p-1}\right] }{\mathbb {E}\left[ \left( \int _{D \cap \{K\epsilon \le |x-u| \le r\}} \frac{e^{\gamma ^2 f_\epsilon (x, u)}}{|x-u|^{\gamma ^2}} e^{\gamma X_{\epsilon }(x) - \frac{\gamma ^2}{2} \mathbb {E}[X_\epsilon (x)^2]} dx\right) ^{p-1}\right] } \\&\qquad = 1+ \mathcal {O}(\omega (\epsilon ) + \omega (r)) \end{aligned}$$

uniformly in $u \in D$, and so it suffices to consider the numerator

44 $$\begin{aligned}&\mathbb {E}\left[ \left( \int _{D \cap \{K\epsilon \le |x-u| \le r\}} \frac{e^{\gamma ^2 f_\epsilon (x, u)}}{|x-u|^{\gamma ^2}} e^{\gamma ({\overline{X}}^{(u)}_{\epsilon }(x)+\mathcal {N}_u) - \frac{\gamma ^2}{2} \mathbb {E}[({\overline{X}}^{(u)}_{\epsilon }(x)+\mathcal {N}_u)^2]}dx\right) ^{p-1}\right] . \end{aligned}$$

We note the following observations:

- $\mathcal {N}_u$ is independent of ${\overline{X}}^{(u)}$ and its expectation may be evaluated directly.
- We may replace $e^{\gamma ^2 f_\epsilon (x, u)}$ by $e^{\gamma ^2 f(u,u)}$ and pull it out of the expectation, up to some multiplicative error which vanishes as $\epsilon \rightarrow 0$, $K \rightarrow \infty$ and $r \rightarrow 0$. This is true because
  $$\begin{aligned} |f_\epsilon (x, u) - f(u, u)| \le |f_\epsilon (x, u) - f(x, u)| + \omega (r), \end{aligned}$$
  and for $$|x-u| \ge K\epsilon \ge \epsilon $$:
  $$\begin{aligned}&\left| f_\epsilon (x, u) - f(x, u)\right| =\left| \mathbb {E}[X_\epsilon (x) X_\epsilon (u)] - \mathbb {E}[X(x) X(u)]\right| \\&\quad = \int \int \left[ -\log \frac{|(x-\epsilon s) - (u - \epsilon t)|}{|x-u|} +f(x - \epsilon s, u - \epsilon t) - f(x,u)\right] \nu (s) \nu (t) ds dt\\&\quad \le \int \int \left| \log \left( 1 - \epsilon \frac{s-t}{|x-u|}\right) \right| \nu (s)\nu (t) ds dt + 2\omega (\epsilon ) \le \frac{1}{K} + 2 \omega (\epsilon ) \end{aligned}$$
  where the last inequality follows from the assumption that $\mathrm {supp}(\nu ) \subset B(0, \frac{1}{4})$.

Taking all these considerations together, (44) can be rewritten (up to a multiplicative error of $C_{K, r}(\epsilon )$ as defined in the statement of the lemma) as

$$\begin{aligned} \nonumber&e^{\gamma ^2(p-1) f(u,u)}\mathbb {E}\left[ \left( e^{\gamma \mathcal {N}_u - \frac{\gamma ^2}{2} \mathbb {E}[\mathcal {N}_u^2]}\right) ^{p-1}\right] \\ \nonumber&\qquad \qquad \times \mathbb {E}\left[ \left( \int _{D \cap \{K\epsilon \le |x-u| \le r\}} \frac{e^{\gamma {\overline{X}}^{(u)}_{\epsilon }(x)- \frac{\gamma ^2}{2} \mathbb {E}[{\overline{X}}^{(u)}_{\epsilon }(x)^2]}dx}{|x-u|^{\gamma ^2}} \right) ^{p-1}\right] \\&= e^{d(p-1) f(u,u)}\mathbb {E}\left[ \left( \int _{(D-u) \cap \{K\epsilon \le |x| \le r\}} e^{\gamma {\overline{X}}_{\epsilon }(x)- \frac{\gamma ^2}{2} \mathbb {E}[{\overline{X}}_{\epsilon }(x)^2]}\frac{dx}{|x|^{\gamma ^2}} \right) ^{p-1}\right] \end{aligned}$$

since $${\overline{X}}_\epsilon ^{(u)}(\cdot ) = {\overline{X}}^{(u)} *\nu _\epsilon = {\overline{X}}(\cdot - u) *\nu _\epsilon = {\overline{X}}_\epsilon (\cdot - u)$$. $$\square $$

#### Convergence to the reflection coefficient

The final ingredient we need is the following asymptotic formula.

##### Lemma 13

We have

45 $$\begin{aligned}&\mathbb {E}\left[ \left( \int _{K\epsilon \le |x| \le r} e^{\gamma {\overline{X}}_{\epsilon }(x)- \frac{\gamma ^2}{2} \mathbb {E}[{\overline{X}}_{\epsilon }(x)^2]}\frac{dx}{|x|^{\gamma ^2}} \right) ^{p-1}\right] \nonumber \\&\qquad \overset{\epsilon \rightarrow 0^+}{\sim } \frac{\gamma ^2}{2}(p-1)^2 \mathbb {E}\left[ \left( \int _{-\infty }^\infty e^{-\gamma \mathcal {B}_t^\mu } Z_\gamma (dt)\right) ^{p-1}\right] \log \frac{1}{\epsilon }. \end{aligned}$$

##### Proof of Theorem 1

Note that for any $u \in D$, we have the trivial uniform bound

$$\begin{aligned}&\mathbb {E}\left[ \left( \int _{(D- u)\cap \{K\epsilon \le |x| \le r\}} e^{\gamma {\overline{X}}_{\epsilon }(x)- \frac{\gamma ^2}{2} \mathbb {E}[{\overline{X}}_{\epsilon }(x)^2]}\frac{dx}{|x|^{\gamma ^2}} \right) ^{p-1}\right] \\&\qquad \le \mathbb {E}\left[ \left( \int _{\{K\epsilon \le |x| \le r\}} e^{\gamma {\overline{X}}_{\epsilon }(x)- \frac{\gamma ^2}{2} \mathbb {E}[{\overline{X}}_{\epsilon }(x)^2]}\frac{dx}{|x|^{\gamma ^2}} \right) ^{p-1}\right] \end{aligned}$$

the asymptotics of which is given by Lemma 13. On the other hand, since *D* is open, there exists ${\tilde{r}} = {\tilde{r}}(u) > 0$ sufficiently small (but not uniform in $u \in D$) such that $(D- u)\cap \{K\epsilon \le |x| \le {\tilde{r}}\} = \{K\epsilon \le |x| \le {\tilde{r}}\}$. By dominated convergence, this means that

$$\begin{aligned}&\lim _{\epsilon \rightarrow 0^+} \frac{1}{-\log \epsilon } \int _D du \Bigg \{\mathbb {E}\left[ \left( \int _{\{K\epsilon \le |x| \le r\}} e^{\gamma {\overline{X}}_{\epsilon }(x)- \frac{\gamma ^2}{2} \mathbb {E}[{\overline{X}}_{\epsilon }(x)^2]}\frac{dx}{|x|^{\gamma ^2}} \right) ^{p-1}\right] \\&\quad -\mathbb {E}\left[ \left( \int _{(D- u)\cap \{K\epsilon \le |x| \le r\}} e^{\gamma {\overline{X}}_{\epsilon }(x)- \frac{\gamma ^2}{2} \mathbb {E}[{\overline{X}}_{\epsilon }(x)^2]}\frac{dx}{|x|^{\gamma ^2}} \right) ^{p-1}\right] \Bigg \} = 0. \end{aligned}$$

Combining this with Lemmas 11, 12 and again 13, we see that both

$$\begin{aligned} \liminf _{\epsilon \rightarrow 0^+} \frac{1}{-\log \epsilon } \mathbb {E}\left[ M_{\gamma , \epsilon }(D)^p\right] \qquad \text {and} \qquad \limsup _{\epsilon \rightarrow 0^+} \frac{1}{-\log \epsilon } \mathbb {E}\left[ M_{\gamma , \epsilon }(D)^p\right] \end{aligned}$$

are equal to

$$\begin{aligned}&\left( 1+ \mathcal {O}\left( \limsup _{\epsilon \rightarrow 0^+} C_{K, r}(\epsilon )\right) \right) \left( \int _D e^{d(p-1) f(u,u)} du\right) \\&\qquad \qquad \times \frac{\gamma ^2}{2}(p-1)^2 \mathbb {E}\left[ \left( \int _{-\infty }^\infty e^{-\gamma \mathcal {B}_t^\mu } Z_\gamma (dt)\right) ^{p-1}\right] . \end{aligned}$$

Since $K, r > 0$ are arbitrary, we send $K \rightarrow \infty$ and $r \rightarrow 0$, and conclude our proof by identifying

$$\begin{aligned} \mathbb {E}\left[ \left( \int _{-\infty }^\infty e^{-\gamma \mathcal {B}_t^\mu } Z_\gamma (dt)\right) ^{p-1}\right] \end{aligned}$$

as the probabilistic representation of ${\overline{C}}_{\gamma , d}$ with Lemma 6. $\square$

The rest of the section is to prove Lemma 13, which is broken into several steps.

*Step 1: simplifying the radial part.* As in the proof of Lemma 10, we start by considering the radial decomposition of the exact field ${\overline{X}}$ and extend it to its regularised version, i.e.

$$\begin{aligned} {\overline{X}}_{\epsilon }(x) = B_{\epsilon , -\log |x|} + {\widehat{X}}_\epsilon (x) \end{aligned}$$

where $B_{\epsilon , -\log |x|} =( B_{-\log |\cdot |} *\nu _\epsilon )(x)$ and ${\widehat{X}}_{\epsilon }(x) = {\widehat{X}} *\nu _{\epsilon }(x)$. By performing the substitution $x = e^{-t}$ when $d=1$ or $x=e^{-t} e^{i\theta }$ when $d=2$, we obtain

$$\begin{aligned}&\mathbb {E}\left[ \left( \int _{K\epsilon \le |x| \le r} e^{\gamma {\overline{X}}_{\epsilon }(x)- \frac{\gamma ^2}{2} \mathbb {E}[{\overline{X}}_{\epsilon }(x)^2]}\frac{dx}{|x|^{\gamma ^2}} \right) ^{p-1}\right] \\&\qquad = \mathbb {E}\left[ \left( \int _{-\log r}^T e^{\gamma B_{\epsilon , t} - \frac{\gamma ^2}{2} \mathbb {E}[B_{\epsilon , t}^2]} e^{(d+\gamma ^2)t}Z_{\gamma , \epsilon }(dt)\right) ^{p-1}\right] \end{aligned}$$

for $T = -\log K \epsilon$, and $Z_{\gamma , \epsilon }(dt)$ is defined in the obvious way. If $-\log r \le s \le t \le T$,

$$\begin{aligned} \mathbb {E}\left[ B_{\epsilon , s} B_{\epsilon , t}\right]&= \int \int \nu (u) \nu (v) du dv\mathbb {E}\left[ B_{-\log |e^{-s}e_1 + \epsilon v|}B_{-\log |e^{-t}e_1 + \epsilon v|}\right] \\&\in [s - \log |1 + \epsilon e^{s}|, s - \log |1 - \epsilon e^{s}|] \end{aligned}$$

where $\epsilon e^{s} \le K^{-1}$. This means that

46 $$\begin{aligned} \left| \mathbb {E}\left[ B_s B_t\right] - \mathbb {E}\left[ B_{\epsilon , s} B_{\epsilon , t}\right] \right| \le \frac{2}{K} \qquad \forall s, t \in [-\log r, T] \end{aligned}$$

for $K>1$ sufficiently large, and by Corollary 2 we obtain

$$\begin{aligned} e^{-\frac{\gamma ^2}{K}|(p-1)(p-2)|} \le \frac{\mathbb {E}\left[ \left( \int _{-\log r}^T e^{\gamma B_t - \frac{\gamma ^2}{2} \mathbb {E}[B_t^2]} e^{(d+\gamma ^2)t}Z_{\gamma , \epsilon }(dt)\right) ^{p-1}\right] }{\mathbb {E}\left[ \left( \int _{-\log r}^T e^{\gamma B_{\epsilon , t} - \frac{\gamma ^2}{2} \mathbb {E}[B_{\epsilon , t}^2]} e^{(d+\gamma ^2)t}Z_{\gamma , \epsilon }(dt)\right) ^{p-1}\right] } \le e^{\frac{\gamma ^2}{K}|(p-1)(p-2)|}. \end{aligned}$$

Therefore it suffices to consider

$$\begin{aligned} \mathbb {E}\left[ \left( \int _{-\log r}^T e^{\gamma B_t - \frac{\gamma ^2}{2} \mathbb {E}[B_t^2]} e^{(d+\gamma ^2)t}Z_{\gamma , \epsilon }(dt)\right) ^{p-1}\right] =\mathbb {E}\left[ \left( \int _{-\log r}^T e^{\gamma (B_t - \mu t)} Z_{\gamma , \epsilon }(dt)\right) ^{p-1}\right] \end{aligned}$$

where $\mu = \frac{\gamma }{2}(p-1)$. By Theorem 4, we can decompose the path of $(B_t - \mu t)_{t \ge 0}$ and rewrite our expectation as

47 $$\begin{aligned}&\mathbb {E}\left[ \mathbb {E}\left[ \left( \int _{-\log r}^T e^{\gamma (B_t - \mu t)} Z_{\gamma , \epsilon }(dt)\right) ^{p-1} \Bigg | \max _{t \ge 0} (B_t - \mu t)\right] \right] \nonumber \\&= \mathbb {E}\left[ \mathbb {E}\left[ e^{\gamma (p-1)\mathcal {M}} \left( \int _{-\log r}^{T} e^{\gamma (B_t^\mu -\mathcal {M}) 1_{\{t \le \tau _\mathcal {M}\}} - \gamma \mathcal {B}_{t - \tau _\mathcal {M}}^\mu 1_{\{t \ge \tau _\mathcal {M}\}}} Z_{\gamma , \epsilon } (dt)\right) ^{p-1} \Bigg | \mathcal {M}\right] \right] \nonumber \\&= 2\mu \int _0^{\infty } dm \mathbb {E}\left[ \left( \int _{-\log r}^{T\wedge \tau _m} e^{\gamma (B_t^\mu -m)} Z_{\gamma , \epsilon } (dt) + \int _0^{(T - \tau _m)_+} e^{-\gamma \mathcal {B}_t^\mu } Z_{\gamma , \epsilon } \circ \phi _{\tau _m}(dt)\right) ^{p-1} \right] \end{aligned}$$

where $\phi _c: x \mapsto x+c$ is the right shift and $\tau _m:= \inf \{t > 0: B_t^\mu = m\}$. Using Lemma 4, the integrand may also be written as

48 $$\begin{aligned} \mathbb {E}\left[ \left( \int _{-L_{m} - \log r}^{(T-L_{m})_+} e^{-\gamma \mathcal {B}_t^\mu } Z_{\gamma , \epsilon }\circ \phi _{L_{m}} (dt)\right) ^{p-1}\right] \end{aligned}$$

with $$L_{m} := \sup \{t > 0: \mathcal {B}_{-t}^\mu = m\}$$.

*Step 2: a uniform upper bound.* We claim that the integrand in (47) is uniformly bounded in *m* and $\epsilon$ (for any fixed $K>1$ and $r \in (0, \frac{1}{4})$). It is more convenient to deal with the representation (48), and we will show that

49 $$\begin{aligned} \mathbb {E}\left[ \left( \int _{-\infty }^\infty e^{-\gamma \mathcal {B}_t^\mu } Z_{\gamma , \epsilon }\circ \phi _{L_{m}} (dt)\right) ^{p-1}\right] \le C_p \end{aligned}$$

for some constants $C_p \in (0, \infty )$ independent of *m* and $\epsilon$. For future reference, we will actually show a slightly strengthened version of (49), namely: for any $c \ge 0$,

50 $$\begin{aligned} \mathbb {E}\left[ \left( \int _{|t| \ge c} e^{-\gamma \mathcal {B}_t^\mu } Z_{\gamma , \epsilon }\circ \phi _{L_{m}} (dt)\right) ^{p-1}\right] \le C_p(c) \end{aligned}$$

for some $C_p(c) \in (0, \infty )$ independent of *m* and $\epsilon$, such that $C_p(c)$ is monotonically decreasing to 0 as $c \rightarrow \infty$. There are two cases to consider:

- $p-1 \ge 1$, i.e. the map $x \mapsto x^{p-1}$ is convex. First observe the distributional equality $Z_{\gamma , \epsilon } \circ \phi _c(dt) \overset{d}{=} Z_{\gamma , \epsilon e^c}(dt)$ for any $c \ge 0$ (possibly random but independent of ${\widehat{X}}$ (see (32) and (33) in the proof of Lemma 5 for the details). Therefore, our integrand may be bounded by first rewriting it as
  $$\begin{aligned} \mathbb {E}\left[ \mathbb {E}\left[ \left( \int _{|t| \ge c} e^{-\gamma \mathcal {B}_t^\mu } Z_{\gamma , \epsilon \exp (L_{m})} (dt)\right) ^{p-1} \Bigg | (\mathcal {B}_t^\mu )_{t \in \mathbb {R}} \right] \right] \end{aligned}$$
  and then performing Gaussian comparison in the conditional expectation. Since
  $$\begin{aligned} \mathbb {E}\left[ {\widehat{X}}_{\epsilon }(x) {\widehat{X}}_\epsilon (y)\right] \le \mathbb {E}\left[ {\widehat{X}}(x) {\widehat{X}}(y)\right] + C \end{aligned}$$
  for some $C > 0$ independent of $\epsilon > 0$, Corollary 2 implies that
  $$\begin{aligned}&\mathbb {E}\left[ \left( \int _{|t| \ge c} e^{-\gamma \mathcal {B}_t^\mu } Z_{\gamma , \epsilon \exp (L_{m})} (dt)\right) ^{p-1} \right] \\&\qquad \le e^{\frac{\gamma ^2}{2}p(p-1) C}\mathbb {E}\left[ \left( \int _{|t| \ge c} e^{-\gamma \mathcal {B}_t^\mu } Z_{\gamma } (dt)\right) ^{p-1} \right] . \end{aligned}$$
  When $c = 0$, the expectation appearing in the last bound is the probabilistic representation of ${\overline{C}}_{\gamma , d}$ (see Lemma 6) and is thus finite. If we take the above bound as the definition of $C_p(c)$, then $C_p(c)$ satisfies all the desired properties (uniformity in $m, \epsilon$ and monotone convergence to 0 as $c \rightarrow \infty$) in our claim.
- $p-1 \in (0, 1)$, i.e. the map $x \mapsto x^{p-1}$ is concave. By Jensen’s inequality and subadditivity, we have
  $$\begin{aligned} \mathbb {E}\left[ \left( \int _{|t| \ge c} e^{-\gamma \mathcal {B}_t^\mu } Z_{\gamma , \epsilon \exp (L_{m})} (dt)\right) ^{p-1} \right]&\le \mathbb {E}\left[ \int _{|t| \ge c} e^{-\gamma \mathcal {B}_t^\mu } Z_{\gamma , \epsilon \exp (L_{m})} (dt)\right] ^{p-1}\\&\le 4\pi \mathbb {E}\left[ \int _{c}^\infty e^{-\gamma \mathcal {B}_t^\mu } dt\right] ^{p-1}. \end{aligned}$$
  Again we can take the above bound as the definition of $C_p(c)$, which obviously satisfies all the desired properties except the claim that $C_p(0) < \infty$ which we verify now. By Lemma 3, $(\mathcal {B}_t^\mu )_{t \ge 0}$ stochastically dominates a $\mathrm {BES}_0(3)$-process, which we denote by $(\beta _t)_{t \ge 0}$. In particular, using the fact that $\beta _t \overset{d}{=} \sqrt{t}\beta _1$ for any fixed $t > 0$, we have
  $$\begin{aligned} \mathbb {E}\left[ \int _{0}^\infty e^{-\gamma \mathcal {B}_t^\mu } dt\right]&= \int _{0}^\infty \mathbb {E}\left[ e^{-\gamma \mathcal {B}_t^\mu }\right] dt\\&\le \int _{0}^\infty \mathbb {E}\left[ e^{-\gamma \beta _t}\right] dt\\&= \mathbb {E}\left[ \int _{0}^\infty e^{-\gamma \sqrt{t}\beta _1} dt\right] \overset{u = \beta _1^2 t}{=} \mathbb {E}\left[ \beta _1^{-2}\right] \int _0^\infty e^{-\gamma \sqrt{u}} du < \infty \end{aligned}$$
  and so we are done.

To analyse the integrand further, we fix some small $\delta \in (0, 1/4)$ and proceed by splitting the *m*-integral into two regions.

*Step 3: main contribution from* $m \le \mu (1-2\delta )T$. Let us fix some $m_0 > 0$ and ignore all the contributions to the integral from $m < m_0$. We recall from Lemma 4 that the law of $L_{m}$ is the same as that of $\tau _m := \inf \{t > 0: B_t^\mu = m\}$ and so the event $A_\delta := \{ L_{m} \le (1-\delta )T\}$ satisfies

$$\begin{aligned} \mathbb {P}(A_\delta ) \ge \mathbb {P}\left( \max _{t \le T} B_t^\mu \ge \mu (1-\delta )T\right) \xrightarrow {T \rightarrow \infty } 1 \end{aligned}$$

for any $m \le \mu (1-\delta )T$. Then

51 $$\begin{aligned}&\mathbb {E}\left[ \left( \int _{-L_{m} - \log r}^{(T-L_{m})_+} e^{-\gamma \mathcal {B}_t^\mu } Z_{\gamma , \epsilon }\circ \phi _{L_{m}} (dt)\right) ^{p-1}\right] \nonumber \\&\qquad = \mathbb {E}\left[ \left( \int _{-L_{m} - \log r}^{(T-L_{m})_+} e^{-\gamma \mathcal {B}_t^\mu } Z_{\gamma , \epsilon \exp (L_{m})} (dt)\right) ^{p-1}\right] \nonumber \\&\qquad \ge \mathbb {E}\left[ \left( \int _{-L_{m_0} - \log r}^{\delta T} e^{-\gamma \mathcal {B}_t^\mu } Z_{\gamma , \epsilon \exp (L_{m})} (dt)\right) ^{p-1} 1_{A_\delta }\right] \nonumber \\&\qquad \ge \mathbb {E}\left[ \left( \int _{-L_{m_0} - \log r}^{M} e^{-\gamma \mathcal {B}_t^\mu } Z_{\gamma , \epsilon \exp (L_{m})} (dt)\right) ^{p-1} \right] \nonumber \\&\qquad \qquad - \mathbb {E}\left[ \left( \int _{-L_{m_0} - \log r}^{M} e^{-\gamma \mathcal {B}_t^\mu } Z_{\gamma , \epsilon \exp (L_{m})} (dt)\right) ^{p-1} 1_{A_\delta ^c}\right] \end{aligned}$$

where the last inequality just follows from the truncation of the integral at a deterministic (but arbitrary) level $M \le \delta T$ (which is allowed since $T = -\log K \epsilon$ is arbitrarily large as $\epsilon \rightarrow 0^+$).

Since $L_m$ is almost surely finite and only depends on $(\mathcal {B}_t^\mu )_{t \in \mathbb {R}}$ (and in particular independent of ${\widehat{X}}$), we have $Z_{\gamma , \epsilon \exp (L_m)} \xrightarrow {\epsilon \rightarrow 0^+} Z_{\gamma }$ in probability in the weak$^*$-topology of measures on any compact intervals of $\mathbb {R}$. Combining this with the fact that the $(p-1)$-th power of the total mass of regularised GMC measures are uniformly integrable and that their expectation converges to the $(p-1)$-th moment of limiting GMC measure (see Lemma 7), we see that

$$\begin{aligned}&\mathbb {E}\left[ \left( \int _{-L_{m_0} - \log r}^{M} e^{-\gamma \mathcal {B}_t^\mu } Z_{\gamma , \epsilon \exp (L_{m})} (dt)\right) ^{p-1} \right] \\&\qquad \xrightarrow {\epsilon \rightarrow 0^+} \mathbb {E}\left[ \left( \int _{-L_{m_0} - \log r}^{M} e^{-\gamma \mathcal {B}_t^\mu } Z_{\gamma } (dt)\right) ^{p-1} \right] . \end{aligned}$$

Similarly, since $\mathbb {P}(A_\delta ^c) \xrightarrow {\epsilon \rightarrow 0^+} 0$, the uniform integrability of the $(p-1)$-th moments of the regularised GMCs implies

$$\begin{aligned} \mathbb {E}\left[ \left( \int _{-L_{m_0} - \log r}^{M} e^{-\gamma \mathcal {B}_t^\mu } Z_{\gamma , \epsilon \exp (L_{m})} (dt)\right) ^{p-1} 1_{A_\delta ^c}\right] \xrightarrow {\epsilon \rightarrow 0^+} 0. \end{aligned}$$

Since $M> 0$ is arbitrary, we obtain from (51) the lower bound

52 $$\begin{aligned}&\liminf _{\epsilon \rightarrow 0^+} \mathbb {E}\left[ \left( \int _{-L_{m} - \log r}^{(T-L_{m})_+} e^{-\gamma \mathcal {B}_t^\mu } Z_{\gamma , \epsilon }\circ \phi _{L_{m}} (dt)\right) ^{p-1}\right] \nonumber \\&\qquad \qquad \ge \mathbb {E}\left[ \left( \int _{-L_{m_0} - \log r}^{\infty } e^{-\gamma \mathcal {B}_t^\mu } Z_{\gamma } (dt)\right) ^{p-1} \right] . \end{aligned}$$

As for the upper bound, consider

53 $$\begin{aligned}&\mathbb {E}\left[ \left( \int _{-L_{m} - \log r}^{(T-L_{m})_+} e^{-\gamma \mathcal {B}_t^\mu } Z_{\gamma , \epsilon }\circ \phi _{L_{m}} (dt)\right) ^{p-1}\right] \nonumber \\&\qquad \qquad \le \mathbb {E}\left[ \left( \int _{-\infty }^\infty e^{-\gamma \mathcal {B}_t^\mu } Z_{\gamma , \epsilon }\circ \phi _{L_{m}} (dt)\right) ^{p-1} \right] \nonumber \\&\qquad \qquad = \mathbb {E}\left[ \left( \int _{-\infty }^\infty e^{-\gamma \mathcal {B}_t^\mu } Z_{\gamma , \epsilon \exp (L_{m})} (dt)\right) ^{p-1} \right] \end{aligned}$$

in the following two cases:

- $p-1 \ge 1$. We can upper bound the $(p-1)$-th root of (53) by
  $$\begin{aligned}&\mathbb {E}\left[ \left( \int _{|t| \le M} e^{-\gamma \mathcal {B}_t^\mu } Z_{\gamma , \epsilon \exp (L_{m})} (dt)\right) ^{p-1} \right] ^{\frac{1}{p-1}}\\&\qquad + \mathbb {E}\left[ \left( \int _{|t| > M} e^{-\gamma \mathcal {B}_t^\mu } Z_{\gamma , \epsilon \exp (L_{m})} (dt)\right) ^{p-1} \right] ^{\frac{1}{p-1}} \end{aligned}$$
  for some fixed $M > 0$.
- $p \in (0, 1)$. We can upper bound (53) by
  $$\begin{aligned}&\mathbb {E}\left[ \left( \int _{|t| \le M} e^{-\gamma \mathcal {B}_t^\mu } Z_{\gamma , \epsilon \exp (L_{m})} (dt)\right) ^{p-1} \right] \\&\qquad + \mathbb {E}\left[ \left( \int _{|t| > M} e^{-\gamma \mathcal {B}_t^\mu } Z_{\gamma , \epsilon \exp (L_{m})} (dt)\right) ^{p-1} \right] \end{aligned}$$
  for some fixed $M > 0$.

Under either scenario, the first term provides the main contribution, and the same argument from the lower bound leads to

$$\begin{aligned}&\mathbb {E}\left[ \left( \int _{|t| \le M} e^{-\gamma \mathcal {B}_t^\mu } Z_{\gamma , \epsilon \exp (L_{m})} (dt)\right) ^{p-1} \right] \\&\xrightarrow {\epsilon \rightarrow 0^+} \mathbb {E}\left[ \left( \int _{|t| \le M} e^{-\gamma \mathcal {B}_t^\mu } Z_{\gamma } (dt)\right) ^{p-1} \right] \le \mathbb {E}\left[ \left( \int _{-\infty }^{\infty } e^{-\gamma \mathcal {B}_t^\mu } Z_{\gamma } (dt)\right) ^{p-1} \right] . \end{aligned}$$

On the other hand, the uniform bound from Step 2 gives us a control on the residual term:

$$\begin{aligned} \mathbb {E}\left[ \left( \int _{|t| > M} e^{-\gamma \mathcal {B}_t^\mu } Z_{\gamma , \epsilon \exp (L_{m})} (dt)\right) ^{p-1} \right] ^{\frac{1}{p-1}} \le C_p(M)^{\frac{1}{p-1}} \end{aligned}$$

where $C_p(\cdot ) \in (0, \infty )$ is defined in (50) with the property that $C_p(M) \rightarrow 0$ as $M \rightarrow \infty$. Since $M > 0$ is arbitrary, we arrive at the upper bound

54 $$\begin{aligned}&\limsup _{\epsilon \rightarrow 0} \mathbb {E}\left[ \left( \int _{-L_{m} - \log r}^{(T-L_{m})_+} e^{-\gamma \mathcal {B}_t^\mu } Z_{\gamma , \epsilon }\circ \phi _{L_{m}} (dt)\right) ^{p-1}\right] \nonumber \\&\qquad \qquad \le \mathbb {E}\left[ \left( \int _{-\infty }^{\infty } e^{-\gamma \mathcal {B}_t^\mu } Z_{\gamma } (dt)\right) ^{p-1} \right] . \end{aligned}$$

Combining (52) and (54), we have

$$\begin{aligned}&\mu (1-2\delta ) \mathbb {E}\left[ \left( \int _{-L_{m_0} - \log r}^{\infty } e^{-\gamma \mathcal {B}_t^\mu } Z_{\gamma } (dt)\right) ^{p-1} \right] \\&\qquad \le \liminf _{\epsilon \rightarrow 0^+} \frac{1}{T} \int _0^{\mu (1-2\delta )T} \mathbb {E}\left[ \left( \int _{-L_{m} - \log r}^{(T-L_{m})_+} e^{-\gamma \mathcal {B}_t^\mu } Z_{\gamma , \epsilon }\circ \phi _{L_{m}} (dt)\right) ^{p-1}\right] \\&\qquad \le \limsup _{\epsilon \rightarrow 0^+} \frac{1}{T} \int _0^{\mu (1-2\delta )T} \mathbb {E}\left[ \left( \int _{-L_{m} - \log r}^{(T-L_{m})_+} e^{-\gamma \mathcal {B}_t^\mu } Z_{\gamma , \epsilon }\circ \phi _{L_{m}} (dt)\right) ^{p-1}\right] \\&\qquad \le \mu (1-2\delta )\mathbb {E}\left[ \left( \int _{-\infty }^{\infty } e^{-\gamma \mathcal {B}_t^\mu } Z_{\gamma } (dt)\right) ^{p-1} \right] . \end{aligned}$$

The lower bound then matches the upper bound as we send $m_0$ to infinity, and this coincides with $\mu (1-2\delta ) {\overline{C}}_{\gamma , d}$ by (35) in Lemma 6.

*Step 4: remaining contribution.* We can use the crude bound for the integrand from Step 2 so that

$$\begin{aligned} \frac{1}{T}\int _{\mu (1-2\delta ) T}^{\mu (1+4\delta )T} \mathbb {E}\left[ \left( \int _{-\infty }^\infty e^{-\gamma \mathcal {B}_t^\mu } Z_{\gamma , \epsilon }\circ \phi _{L_{m}} (dt)\right) ^{p-1}\right] \le 6\delta \mu C_{p} \end{aligned}$$

where $C_p$ is defined in (49). It suffices to control the remaining contribution from $m \ge \mu (1+4\delta )T$. We consider a different event here, namely

$$\begin{aligned} {\widetilde{A}}_\delta = \{\tau _{(1-\delta )m} > (1+\delta )T\} = \left\{ \max _{t \le (1+\delta )T} B_t^\mu < (1-\delta )m\right\} . \end{aligned}$$

On this event, it is actually more convenient to work with the original representation of the integrand, i.e. (47). Since $\tau _m> \tau _{(1-\delta )m} > T$ and $\max _{t \le T} B_t^\mu \le (1-\delta )m$, we see that

$$\begin{aligned}&\mathbb {E}\left[ \left( \int _{-\log r}^{T\wedge \tau _m} e^{\gamma (B_t^\mu -m)} Z_{\gamma , \epsilon } (dt) + \int _0^{(T - \tau _m)_+} e^{-\gamma \mathcal {B}_t^\mu } Z_{\gamma , \epsilon } \circ \phi _{\tau _m}(dt)\right) ^{p-1} 1_{{\widetilde{A}}_\delta }\right] \\&\quad \le e^{-(p-1)\delta \gamma m} \mathbb {E}\left[ \left( \int _{-\log r}^{\tau _{(1-\delta )m}} e^{\gamma (B_t^\mu -(1-\delta ) m)} Z_{\gamma , \epsilon } (dt) \right) ^{p-1} 1_{{\widetilde{A}}_\delta }\right] \\&\quad \le e^{-(p-1)\delta \gamma m} \mathbb {E}\left[ \left( \int _{-\infty }^0 e^{-\gamma \mathcal {B}_t^\mu } Z_{\gamma , \epsilon } \circ \phi _{L_{(1-\delta )m}} (dt) \right) ^{p-1} 1_{{\widetilde{A}}_\delta }\right] \\&\quad \le C_p e^{-(p-1)\delta \gamma m} \end{aligned}$$

where the second inequality follows from time reversal (Lemma 4), and the constant $C_p < \infty$ in the last inequality is again the bound in (49).

On the complementary event, we have

$$\begin{aligned}&\mathbb {E}\left[ \left( \int _{-\log r}^{T\wedge \tau _m} e^{\gamma (B_t^\mu -m)} Z_{\gamma , \epsilon } (dt) + \int _0^{(T - \tau _m)_+} e^{-\gamma \mathcal {B}_t^\mu } Z_{\gamma , \epsilon } \circ \phi _{\tau _m}(dt)\right) ^{p-1} 1_{{\widetilde{A}}_\delta ^c}\right] \\&\le 2^{p-1} \Bigg \{\mathbb {E}\left[ \left( \int _{-L_m}^{0} e^{-\gamma \mathcal {B}_t^\mu } Z_{\gamma , \epsilon } \circ \phi _{L_m}(dt)\right) ^{p-1} 1_{{\widetilde{A}}_\delta ^c}\right] \\&\qquad \qquad + \mathbb {E}\left[ \left( \int _{0}^{(T - L_m)_+} e^{-\gamma \mathcal {B}_t^\mu } Z_{\gamma , \epsilon } \circ \phi _{L_m}(dt)\right) ^{p-1} 1_{{\widetilde{A}}_\delta ^c}\right] \Bigg \}\\&=: 2^{p-1}(I + II) \end{aligned}$$

where we abused the notation in the first inequality and considered the dual interpretation ${\widetilde{A}}_\delta ^c = \left\{ L_{(1-\delta )m} \le (1+\delta )T\right\}$ when we applied time reversal. Using the fact that $\mathbb {P}(\mathcal {N}(0,1) > t) \le e^{-t^2/2}$ and $m \ge \mu (1+4\delta ) T$, we have

$$\begin{aligned} \mathbb {P}\left( {\widetilde{A}}_\delta ^c\right)&= \mathbb {P}\left( \tau _{(1-\delta )m} \le (1+\delta )T\right) \\&\le \mathbb {P}\left( \max _{t \le (1+\delta )T} B_t \ge (1-\delta )m - \mu T\right) \\&= 2\mathbb {P}\left( B_{(1+\delta )T}\ge (1-\delta )m - \mu T\right) \\&\le 2 \exp \left( -\frac{1}{2} \left( \frac{(1-\delta )m - \mu T}{\sqrt{(1+\delta )T}}\right) ^2\right) \le 2 \exp \left( -\frac{\mu }{2} \frac{3\delta - 4\delta ^2}{1+\delta } \left[ (1-\delta )m - \mu T \right] \right) \end{aligned}$$

so that

$$\begin{aligned} II&\le \mathbb {E}\left[ \left( \int _{0}^{(T - L_m)_+} e^{-\gamma \mathcal {B}_t^\mu } Z_{\gamma , \epsilon } \circ \phi _{L_m}(dt)\right) ^{p-1} \right] \mathbb {P}\left( {\widetilde{A}}_\delta ^c\right) \\&\le 2 C_p \exp \left( -\frac{\mu }{2} \frac{3\delta - 4\delta ^2}{1+\delta } \left[ (1-\delta )m - \mu T \right] \right) \end{aligned}$$

by independence (as ${\widetilde{A}}_\delta ^c$ only depends on $(\mathcal {B}_t^\mu )_{t \le 0}$). As for the first term, pick $\alpha > 1$ small enough so that $1 \le \alpha (p-1) < p$, and we obtain

$$\begin{aligned} I&\le \mathbb {E}\left[ \left( \int _{-L_m}^{0} e^{-\gamma \mathcal {B}_t^\mu } Z_{\gamma , \epsilon } \circ \phi _{L_m}(dt)\right) ^{\alpha (p-1)}\right] ^{1/\alpha } \mathbb {P}\left( {{\widetilde{A}}_\delta ^c}\right) ^{1 - \frac{1}{\alpha }}\\&\le C\mathbb {E}\left[ \left( \int _{-L_m}^{0} e^{-\gamma \mathcal {B}_t^\mu } Z_{\gamma }(dt)\right) ^{\alpha (p-1)}\right] ^{1/\alpha } \mathbb {P}\left( {{\widetilde{A}}_\delta ^c}\right) ^{1 - \frac{1}{\alpha }}\\&\le C'\mathbb {E}\left[ \left( \int _{-\infty }^0 e^{-\gamma \mathcal {B}_t^\mu } Z_{\gamma }(dt)\right) ^{\alpha (p-1)}\right] ^{1/\alpha } \exp \left( -\frac{\mu }{2} \frac{3\delta - 4\delta ^2}{1+\delta } (1-\alpha ^{-1})\left[ (1-\delta )m - \mu T \right] \right) \end{aligned}$$

by Hölder’s inequality and Gaussian comparison (to replace $Z_{\gamma , \epsilon } \circ \phi _{L_m}$ with $Z_{\gamma }$ up to a multiplicative error $C>0$). Our task now is to show that

55 $$\begin{aligned} \mathbb {E}\left[ \left( \int _{-\infty }^0 e^{-\gamma \mathcal {B}_t^\mu } Z_{\gamma }(dt)\right) ^{\alpha (p-1)}\right] ^{\frac{1}{\alpha (p-1)}} < \infty . \end{aligned}$$

For this, consider

56 $$\begin{aligned}&\mathbb {E}\left[ \left( \int _{-\infty }^0 e^{-\gamma \mathcal {B}_t^\mu } Z_{\gamma }(dt)\right) ^{\alpha (p-1)}\right] ^{\frac{1}{\alpha (p-1)}}\nonumber \\&\qquad \le \sum _{n \ge 1} e^{-\gamma n}\mathbb {E}\left[ \left( \int _{L_{n-1}}^{L_{n}} e^{-\gamma (\mathcal {B}_{-t}^\mu - \mathcal {B}_{-L_{n-1}}^\mu )} Z_{\gamma }(dt)\right) ^{\alpha (p-1)}\right] ^{\frac{1}{\alpha (p-1)}}\nonumber \\&\qquad \le \left( \sum _{n \ge 1} e^{-\gamma n}\right) \mathbb {E}\left[ \left( \int _{0}^{L_1} e^{-\gamma \mathcal {B}_{-t}^\mu } Z_{\gamma }(dt)\right) ^{\alpha (p-1)}\right] ^{\frac{1}{\alpha (p-1)}} \end{aligned}$$

where the last equality follows from Corollary 3. To analyse the expectation, we first split it into

57 $$\begin{aligned} \mathbb {E}\left[ \left( \int _{0}^{L_1} e^{-\gamma \mathcal {B}_{-t}^\mu } Z_{\gamma }(dt)\right) ^{\alpha (p-1)}\right] ^{\frac{1}{\alpha (p-1)}}&\le \sum _{n \ge 0} \mathbb {E}\left[ \left( \int _{n}^{n+1} Z_{\gamma }(dt) \right) ^{\alpha (p-1)} 1_{\{L_1 \ge n\}}\right] ^{\frac{1}{\alpha (p-1)}} \end{aligned}$$

where

$$\begin{aligned} \mathbb {E}\left[ \left( \int _{n}^{n+1} Z_{\gamma }(dt) \right) ^{\alpha (p-1)}\right] ^{\frac{1}{\alpha (p-1)}} = \mathbb {E}\left[ \left( \int _{0}^{1} Z_{\gamma }(dt) \right) ^{\alpha (p-1)}\right] ^{\frac{1}{\alpha (p-1)}} \end{aligned}$$

by translation invariance (Lemma 5), and this quantity is finite due to the existence of GMC moments. Meanwhile, from Theorem 3, we have

$$\begin{aligned} \mathbb {P}\left( L_1 \ge n\right) = \mathbb {P}\left( \inf _{s \ge n} \mathcal {B}_s^\mu \le 1 \right)&= \mathbb {P}\left( \max _{s \le n} B_s^\mu \le 1\right) \\&\le \mathbb {P}\left( B_n + \mu n \le 1\right) \le \exp \left( -\frac{1}{2n} (1-\mu n)^2\right) . \end{aligned}$$

Applying Hölder’s inequality, (57) can be further bounded by

$$\begin{aligned}&\sum _{n \ge 0} \mathbb {E}\left[ \left( \int _{n}^{n+1} Z_{\gamma }(dt) \right) ^{\alpha q(p-1)}\right] ^{\frac{1}{\alpha q(p-1)}} \mathbb {P}\left( L_1 \ge n\right) ^{1 - \frac{1}{q}} \end{aligned}$$

which is summable provided that $q > 1$ is chosen such that $\alpha q(p-1) < p$ so that

$$\begin{aligned} \mathbb {E}\left[ \left( \int _{n}^{n+1} Z_{\gamma }(dt) \right) ^{\alpha q(p-1)}\right] =\mathbb {E}\left[ \left( \int _{0}^{1} Z_{\gamma }(dt) \right) ^{\alpha q(p-1)}\right] < \infty \end{aligned}$$

by the existence of GMC moments again. Therefore, (56) and hence (55) are all finite, as claimed.

Gathering all terms, we see that there exists some constant $C>0$ independent of *T* (or equivalently $\epsilon$) and $m \ge \mu (1+4\delta )T$ (but possibly depends on other parameters) such that

$$\begin{aligned}&\mathbb {E}\left[ \left( \int _{-\log r}^{T\wedge \tau _m} e^{\gamma (B_t^\mu -m)} Z_{\gamma , \epsilon } (dt) + \int _0^{(T - \tau _m)_+} e^{-\gamma \mathcal {B}_t^\mu } Z_{\gamma , \epsilon } \circ \phi _{\tau _m}(dt)\right) ^{p-1} \right] \\&\qquad \le C \exp \left( -C^{-1} \left( (1-\delta )m - \mu T\right) \right) \end{aligned}$$

and hence

$$\begin{aligned}&\int _{m \ge \mu (1+4\delta )T} dm \mathbb {E}\left[ \left( \int _{-\log r}^{T\wedge \tau _m} e^{\gamma (B_t^\mu -m)} Z_{\gamma , \epsilon } (dt) + \int _0^{(T - \tau _m)_+} e^{-\gamma \mathcal {B}_t^\mu } Z_{\gamma , \epsilon } \circ \phi _{\tau _m}(dt)\right) ^{p-1} \right] \\&\qquad \le \frac{1}{1-\delta } e^{-\mu \left[ (1-\delta )(1+4\delta ) - 1\right] T} \le 2 \end{aligned}$$

for $\delta > 0$ sufficiently small.

*Step 5: conclusion.* Summarising all the work we have done so far, we have

$$\begin{aligned}&\mathbb {E}\left[ \left( \int _{K\epsilon \le |x| \le r} e^{\gamma {\overline{X}}_{\epsilon }(x)- \frac{\gamma ^2}{2} \mathbb {E}[{\overline{X}}_{\epsilon }(x)^2]}\frac{dx}{|x|^{\gamma ^2}} \right) ^{p-1}\right] \\&= \left( 1 + \mathcal {O}(K^{-1})\right) 2\mu \int _0^{\infty } dm \mathbb {E}\Bigg [\Bigg ( \int _{-\log r}^{T\wedge \tau _m} e^{\gamma (B_t^\mu -m)} Z_{\gamma , \epsilon } (dt) \\&\qquad \qquad \qquad \qquad \qquad \qquad \qquad + \int _0^{(T - \tau _m)_+} e^{-\gamma \mathcal {B}_t^\mu } Z_{\gamma , \epsilon } \circ \phi _{\tau _m}(dt)\Bigg )^{p-1} \Bigg ]\\&= \left( 1 + \mathcal {O}(K^{-1})\right) 2\mu \left[ \mu {\overline{C}}_{\gamma , d} T + \mathcal {O}(1+\delta T)\right] \end{aligned}$$

where $T = -\log K\epsilon$. Dividing both sides by $-\log \epsilon$ and taking the limit $\epsilon \rightarrow 0^+$, the RHS becomes $\left( 1 + \mathcal {O}(K^{-1})\right) 2\mu ^2 {\overline{C}}_{\gamma , d} + \mathcal {O}(\delta )$. Since $K > 1$ and $\delta > 0$ are arbitrary, we send *K* to infinity and $\delta$ to zero and obtain the desired result.

##### Remark 5

To treat the case where $g \ge 0$ is continuous on ${\overline{D}}$, one would first obtain the analogue of Lemma 11 by following the same argument, i.e.

$$\begin{aligned}&\mathbb {E}\left[ \left( \int _D g(x)M_{\gamma , \epsilon }(dx)\right) ^p\right] \\&= \int _D g(u) du\mathbb {E}\left[ \left( \int _{D \cap \{K\epsilon \le |x-u| \le r\}} g(x) \frac{e^{\gamma ^2 f_\epsilon (x, u)}}{|x-u|^{\gamma ^2}} M_{\gamma , \epsilon }(dx)\right) ^{p-1}\right] + \mathcal {O}(1). \end{aligned}$$

When *r* is small, $g(x) \approx g(u)$ for $|x-u| \le r$, and so the integral above can be further approximated by

$$\begin{aligned}&= \int _D g(u)^p du\mathbb {E}\left[ \left( \int _{D \cap \{K\epsilon \le |x-u| \le r\}} \frac{e^{\gamma ^2 f_\epsilon (x, u)}}{|x-u|^{\gamma ^2}} M_{\gamma , \epsilon }(dx)\right) ^{p-1}\right] \end{aligned}$$

and no further changes are needed for the rest of the analysis. This explains the appearance of $g(u)^p$ in the leading order coefficient in Theorem 1.

### General approximation: proof of Corollary 1

Not surprisingly, the generalisation of Theorem 1 to Corollary 1 relies on Gaussian comparison. To achieve this, we need the following technical estimate.

#### Lemma 14

Let $\epsilon , \delta \in (0, 1)$ and $r = \epsilon ^{1-\delta }$. Then for $\gamma \in (0, \sqrt{2d})$, $p = p_c = 2d/\gamma ^2$, we have

$$\begin{aligned} \mathbb {E}\left[ M_{\gamma , \epsilon }(B(x_0, r)\cap D)^2 M_{\gamma , \epsilon }(D)^{p-2}\right] \le C r^d \log \frac{r}{\epsilon } \end{aligned}$$

where the constant $C>0$ is uniform in $\epsilon , \delta$, $x_0 \in D$, and can be further chosen to be uniformly for all Gaussian fields $X_\epsilon$ with covariance of the form

$$\begin{aligned} \mathbb {E}\left[ X_\epsilon (x) X_\epsilon (y)\right] = -\log \left( |x- y| \vee \epsilon \right) + f_\epsilon (x, y) \qquad \forall x, y \in D \end{aligned}$$

where $\sup _{\epsilon > 0} \sup _{x, y \in D} |f_\epsilon (x, y)| \le K$ for some $K > 0$.

#### Proof

To simplify the notations let us verify the inequality for $x_0 = 0$, assuming that the domain contains the origin and that $\mathrm {diam}(D) := \sup \{|x-y| : x, y \in D\} \le 1$. The constant $C>0$ below will vary from line to line but its uniformity in $\epsilon , \delta$ and $x_0 \in D$ should be evident from the proof.

To begin with, consider

58 $$\begin{aligned}&\mathbb {E}\left[ M_{\gamma , \epsilon }(B(x_0, r))^2 M_{\gamma , \epsilon }(D)^{p-2}\right] \nonumber \\&\quad = \int _{|u| \le r} \int _{|v| \le r} du dv\mathbb {E}\left[ e^{\gamma (X_\epsilon (u) + X_\epsilon (v)) - \frac{\gamma ^2}{2} \mathbb {E}[X_\epsilon (u)^2 + X_\epsilon (v)^2]}M_{\gamma , \epsilon }(D)^{p-2}\right] \nonumber \\&\quad \le C \int _{|u|\le r} du \int _{|v| \le r} \frac{dv}{(|u-v| \vee \epsilon )^{\gamma ^2}} \mathbb {E}\left[ \left( \int _D \frac{M_{\gamma , \epsilon }(dx)}{(|x-u|\vee \epsilon )^{\gamma ^2}(|x-v|\vee \epsilon )^{\gamma ^2}} \right) ^{p-2}\right] . \end{aligned}$$

Before we continue with our bounds, let us mention that the uniformity of $C>0$ over the class of fields $X_\epsilon$ stated in the lemma is a simple consequence of the fact that the expectation that appears in the integrand in (58) can be bounded uniformly in this class via Gaussian comparison (see Corollary 2).

Now, let us split the *v*-integral in (58) into two parts, namely when $|u-v| \le 2\epsilon$ or $2\epsilon \le |u-v| \le r$. We proceed by considering two separate cases.

- Case 1: $p \ge 2$. For the first part where $|u-v| \le 2\epsilon$, we have
  $$\begin{aligned}&\int _{|u-v| \le 2\epsilon } \frac{dv}{(|u-v| \vee \epsilon )^{\gamma ^2}}\mathbb {E}\left[ \left( \int _D \frac{M_{\gamma , \epsilon }(dx)}{(|x-u|\vee \epsilon )^{\gamma ^2}(|x-v|\vee \epsilon )^{\gamma ^2}} \right) ^{p-2}\right] \\&\qquad \le C \epsilon ^{d-\gamma ^2}\mathbb {E}\left[ \left( \int _D \frac{M_{\gamma , \epsilon }(dx)}{(|x-u|\vee \epsilon )^{2\gamma ^2}} \right) ^{p-2}\right] \\&\qquad \le C \epsilon ^{d-\gamma ^2} \Bigg \{\mathbb {E}\left[ \left( \epsilon ^{-2\gamma ^2} M_{\gamma , \epsilon }(B(u, \epsilon )) \right) ^{p-2}\right] \\&\qquad \qquad \qquad \qquad + \mathbb {E}\left[ \left( \int _{\{|x-u| \ge \epsilon \} \cap D} \frac{M_{\gamma , \epsilon }(dx)}{(|x-u|\vee \epsilon )^{2\gamma ^2}} \right) ^{p-2}\right] \Bigg \}\\&\qquad \le C \epsilon ^{d-\gamma ^2} \epsilon ^{-\frac{\gamma ^2}{2}(p-2)} \le C \end{aligned}$$
  where the second last inequality follows from Lemma 8. As for the part where $|u-v| \in [2\epsilon , r]$, we first focus on the expected value in (58) and split it into two terms, depending on whether *x* is closer to *u* or *v*. By symmetry, it suffices to deal with
  $$\begin{aligned}&\mathbb {E}\left[ \left( \int _{\{ |x-u| \le |x-v| \} \cap D} \frac{M_{\gamma , \epsilon }(dx)}{(|x-u|\vee \epsilon )^{\gamma ^2}(|x-v|\vee \epsilon )^{\gamma ^2}} \right) ^{p-2}\right] \\&\qquad \le C \Bigg \{ \mathbb {E}\left[ \left( \int _{\{ |x-u| \ge 2|u-v| \}} \frac{M_{\gamma , \epsilon }(dx)}{|x-u|^{2\gamma ^2}} \right) ^{p-2}\right] \\&\qquad \qquad \qquad \qquad + \mathbb {E}\left[ \left( \int _{\{ |x-u| \le 2|u-v| \}} \frac{M_{\gamma , \epsilon }(dx)}{(|x-u|\vee \epsilon )^{\gamma ^2}|u-v|^{\gamma ^2}} \right) ^{p-2}\right] \Bigg \} \end{aligned}$$
  where
  $$\begin{aligned} \mathbb {E}\left[ \left( \int _{\{ |x-u| \ge 2|u-v| \}} \frac{M_{\gamma , \epsilon }(dx)}{|x-u|^{2\gamma ^2}} \right) ^{p-2}\right]&\le C |u-v|^{-\frac{\gamma ^2}{2}(p-2)} \end{aligned}$$
  and
  $$\begin{aligned}&\mathbb {E}\left[ \left( \int _{\{ |x-u| \le 2|u-v| \}} \frac{M_{\gamma , \epsilon }(dx)}{(|x-u|\vee \epsilon )^{\gamma ^2} |u-v|^{\gamma ^2}} \right) ^{p-2}\right] \\&\qquad \le C |u-v|^{-\gamma ^2(p-2)} \mathbb {E}\left[ \left( \int _{\{|x-u| \le 2|u-v| \}} \frac{M_{\gamma , \epsilon }(dx)}{|x-u|^{\gamma ^2}} \right) ^{p-2}\right] \\&\qquad \le C |u-v|^{-\gamma ^2(p-2)} |u-v|^{\frac{\gamma ^2}{2}(p-2)} \le C |u-v|^{-\frac{\gamma ^2}{2}(p-2)} \end{aligned}$$
  again by Lemma 8. Therefore,
  $$\begin{aligned}&\int _{2\epsilon \le |u-v| \le r} \frac{dv}{(|u-v| \vee \epsilon )^{\gamma ^2}}\mathbb {E}\left[ \left( \int _D \frac{M_{\gamma , \epsilon }(dx)}{(|x-u|\vee \epsilon )^{\gamma ^2}(|x-v|\vee \epsilon )^{\gamma ^2}} \right) ^{p-2}\right] \\&\qquad \le C\int _{2\epsilon \le |u-v| \le r} \frac{dv}{|u-v|^{\gamma ^2 + \frac{\gamma ^2}{2}(p-2)}}\\&\qquad = C \int _{2\epsilon \le |u-v| \le r} \frac{dv}{|u-v|^d} \le C \log \frac{r}{\epsilon } \end{aligned}$$
  which implies the desired inequality.
- Case 2: $p < 2$. For the first part where $|u-v| \le 2\epsilon$, we compare the Gaussian field $X_\epsilon$ with a Gaussian random variable $\mathcal {N}_\epsilon$ with variance $-\log \epsilon$ on $|x-u| \le \epsilon$, and obtain
  59 $$\begin{aligned}&\int _{|u-v| \le 2\epsilon } \frac{dv}{(|u-v| \vee \epsilon )^{\gamma ^2}}\mathbb {E}\left[ \left( \int _D \frac{M_{\gamma , \epsilon }(dx)}{(|x-u|\vee \epsilon )^{\gamma ^2}(|x-v|\vee \epsilon )^{\gamma ^2}} \right) ^{p-2}\right] \nonumber \\&\qquad \le C \int _{|u-v| \le 2\epsilon } \frac{dv}{(|u-v| \vee \epsilon )^{\gamma ^2}}\mathbb {E}\left[ \left( \int _{\{|x-u| \le \epsilon \} \cap D} \frac{e^{\gamma \mathcal {N}_\epsilon - \frac{\gamma ^2}{2} \mathbb {E}[\mathcal {N}_\epsilon ^2]}dx}{\epsilon ^{\gamma ^2}(3\epsilon )^{\gamma ^2}} \right) ^{p-2}\right] \nonumber \\&\qquad \le C \epsilon ^{d-\gamma ^2} \epsilon ^{-\frac{\gamma ^2}{2}(p-2)} \left( \int _{\{|x-u| \le \epsilon \} \cap D} \epsilon ^{-d} dx \right) ^{p-2}\nonumber \\&\qquad = C \left( \int _{\{|x-u| \le \epsilon \} \cap D} \epsilon ^{-d} dx \right) ^{p-2} \end{aligned}$$
  by Corollary 2. This can be uniformly bounded in $u \in {\overline{D}}$ by
  $$\begin{aligned} C \epsilon ^{-d} \int _{{\overline{B}}(u, \epsilon )} dx \le C \int _{{\overline{B}}(0,1)} dx \le C. \end{aligned}$$
  The second part may be treated in a similar way. If $|x-u| \le \frac{1}{2}|u-v|$, it is easy to check by triangle inequality that $\frac{1}{2} |u-v| \le |x-v| \le \frac{3}{2} |u-v|.$ Let us write $c = c(u, v):= \frac{1}{2}|u-v|$ If $\mathcal {N}_{c} \sim \mathcal {N}(0, -\log c)$ is an independent variable, we have
  $$\begin{aligned} \left| \mathbb {E}\left[ X_\epsilon (x-u) X_\epsilon (y-u)\right] - \mathbb {E}\left[ X_{\epsilon /c}(c^{-1}(x-u)) + \mathcal {N}_c)( X_{\epsilon /c}(c^{-1}(y-u)) + \mathcal {N}_c)\right] \right| \end{aligned}$$
  for any $x, y \in {\overline{B}}(u, c)$ and hence
  $$\begin{aligned}&\int _{B(0,r)}du \int _{2\epsilon \le |u-v| \le r} \frac{dv}{|u-v|^{\gamma ^2}} \mathbb {E}\left[ \left( \int _{D} \frac{M_{\gamma , \epsilon }(dx)}{(|x-u|\vee \epsilon )^{\gamma ^2}(|x-v|\vee \epsilon )^{\gamma ^2}} \right) ^{p-2}\right] \\&\le \int _{B(0,r)}du \int _{2\epsilon \le |u-v| \le r} \frac{dv}{|u-v|^{\gamma ^2}}\mathbb {E}\left[ \left( \int _{\{|x-u| \le \frac{1}{2}|u-v|\} \cap D} \frac{M_{\gamma , \epsilon }(dx)}{(|x-u|\vee \epsilon )^{\gamma ^2}(|x-v|\vee \epsilon )^{\gamma ^2}} \right) ^{p-2}\right] \\&\le C\int _{B(0,r)}du \int _{2\epsilon \le |u-v| \le r} \frac{dv}{|u-v|^{\gamma ^2}}\\&\qquad \mathbb {E}\left[ \left( \int _{{\overline{B}}(0, c) \cap (D-u)} \frac{e^{\gamma \mathcal {N}_{c} - \frac{\gamma ^2}{2} \mathbb {E}[\mathcal {N}_{c}^2]} e^{\gamma X_{\epsilon /c}(x/c) - \frac{\gamma ^2}{2} \mathbb {E}[X_{\epsilon /c}(x/c)^2]}dx}{|u-v|^{2\gamma ^2}} \right) ^{p-2}\right] \\&= C\int _{B(0,r)}du \int _{2\epsilon \le |u-v| \le r} \frac{dv}{|u-v|^{d}} \mathbb {E}\left[ \left( \int _{{\overline{B}}(0, 1) \cap \left( c^{-1}(D-u)\right) } M_{\gamma , \epsilon /c}(dx)\right) ^{p-2} \right] . \end{aligned}$$
  Arguing as before (and combined with the convergence and existence of GMC moments, see Lemma 7) we can obtain a bound for the last expectation that is uniform for all $u, v \in D$ and $\epsilon > 0$. Hence the above expression is bounded by $C r^d \log \frac{r}{\epsilon }$ and the proof is complete.

$$\square $$

#### Proof of Corollary 1

Again for simplicity we just treat the case $g \equiv 1$ here. Let us construct on the same probability space a Gaussian field *X* with covariance (9), independent of $\{X_\epsilon \}_\epsilon$, and write ${\widetilde{X}}_\epsilon (x) = X *\nu _\epsilon$ for some mollifier $\nu \in C_c^\infty (\mathbb {R}^d)$. We further introduce the notations

$$\begin{aligned} X_\epsilon ^t(x) = \sqrt{t} {\widetilde{X}}_\epsilon (x) + \sqrt{1-t} X_\epsilon (x), \qquad M_{\gamma , \epsilon }^t(dx) = e^{\gamma X_\epsilon ^t(x) - \frac{\gamma ^2}{2}\mathbb {E}[X_\epsilon ^t(x)^2]}dx \end{aligned}$$

for all $t \in [0,1]$. Using the interpolation formula from Lemma 2, we have

60 $$\begin{aligned} \nonumber&\left| \mathbb {E}\left[ M_{\gamma , \epsilon }^1(D)^p\right] - \mathbb {E}\left[ M_{\gamma , \epsilon }^0(D)^p\right] \right| \\ \nonumber&\qquad \le \frac{p(p-1) }{2} \int _0^1 dt\int _D \int _D \left| \mathbb {E}[X_\epsilon (x) X_\epsilon (y)]-\mathbb {E}[ {\widetilde{X}}_\epsilon (x) {\widetilde{X}}_\epsilon (y)]\right| \\&\qquad \qquad \qquad \qquad \qquad \qquad \times \mathbb {E}\left[ M_{\gamma , \epsilon }^t(dx) M_{\gamma , \epsilon }^t(dy)M_{\gamma , \epsilon }^t(D)^{p-2}\right] . \end{aligned}$$

Now fix $\delta > 0$ and write $r = \epsilon ^{1-\delta }$. By assumption, we have for $\epsilon > 0$ sufficiently small

$$\begin{aligned} \sup _{|x-y| \ge r} \left| \mathbb {E}[X_\epsilon (x) X_\epsilon (y)] - \mathbb {E}[{\widetilde{X}}_\epsilon (x) {\widetilde{X}}_\epsilon (y)]\right| \le \delta . \end{aligned}$$

Together with the other assumption on the covariance of $X_\epsilon$, we can bound (60) by

61 $$\begin{aligned}&\frac{p(p-1)\delta }{2} \sup _{t \in [0, 1]} \mathbb {E}\left[ M_{\gamma , \epsilon }^t(D)^{p}\right] \nonumber \\&+ \frac{Cp(p-1)}{2} \int _0^1 dt\int _D \int _{\{|x-y| \le r\}\cap D} \mathbb {E}\left[ M_{\gamma , \epsilon }^t(dx) M_{\gamma , \epsilon }^t(dy) M_{\gamma , \epsilon }^t(D)^{p-2}\right] . \end{aligned}$$

Using Gaussian comparison, $\mathbb {E}\left[ M_{\gamma , \epsilon }^t(D)^p\right]$ is, up to a multiplicative factor, upper bounded by $\mathbb {E}\left[ M_{\gamma , \epsilon }^{1}(D)^p\right]$. It then follows from Theorem 1 that the first term in (61) is of order $\mathcal {O}(\delta \log \frac{1}{\epsilon })$. As for the second term, we can bound it by

$$\begin{aligned}&\frac{Cp(p-1)}{2}\int _0^1 dt \int _D \mathbb {E}\left[ M_{\gamma , \epsilon }^t(dx) M_{\gamma , \epsilon }^t(B(x, r) \cap D) M_{\gamma , \epsilon }^t(D)^{p-2}\right] \\&\qquad \le \frac{Cp(p-1)}{2} \sum _{x \in D_r}\sup _{t \in [0, 1]} \mathbb {E}\left[ M_{\gamma , \epsilon }^t(B(x, 2r) \cap D)^2 M_{\gamma , \epsilon }^t(D)^{p-2}\right] \end{aligned}$$

where $D_r$ is a collection of points $x\in D$ such that $D \subset \bigcup _{x \in D} B(x, r)$. We can of course choose $D_r$ such that its cardinality is at most $\mathcal {O}(r^{-d})$. Combining this with Lemma 14 (which holds uniformly in $t \in [0, 1]$) we see that the second term is also $\mathcal {O}(\delta \log \frac{1}{\epsilon })$, and therefore

$$\begin{aligned} \limsup _{\epsilon \rightarrow 0^+} \frac{1}{-\log \epsilon } \left| \mathbb {E}\left[ M_{\gamma , \epsilon }^1(D)^p\right] - \mathbb {E}\left[ M_{\gamma , \epsilon }^0(D)^p\right] \right| = \mathcal {O}(\delta ). \end{aligned}$$

Since $\delta > 0$ is arbitrary, we let $\delta \rightarrow 0^+$ and conclude that $\mathbb {E}\left[ M_{\gamma , \epsilon }^1(D)^{p}\right]$ has the same leading order asymptotics as $\mathbb {E}\left[ M_{\gamma , \epsilon }^0(D)^{p}\right]$, which is described by Theorem 1. $\square$

### On circular unitary ensemble: proof of Theorem 2

The $k=2$ case was first proved in [14, Theorem 1.15], relying on the full Painlevé V description of the strong asymptotics of Toeplitz determinant with 2 merging singularities, but it is not clear how this may be extended to deal with $k > 2$ singularities with arbitrary merging behaviour. We shall show, however, that the asymptotics for critical-subcritical moments can be derived using only the following ingredients.

#### Lemma 15

The following estimates holds for any integers $k \ge 1$.

(i) (cf. [17, Theorem 1.1]) For any $s \ge 0$ and any $\delta > 0$,
  $$\begin{aligned} \mathbb {E}_{\mathrm {U(N)}}\left[ \prod _{j=1}^k |P_N(\theta _j)|^{2s}\right] = (1+o(1)) N^{ks^2}\left[ \frac{G(1+s)^2}{G(1+2s)}\right] ^k \prod _{1 \le u < v \le k}|e^{i\theta _u} - e^{i\theta _v}|^{-2s^2} \end{aligned}$$
  uniformly in $\theta _1, \dots , \theta _k \in [0, 2\pi )$ satisfying $|e^{i\theta _u} -e^{i \theta _v}| \ge N^{-(1-\delta )}$ for any distinct $u, v \in 1, \dots , k$.
(ii) (cf. [23, Theorem 1.1]) For any $s \ge 0$, there exists some constant $C>0$ such that
  $$\begin{aligned} \mathbb {E}_{\mathrm {U(N)}}\left[ \prod _{j=1}^k |P_N(\theta _j)|^{2s}\right] \le C N^{ks^2} \prod _{1 \le u < v \le k}\left( |e^{i\theta _u} - e^{i\theta _v}|\vee \frac{1}{N}\right) ^{-2s^2} \end{aligned}$$
  uniformly in $\theta _1, \dots , \theta _k \in [0, 2\pi )$ and for all *N* sufficiently large.

#### Remark 6

The asymptotics in Lemma 15 (i) was originally stated for fixed singularities $\theta _1, \dots , \theta _k \in [0, 2\pi )$ in [17], but can be shown to hold in the current setting. We explain this briefly in Appendix D.

#### Proof of Theorem 2

Let $X(e^{i\theta })$ be the log-correlated Gaussian field in (16) and $\{X_\epsilon (e^{i\theta })\}_{\epsilon > 0}$ a sequence of approximate fields in the sense of Corollary 1. If we set $\epsilon = \epsilon (N) = N^{-1}$, $s = 1/\sqrt{k}$ and $\gamma = \sqrt{2}s$, then Lemma 15 may be re-interpreted as follows:

- For any $$\delta \in (0, 1)$$,
  $$\begin{aligned} \frac{\mathbb {E}_{\mathrm {U}(N)} \left[ \prod _{j=1}^k|P_N(\theta _j)|^{2s}\right] }{\prod _{j=1}^k\mathbb {E}_{\mathrm {U}(N)} \left[ |P_N(\theta _j)|^{2s}\right] } \overset{N \rightarrow \infty }{=} (1+o(1)) \frac{\mathbb {E}\left[ \prod _{j=1}^k e^{\gamma X_{\epsilon }(e^{i\theta _j})} \right] }{\prod _{j=1}^k\mathbb {E}\left[ e^{\gamma X_{\epsilon }(e^{i\theta _j})} \right] } \end{aligned}$$
  for some *o*(1) depending on $\delta$ but uniformly in $\theta _1, \dots , \theta _k \in [0, 2\pi )$ satisfying $|e^{i\theta _u} -e^{i \theta _v}| \ge N^{-(1-\delta )}$ for any distinct $u, v \in \{1, \dots , k\}$.
- There exists some constant $C>0$ such that
  $$\begin{aligned} \frac{\mathbb {E}_{\mathrm {U}(N)} \left[ \prod _{j=1}^k|P_N(\theta _j)|^{2s}\right] }{\prod _{j=1}^k\mathbb {E}_{\mathrm {U}(N)} \left[ |P_N(\theta _j)|^{2s}\right] } \le C \frac{\mathbb {E}\left[ \prod _{j=1}^k e^{\gamma X_{\epsilon }(e^{i\theta _j})} \right] }{\prod _{j=1}^k\mathbb {E}\left[ e^{\gamma X_{\epsilon }(e^{i\theta _j})} \right] } \end{aligned}$$
  uniformly in $$\theta _1, \dots , \theta _k \in [0,2\pi )$$.

For any $\delta _1, \dots , \delta _{k-1} > 0$, let us define

$$\begin{aligned} D_{(\delta _1, \dots , \delta _{k-1})}&= D_{(\delta _1, \dots , \delta _{k-1})}(N)\\&= \{ (\theta _1, \dots , \theta _k) \in [0, 2\pi )^k: |e^{i\theta _u} - e^{i\theta _v}| \ge \epsilon ^{1-\delta _u} \quad \text {for any } v > u, u \in [k]\} \end{aligned}$$

and $D_{(\delta _1, \dots , \delta _{k-1})}^c = [0, 2\pi )^k \setminus D_{(\delta _1, \dots , \delta _{k-1})}$. Then we may expand the integer moments and write

$$\begin{aligned}&\mathrm {MoM}_{\mathrm {U}(N)}(k, s) = \frac{1}{(2\pi )^k} \int _{[0, 2\pi )^k}\mathbb {E}_{\mathrm {U}(N)} \left[ \prod _{j=1}^k|P_N(\theta _j)|^{2s}\right] d\theta _1 \dots d\theta _k\\&\qquad = \frac{\mathbb {E}_{\mathrm {U(N)}}[|P_N(0)|^{2s}]^k}{(2\pi )^k} \int _{[0, 2\pi )^k}\frac{\mathbb {E}_{\mathrm {U}(N)} \left[ \prod _{j=1}^k|P_N(\theta _j)|^{2s}\right] }{\prod _{j=1}^k\mathbb {E}_{\mathrm {U}(N)} \left[ |P_N(\theta _j)|^{2s}\right] } d\theta _1 \dots d\theta _k\\&\qquad = \frac{\mathbb {E}_{\mathrm {U(N)}}[|P_N(0)|^{2s}]^k}{(2\pi )^k}\\&\qquad \left\{ \int _{D_{(\delta _1, \dots , \delta _{k-1})}}\mathbb {E}\left[ \prod _{j=1}^k M_{\gamma , \epsilon }(d\theta _j) \right] + \mathcal {O}\left( \int _{D_{(\delta _1, \dots , \delta _{k-1})}^c}\mathbb {E}\left[ \prod _{j=1}^k M_{\gamma , \epsilon }(d\theta _j) \right] \right) \right\} \\&\qquad = \frac{\mathbb {E}_{\mathrm {U(N)}}[|P_N(0)|^{2s}]^k}{(2\pi )^k} \left\{ \mathbb {E}\left[ M_{\gamma , \epsilon }([0, 2\pi ))^k \right] + \mathcal {O}\left( \int _{D_{(\delta _1, \dots , \delta _{k-1})}^c}\mathbb {E}\left[ \prod _{j=1}^k M_{\gamma , \epsilon }(d\theta _j) \right] \right) \right\} \end{aligned}$$

where $M_{\gamma , \epsilon }(d\theta ) = e^{\gamma X_\epsilon (e^{i\theta }) - \frac{\gamma ^2}{2} \mathbb {E}[X_\epsilon (e^{i\theta })^2]}d\theta$. All we have to show now is that for suitably chosen $\delta _1, \dots , \delta _{k-1}$, the estimate

62 $$\begin{aligned} \mathbb {E}\left[ \int _{D_{(\delta _1, \dots , \delta _{k-1})}^c}\prod _{j=1}^k M_{\gamma , \epsilon }(d\theta _j)\right] = \mathcal {O}\left( c(\delta _1, \dots , \delta _{k-1}; \epsilon ) \log \frac{1}{\epsilon }\right) . \end{aligned}$$

holds for some $c(\delta _1, \dots , \delta _{k-1}; \epsilon ) > 0$ such that $c(\delta _1, \dots , \delta _{k-1}; \epsilon )$ tends to zero when we first let $\epsilon \rightarrow 0$ and then $\delta _1, \dots , \delta _{k-1} \rightarrow 0$.

We proceed in the following way. Let us fix $\delta _1 > 0$, and consider^15^

63 $$\begin{aligned}&\mathbb {E}\left[ \left( \int _{|e^{iu} - e^{i\theta _1}| \le \epsilon ^{1-\delta _1}} \frac{M_{\gamma , \epsilon }(du)}{(|e^{iu} - e^{i\theta _1}|\vee \epsilon )^{\gamma ^2}}\right) ^{k-1} \right] . \end{aligned}$$

Using the fact that $|e^{iu} - e^{i\theta _1}| = \Omega (|u-\theta _1|)$ where $|u-\theta _1|$ should be interpreted as the distance on $[0, 2\pi )$ with the end-points identified, we have the following multifractal estimates for (regularised) GMC measures (see Lemma 8):

$$\begin{aligned} \mathbb {E}\left[ \left( \int _{|e^{iu} - e^{i\theta _1}| \le \epsilon } \frac{M_{\gamma , \epsilon }(du)}{(|e^{iu} - e^{i\theta _1}|\vee \epsilon )^{\gamma ^2}}\right) ^{k-1} \right]&\le C,\\ \mathbb {E}\left[ \left( \int _{\epsilon \le |e^{iu} - e^{i\theta _1}| \le \epsilon ^{1-\delta _1}} \frac{M_{\gamma , \epsilon }(du)}{(|e^{iu} - e^{i\theta _1}|\vee \epsilon )^{\gamma ^2}}\right) ^{k-1} \right]&\le C \delta _1 \log \frac{1}{\epsilon }. \end{aligned}$$

(In this proof we use $C > 0$ to denote some constant which varies from line to line below but is uniform in every relevant parameter.) In particular, (63) is of order $\mathcal {O}(\delta _1 \log \frac{1}{\epsilon })$, and

$$\begin{aligned}&\mathbb {E}\left[ M_{\gamma , \epsilon }([0, 2\pi ))^k \right] = \int _{[0, 2\pi )} d\theta _1 \mathbb {E}\left[ e^{\gamma X_\epsilon (e^{i\theta _1}) - \frac{\gamma ^2}{2}\mathbb {E}[X_\epsilon (e^{i\theta })^2]} \left( \int _{[0, 2\pi )} M_{\gamma , \epsilon }(du)\right) ^{k-1} \right] \\&\qquad = \int _{[0, 2\pi )} d\theta _1 \mathbb {E}\left[ \left( \int _{[0, 2\pi )} e^{\gamma ^2 \mathbb {E}[X_\epsilon (e^{i\theta _1}) X_\epsilon (e^{iu})]} M_{\gamma , \epsilon }(du)\right) ^{k-1} \right] \\&\qquad = \mathcal {O}\left( \delta _1^{\frac{1}{k-1}} \log \frac{1}{\epsilon }\right) + (1+o(1)) \int _{[0, 2\pi )} d\theta _1 \mathbb {E}\left[ \left( \int _{|e^{iu}-e^{i\theta _1}| \ge \epsilon ^{1-\delta _1}} \frac{M_{\gamma , \epsilon }(du)}{|e^{i u} - e^{i\theta _1}|^{\gamma ^2}}\right) ^{k-1} \right] \end{aligned}$$

where the second equality follows from Cameron–Martin (Lemma 1), and in the last line the error term $\mathcal {O}\left( \delta _1^{\frac{1}{k-1}} \log \frac{1}{\epsilon }\right)$ comes from the removal of contribution from $|e^{iu} - e^{i\theta _1}| \le \epsilon ^{1-\delta }$, combined with the fact that the leading order is $\Omega (\log \frac{1}{\epsilon })$ (Corollary 1) and the basic estimate in Lemma 9. If $k=2$ then the previous identity reads as

$$\begin{aligned}&\mathbb {E}\left[ M_{\gamma , \epsilon }([0, 2\pi ))^2 \right] = \mathcal {O}\left( \delta _1 \log \frac{1}{\epsilon }\right) + (1+o(1)) \int _{[0, 2\pi )} d\theta _1 \int _{|e^{iu}-e^{i\theta _1}| \ge \epsilon ^{1-\delta _1}} \frac{du}{|e^{i u} - e^{i\theta _1}|^{\gamma ^2}} \end{aligned}$$

and we have verified (62) successfully.

If $k \ge 3$ we continue to ‘bring down the powers’:

$$\begin{aligned}&\int d\theta _1 \mathbb {E}\left[ \left( \int _{|e^{iu}-e^{i\theta _1}| \ge \epsilon ^{1-\delta _1}} \frac{M_{\gamma , \epsilon }(du)}{|e^{i u} - e^{i\theta _1}|^{\gamma ^2}}\right) ^{k-1} \right] \\&= \int d\theta _1 \int _{|e^{i\theta _2} - e^{i\theta _1}| \ge \epsilon ^{1-\delta _1}} \frac{d\theta _2}{|e^{i\theta _2}-e^{i\theta _1}|^{\gamma ^2}} \mathbb {E}\left[ \left( \int _{|e^{iu}-e^{i\theta _1}| \ge \epsilon ^{1-\delta _1}} \frac{e^{\gamma ^2 \mathbb {E}[X_\epsilon (e^{i\theta _2}) X_\epsilon (e^{iu})]}M_{\gamma , \epsilon }(du)}{|e^{i u} - e^{i\theta _1}|^{\gamma ^2}}\right) ^{k-2} \right] . \end{aligned}$$

Let us now pick $\delta _2 > 0$ such that $2\delta _2 < \delta _1$, and consider

$$\begin{aligned}&\mathbb {E}\left[ \left( \int _{|e^{iu}-e^{i\theta _1}| \ge \epsilon ^{1-\delta _1}, |e^{iu} - e^{i\theta _2}| \le \epsilon ^{1-\delta _2}} \frac{e^{\gamma ^2 \mathbb {E}[X_\epsilon (e^{i\theta _2}) X_\epsilon (e^{iu})]}M_{\gamma , \epsilon }(du)}{|e^{i u} - e^{i\theta _1}|^{\gamma ^2}}\right) ^{k-2} \right] \\&\le C \mathbb {E}\left[ \left( \int _{|e^{iu}-e^{i\theta _1}| \ge \epsilon ^{1-\delta _1}, |e^{iu} - e^{i\theta _2}| \le \epsilon ^{1-\delta _2}} \frac{M_{\gamma , \epsilon }(du)}{|e^{i u} - e^{i\theta _1}|^{\gamma ^2} (|e^{i u} - e^{i\theta _2}| \vee \epsilon )^{\gamma ^2}}\right) ^{k-2} \right] . \end{aligned}$$

For $\epsilon > 0$ sufficiently small, we may assume without loss of generality that $\epsilon ^{1-\delta _1} \ge 10 \epsilon ^{1-2\delta _2}$ so that

$$\begin{aligned} |e^{iu} - e^{i\theta _1}|&\ge |e^{i\theta _2} - e^{i\theta _1}| - |e^{iu} - e^{i\theta _2}| \ge \frac{1}{2} |e^{i\theta _2} - e^{i\theta _1}|, \\ \text {and}\qquad |e^{i\theta _2} - e^{i\theta _1}|&\ge \frac{1}{2} \left[ |e^{i u} - e^{i\theta _2}|+ |e^{i\theta _2} - e^{i\theta _1}|\right] \ge \frac{1}{2}|e^{iu} - e^{i\theta _1}| \end{aligned}$$

when $|e^{iu} - e^{i\theta _2}| \le \epsilon ^{1-2\delta _2} \le \epsilon ^{1-\delta _1} \le |e^{i\theta _2} - e^{i\theta _1}|$. This means that

$$\begin{aligned}&\mathbb {E}\left[ \left( \int _{|e^{iu}-e^{i\theta _1}| \ge \epsilon ^{1-\delta _1}, |e^{iu} - e^{i\theta _2}| \le \epsilon ^{1-\delta _2}} \frac{M_{\gamma , \epsilon }(du)}{|e^{i u} - e^{i\theta _1}|^{\gamma ^2} (|e^{i u} - e^{i\theta _2}| \vee \epsilon )^{\gamma ^2}}\right) ^{k-2} \right] \\&\qquad \le C |e^{i\theta _2} - e^{i\theta _1}|^{-(k-2) \gamma ^2}\mathbb {E}\left[ \left( \int _{|e^{iu} - e^{i\theta _2}| \le \epsilon ^{1-\delta _2}} \frac{M_{\gamma , \epsilon }(du)}{ (|e^{i u} - e^{i\theta _2}| \vee \epsilon )^{\gamma ^2}}\right) ^{k-2} \right] \\&\qquad \le C |e^{i\theta _2} - e^{i\theta _1}|^{-(k-2) \gamma ^2} \epsilon ^{(1-\delta _2)\frac{\gamma ^2}{2}(k-2)}\\&\qquad \le C |e^{i\theta _2} - e^{i\theta _1}|^{-(k-2) \gamma ^2} \epsilon ^{\delta _2 \frac{\gamma ^2}{2}(k-2)}\mathbb {E}\left[ \left( \int _{|e^{iu} - e^{i\theta _2}| \le \epsilon ^{1-2\delta _2}} \frac{M_{\gamma , \epsilon }(du)}{ (|e^{i u} - e^{i\theta _2}| \vee \epsilon )^{\gamma ^2}}\right) ^{k-2} \right] \\&\qquad \le C \epsilon ^{\delta _2 \frac{\gamma ^2}{2}(k-2)}\mathbb {E}\left[ \left( \int _{|e^{iu} - e^{i\theta _2}| \le \epsilon ^{1-2\delta _2}} \frac{M_{\gamma , \epsilon }(du)}{(|e^{i u} - e^{i\theta _1}|\vee \epsilon )^{\gamma ^2} (|e^{i u} - e^{i\theta _2}| \vee \epsilon )^{\gamma ^2}}\right) ^{k-2} \right] \end{aligned}$$

where the second and third inequalities follow from the multifractal estimates (Lemma 8) again. In particular,

$$\begin{aligned}&\int d\theta _1 \int _{|e^{i\theta _2} - e^{i\theta _1}| \ge \epsilon ^{1-\delta _1}} \frac{d\theta _2}{|e^{i\theta _2}-e^{i\theta _1}|^{\gamma ^2}} \mathbb {E}\left[ \left( \int _{\begin{array}{c} |e^{iu}-e^{i\theta _1}| \ge \epsilon ^{1-\delta _1} \\ |e^{iu} - e^{i\theta _2}| \le \epsilon ^{1-\delta _2} \end{array}} \frac{M_{\gamma , \epsilon }(du)}{|e^{i u} - e^{i\theta _1}|^{\gamma ^2} (|e^{i u} - e^{i\theta _2}| \vee \epsilon )^{\gamma ^2}}\right) ^{k-2} \right] \\&\qquad \le C \epsilon ^{\delta _2\frac{\gamma ^2}{2}(k-2)}\int d\theta _1 \int _{|e^{i\theta _2} - e^{i\theta _1}| \ge \epsilon ^{1-\delta _1}} \frac{d\theta _2}{|e^{i\theta _2}-e^{i\theta _1}|^{\gamma ^2}}\\&\qquad \qquad \times \mathbb {E}\left[ \left( \int _{|e^{iu} - e^{i\theta _2}| \le \epsilon ^{1-2\delta _2}} \frac{M_{\gamma , \epsilon }(du)}{(|e^{i u} - e^{i\theta _1}|\vee \epsilon )^{\gamma ^2} (|e^{i u} - e^{i\theta _2}| \vee \epsilon )^{\gamma ^2}}\right) ^{k-2} \right] \\&\qquad \le C \epsilon ^{\delta _2\frac{\gamma ^2}{2}(k-2)} \mathbb {E}\left[ M_{\gamma , \epsilon }([0, 2\pi )^k\right] \le C \epsilon ^{\delta _2\frac{\gamma ^2}{2}(k-2)} \log \frac{1}{\epsilon }, \end{aligned}$$

and using the basic estimate from Lemma 9 we have

$$\begin{aligned}&\int d\theta _1 \int _{|e^{i\theta _2} - e^{i\theta _1}| \ge \epsilon ^{1-\delta _1}} \frac{d\theta _2}{|e^{i\theta _2}-e^{i\theta _1}|^{\gamma ^2}} \mathbb {E}\left[ \left( \int _{|e^{iu}-e^{i\theta _1}| \ge \epsilon ^{1-\delta _1}} \frac{e^{\gamma ^2 \mathbb {E}[X_\epsilon (e^{i\theta _2}) X_\epsilon (e^{iu})]}M_{\gamma , \epsilon }(du)}{|e^{i u} - e^{i\theta _1}|^{\gamma ^2}}\right) ^{k-2} \right] \\&= \int d\theta _1 \int _{|e^{i\theta _2} - e^{i\theta _1}| \ge \epsilon ^{1-\delta _1}} \frac{d\theta _2}{|e^{i\theta _2}-e^{i\theta _1}|^{\gamma ^2}} \mathbb {E}\left[ \left( \int _{\begin{array}{c} |e^{iu}-e^{i\theta _1}| \ge \epsilon ^{1-\delta _1} \\ |e^{iu} - e^{i\theta _2}| \ge \epsilon ^{1-\delta _2} \end{array}} \frac{M_{\gamma , \epsilon }(du)}{|e^{i u} - e^{i\theta _1}|^{\gamma ^2} |e^{i u} - e^{i\theta _2}|^{\gamma ^2}}\right) ^{k-2} \right] \\&\qquad + \mathcal {O}\left( c(\delta _2; \epsilon ) \log \frac{1}{\epsilon }\right) \end{aligned}$$

for some suitable $c(\delta _2; \epsilon )$. This procedure can be repeated until we bring down all the powers, leading to the identity

$$\begin{aligned} \mathbb {E}\left[ M_{\gamma , \epsilon }([0, 2\pi ))^k\right]&= \int _{D_{(\delta _1, \dots , \delta _{k-1})}} \frac{d\theta _1 \dots d\theta _k}{\prod _{1 \le u < v \le k} |e^{i\theta _u} - e^{i\theta _v}|^{\gamma ^2}} + \mathcal {O}\left( c(\delta _1, \dots , \delta _{k-1}; \epsilon ) \log \frac{1}{\epsilon }\right) \\&= \mathbb {E}\left[ \int _{D_{(\delta _1, \dots , \delta _{k-1})}} \prod _{j=1}^k M_{\gamma , \epsilon }(d\theta _j)\right] + \mathcal {O}\left( c(\delta _1, \dots , \delta _{k-1}; \epsilon ) \log \frac{1}{\epsilon }\right) \end{aligned}$$

for some suitable $c(\delta _1, \dots , \delta _{k-1}; \epsilon )$ and this necessarily implies the validity of (62). Since all these $\delta _j$’s are arbitrary, we conclude that

$$\begin{aligned} \mathrm {MoM}_{\mathrm {U}(N)}(k, s)&= (1+o(1)) \frac{\mathbb {E}_{\mathrm {U(N)}}[|P_N(0)|^{2s}]^k}{(2\pi )^k} \mathbb {E}\left[ M_{\gamma , \epsilon }([0, 2\pi ))^k \right] \end{aligned}$$

as $\epsilon \rightarrow 0^+$ and our proof is complete. $\square$

## Data Availability

Data sharing not applicable to this article as no datasets were generated or analysed during the current study.

## A Circular Unitary Ensemble and Subcritical GMCs

Earlier in the introduction, we mentioned that the asymptotics (6) for $\mathrm {MoM}_{\mathrm {U}(N)}(k, s)$ is essentially resolved in the subcritical regime where $k < 1/s^2$ and $0< s < 1$. By essentially resolved we meant that the GMC argument could be made rigorous provided that one could establish the convergence of moments

64 $$\begin{aligned} \lim _{N \rightarrow \infty } \mathbb {E}_{\mathrm {U}(N)} \left[ \left( \int _0^{2\pi } \frac{|P_N(\theta )|^{2s}}{\mathbb {E}_{\mathrm {U}(N)}|P_N(\theta )|^{2s}} d\theta \right) ^{k}\right] = \mathbb {E}\left[ \left( \int _0^{2\pi } M_{\gamma }(d\theta )\right) ^k\right] , \end{aligned}$$

where $\gamma = \sqrt{2}s$ and $M_{\gamma }(d\theta ) =e^{\gamma X(e^{i\theta }) - \frac{\gamma ^2}{2}\mathbb {E}[X(e^{i\theta })^2]} d\theta$ is the GMC measure associated to the Gaussian free field (4). In many situations, the condition (64) can be verified. For instance, if $k \in \mathbb {N}$, we can consider the expanded moments (2) and show that the main contribution to the multiple integral comes from the set where the angles $\theta _1, \dots , \theta _k$ are at a macroscopic distance apart from each other, in which case existing asymptotics for Toeplitz determinant with singularities may be applied. This was done as part of [23, Corollary 2.1].

For non-integer values of *k*, the expanded moment approach no longer works. One may want to rely on the weak convergence

65 $$\begin{aligned} \frac{|P_N(\theta )|^{2s}}{\mathbb {E}_{\mathrm {U}(N)}|P_N(\theta )|^{2s}} d\theta&=: \mu _{N,s}(d\theta )\nonumber \\&\xrightarrow [N \rightarrow \infty ]{d} M_{\gamma }(d\theta ) =e^{\gamma X(e^{i\theta }) - \frac{\gamma ^2}{2}\mathbb {E}[X(e^{i\theta })^2]} d\theta , \qquad \theta \in [0, 2\pi ), \end{aligned}$$

but this distributional result per se does not immediately imply (64). Fortunately, the proof of (65) is based on a moment method [35, 45], and stronger conclusions may be obtained. For example, in the $L^2$-regime [45] where $s^2 < 1/2$ , there exists a further sequence of random measures $\mu _{N, s}^{(M)}(d\theta )$ such that

$$\begin{aligned} \lim _{M \rightarrow \infty } \lim _{N \rightarrow \infty }\mathbb {E}_{\mathrm {U}(N)}\left[ \left( \mu _{N, s}([0, 2\pi ))-\mu _{N, s}^{(M)}([0, 2\pi ))\right) ^2\right] = 0 \end{aligned}$$

and

$$\begin{aligned}&\lim _{M \rightarrow \infty } \lim _{N \rightarrow \infty }\mathbb {E}_{\mathrm {U}(N)}\left[ \left( \mu _{N, s}^{(M)}([0, 2\pi ))\right) ^k\right] \\&\quad = \mathbb {E}\left[ \left( \int _0^{2\pi } M_{\gamma }(d\theta )\right) ^k\right] \qquad \text {for any }k \in (0, 1/s^2). \end{aligned}$$

Using these ingredients, we may conclude that for any $1 \le k \le 2$,

$$\begin{aligned}&\mathbb {E}_{\mathrm {U}(N)} \left[ \left( \int _0^{2\pi } \frac{|P_N(\theta )|^{2s}}{\mathbb {E}_{\mathrm {U}(N)}|P_N(\theta )|^{2s}} d\theta \right) ^{k}\right] ^{1/k}\\&=\mathbb {E}_{\mathrm {U}(N)}\left[ \left( \mu _{N, s}^{(M)}([0, 2\pi ))\right) ^k\right] + \mathcal {O}\left( \mathbb {E}_{\mathrm {U}(N)}\left[ \left( \mu _{N, s}([0, 2\pi ))-\mu _{N, s}^{(M)}([0, 2\pi ))\right) ^2\right] ^{1/2}\right) \\&\xrightarrow {N \rightarrow \infty , M \rightarrow \infty } \mathbb {E}\left[ \left( \int _0^{2\pi } M_{\gamma }(d\theta )\right) ^k\right] \end{aligned}$$

and the case where $k \in (0, 1)$ is similar. This approach is generalised in [35] to the $L^1$-regime where $s^2 \in [1/2, 1)$, from which one could obtain (64) at least for all $k \le 1$. Of course, these results have not covered the entire subcritical regime $k < 1/s^2$ in the asymptotic analysis of moments of moments (1) of CUE matrices, but we expect that more refined analysis along a similar line of argument may make this possible and that the problem is conceptually solved.

## B GMC Moments: Renormalisation from Below

Here we briefly mention the ‘lower continuity’ of GMC moments up to renormalisation at critical exponent in the following sense.

### Proposition 1

Let $d \le 2$, $\gamma \in (0, \sqrt{2d})$, $p_c = 2d / \gamma ^2$ and $M_{\gamma }(dx)$ be the GMC measure associated to the log-correlated field (9). If $g \ge 0$ is a continuous function on ${\overline{D}}$, then

$$\begin{aligned} \lim _{\alpha \rightarrow p_c^-} (p_c-1-\alpha ) \mathbb {E}\left[ \left( \int _D g(x) M_{\gamma }(dx)\right) ^{\alpha }\right] = \left( \int _D e^{d(p_c-1)f(u, u)} g(u)^{p_c} du\right) (p_c-1) {\overline{C}}_{\gamma , d} \end{aligned}$$

and

$$\begin{aligned} \lim _{{\widetilde{\gamma }} \rightarrow \gamma ^-} \left( \frac{2d}{{\widetilde{\gamma }}^2} - p_c\right) \mathbb {E}\left[ \left( \int _D g(x) M_{{\widetilde{\gamma }}}(dx)\right) ^{p_c}\right] = \left( \int _D e^{d(p_c-1)f(u, u)} g(u)^{p_c} du\right) (p-1) {\overline{C}}_{\gamma , d}. \end{aligned}$$

### Proof

We only sketch the proof of the first limit for $g \equiv 1$ and $f \ge 0$. Let us fix $r \in (0, 1/4)$ as before. By considerations similar to those in Section 3.2.1, we have

66 $$\begin{aligned} \mathbb {E}\left[ \left( \int _D M_{\gamma }(dx)\right) ^{\alpha }\right]&= \int _D du \mathbb {E}\left[ \left( \int _D \frac{e^{\gamma ^2 f(x, u)}}{|x-u|^{\gamma ^2}} M_{\gamma }(dx)\right) ^{\alpha -1}\right] \nonumber \\&= \mathcal {O}(1) + \int _D du \mathbb {E}\left[ \left( \int _{D\cap \{|x-u| \le r\}} \frac{e^{\gamma ^2 f(x, u)}}{|x-u|^{\gamma ^2}} M_{\gamma }(dx)\right) ^{\alpha -1}\right] . \end{aligned}$$

Given the uniform continuity of *f* on ${\overline{D}} \times {\overline{D}}$, it has a modulus of continuity $\omega (\cdot )$ so that

$$\begin{aligned} |f(x, u) - f(u,u)| \le \omega (r) \qquad \forall x \in B(u, r) \quad \forall u \in D \end{aligned}$$

and $\omega (r) \rightarrow 0$ as $r \rightarrow 0^+$. Based on the same argument of Gaussian comparison we saw in Lemma 12, (66) can be rewritten as

$$\begin{aligned}&\mathcal {O}(1) + \left( 1+\mathcal {O}(\omega (r))\right) \left( \int _D du \mathbb {E}\left[ \left( e^{\gamma ^2 f(u, u)} e^{\gamma \mathcal {N}_{u} - \frac{\gamma ^2}{2} \mathbb {E}[\mathcal {N}_{u}^2]}\right) ^{\alpha -1}\right] \right) \\&\qquad \mathbb {E}\left[ \left( \int _{|x|\le r} e^{\gamma {\overline{X}}(x) - \frac{\gamma ^2}{2} \mathbb {E}[{\overline{X}}(x)^2]}\frac{dx}{|x|^{\gamma ^2}} \right) ^{\alpha } \right] \\&= \mathcal {O}(1) + \left( 1+\mathcal {O}(\omega (r))\right) \left( \int _D e^{\frac{\gamma ^2}{2}\alpha (\alpha -1) f(u, u)} du\right) \mathbb {E}\left[ \left( \int _{|x|\le r} e^{\gamma {\overline{X}}(x) - \frac{\gamma ^2}{2} \mathbb {E}[{\overline{X}}(x)^2]}\frac{dx}{|x|^{\gamma ^2}} \right) ^{\alpha } \right] . \end{aligned}$$

Our claim follows immediately by first applying Lemma 10 and then sending *r* to 0. $\square$

## C Supercritical Moments of Approximate GMCs

Let us again consider $X_\epsilon := X *\nu _\epsilon$ for some mollifier $\nu \in C_c^\infty (\mathbb {R}^d)$ and study the integrand on the RHS of

$$\begin{aligned} \mathbb {E}\left[ M_{\gamma , \epsilon }(D)^{p}\right] = \int _D du\mathbb {E}\left[ \left( \int _D \frac{e^{\gamma ^2 f_\epsilon (x, u)}}{\left( |x-u|\vee \epsilon \right) ^{\gamma ^2}} e^{\gamma X_{\epsilon }(x) - \frac{\gamma ^2}{2} \mathbb {E}[X_\epsilon (x)^2]} dx\right) ^{p-1}\right] . \end{aligned}$$

Suppose we are in the ‘supercritical’ case where $p > 2d/\gamma ^2$ for any $\gamma \in \mathbb {R}$. Looking at (part of) the microscopic contribution, one can show that

$$\begin{aligned} \mathbb {E}\left[ \left( \int _{|x-u| \le \epsilon } \frac{e^{\gamma ^2 f_\epsilon (x, u)}}{\left( |x-u|\vee \epsilon \right) ^{\gamma ^2}} e^{\gamma X_{\epsilon }(x) - \frac{\gamma ^2}{2} \mathbb {E}[X_\epsilon (x)^2]} dx\right) ^{p-1}\right] = \Omega \left( \epsilon ^{\frac{\gamma ^2}{2}(p-1)\left( \frac{2d}{\gamma ^2} - p\right) }\right) \end{aligned}$$

captures the size of the leading order of $\mathbb {E}[M_{\gamma , \epsilon }(D)^p]$. On the other hand, for any $\delta > 0$, it is possible to show that the contribution from $\{|x-u| \ge \epsilon ^{1-\delta }\}$ does not yield the leading order, so that

$$\begin{aligned}&\mathbb {E}\left[ \left( \int _D \frac{e^{\gamma ^2 f_\epsilon (x, u)}}{\left( |x-u|\vee \epsilon \right) ^{\gamma ^2}} e^{\gamma X_{\epsilon }(x) - \frac{\gamma ^2}{2} \mathbb {E}[X_\epsilon (x)^2]} dx\right) ^{p-1}\right] \\&\qquad \overset{\epsilon \rightarrow 0^+}{\sim } \mathbb {E}\left[ \left( \int _{D\cap \{|x-u| \le \epsilon ^{1-\delta }\}} \frac{e^{\gamma ^2 f_\epsilon (x, u)}}{\left( |x-u|\vee \epsilon \right) ^{\gamma ^2}} e^{\gamma X_{\epsilon }(x) - \frac{\gamma ^2}{2} \mathbb {E}[X_\epsilon (x)^2]} dx\right) ^{p-1}\right] \end{aligned}$$

by Lemma 9. Since $\delta > 0$ is arbitrary, a finer analysis will arrive at the conclusion that the leading coefficient depends only on the microscopic regime where $|x-u| = \mathcal {O}(\epsilon )$.

To proceed further, let us define $X_\epsilon (x; u) = X_\epsilon (x) - X_\epsilon (u)$ and rewrite the last expression as

67 $$\begin{aligned}&\mathbb {E}\left[ \left( \int _{D\cap \{ |x-u| \le \epsilon ^{1-\delta }\}} e^{\gamma ^2 \mathbb {E}[X_\epsilon (x) X_\epsilon (u)]}e^{\gamma X_{\epsilon }(x) - \frac{\gamma ^2}{2} \mathbb {E}[X_\epsilon (x)^2]} dx\right) ^{p-1}\right] \nonumber \\&= e^{\frac{\gamma ^2}{2}p(p-1) \mathbb {E}[X_\epsilon (u)^2]} \nonumber \\&\qquad \times \mathbb {E}\Bigg [e^{\gamma (p-1) X_\epsilon (u) - \frac{\gamma ^2(p-1)^2}{2} \mathbb {E}[X_\epsilon (u)^2]}\left( \int _{D\cap \{ |x-u| \le \epsilon ^{1-\delta }\}} e^{\gamma X_{\epsilon }(x;u) - \frac{\gamma ^2}{2} \mathbb {E}[X_\epsilon (x;u)^2]} dx\right) ^{p-1}\Bigg ]\nonumber \\&= e^{\frac{\gamma ^2}{2}p(p-1) \mathbb {E}[X_\epsilon (u)^2]} \mathbb {E}\Bigg [\left( \int _{D\cap \{|x-u| \le \epsilon ^{1-\delta }\}} e^{\gamma ^2(p-1) \mathbb {E}[X_\epsilon (u) X_\epsilon (x; u)]}e^{\gamma X_{\epsilon }(x;u) - \frac{\gamma ^2}{2} \mathbb {E}[X_\epsilon (x;u)^2]} dx\right) ^{p-1}\Bigg ] \end{aligned}$$

by Cameron–M-artin (Lemma 1). We have

$$\begin{aligned} \mathbb {E}[X_\epsilon (u) X_\epsilon (x; u)]&= - \log \left( \frac{|u-x|}{\epsilon } \vee 1\right) + \left( f_\epsilon (u, x) - f_\epsilon (u, u)\right) , \\ \mathbb {E}[X_\epsilon (u+\epsilon s; u) X_\epsilon (u + \epsilon t; u)]&= \log \left( |s| \vee 1\right) + \log \left( |t| \vee 1\right) - \log \left( |s-t| \vee 1\right) \\&\qquad + f_\epsilon (u, u) + f_\epsilon (u+\epsilon s, u+ \epsilon t) - f_\epsilon (u, u+ \epsilon s) - f_\epsilon (u, u+ \epsilon t). \end{aligned}$$

In particular, the Gaussian field $\mathbf {X}_\epsilon (s; u) := X_\epsilon (u + \epsilon s; u)$ does not have log-singularity anymore as $\epsilon \rightarrow 0^+$, and with a simple change of variable we see that (67) is asymptotically equal to

$$\begin{aligned}&e^{\frac{\gamma ^2}{2}p(p-1) \mathbb {E}[X_\epsilon (u)^2]} \epsilon ^{d(p-1)}\\&\quad \times \mathbb {E}\Bigg [\left( \int _{\mathbb {R}^d}\frac{e^{\gamma ^2(p-1) \left[ f_\epsilon (u, u + \epsilon s) - f_\epsilon (u, u)\right] }}{\left( |s| \vee 1\right) ^{\gamma ^2 (p-1)}} \frac{e^{-\frac{\gamma ^2}{2}\left[ f_\epsilon (u,u) + f_\epsilon (u+\epsilon s, u+\epsilon s) - 2f_\epsilon (u, u+\epsilon s)\right] }}{\left( |s| \vee 1\right) ^{\gamma ^2}} e^{\gamma \mathbf {X}_{\epsilon }(s;u)} ds\right) ^{p-1}\Bigg ]\\&= \epsilon ^{\frac{\gamma ^2}{2}(p-1)\left( \frac{2d}{\gamma ^2} - p\right) } e^{-\frac{\gamma ^2}{2}(p-1)^2f_\epsilon (u, u)} \mathbb {E}\Bigg [\left( \int _{\mathbb {R}^d}\frac{e^{p\gamma ^2f_\epsilon (u, u + \epsilon s) - \frac{\gamma ^2}{2} f_\epsilon (u+\epsilon s, u+\epsilon s)}}{\left( |s| \vee 1\right) ^{p\gamma ^2}} e^{\gamma \mathbf {X}_{\epsilon }(s;u)}ds\right) ^{p-1}\Bigg ]. \end{aligned}$$

The leading order coefficient remains $\Omega (1)$ as $\epsilon \rightarrow 0^+$, but it is very sensitive to the choice of approximate fields $X_\epsilon$ (or equivalently the mollifiers $\nu$). In addition, the Gaussian quantity here is not able to accurately capture the properties of e.g. random matrix models, the microscopic statistics of which are described by the Sine process instead.

Let us also quickly comment on the ‘critical-supercritical’ case, i.e. $p = 2d/\gamma ^2 \in (0, 1)$. If we look at the toy model of exponential functional of Brownian motion, i.e.

$$\begin{aligned} \mathbb {E}\left[ \left( \int _0^T e^{\gamma (B_t - \mu t)}dt\right) ^{p-1}\right] \end{aligned}$$

we now have a Brownian motion with drift $-\mu = -\frac{\gamma }{2}(p-1) > 0$, so that the leading order contribution to the above integral should come from near $T = -(1+o(1))\log \epsilon$. In terms of the GMC asymptotics, it again suggests that the leading order depends heavily on the microscopic region.

## D Slowly Merging Fisher–Hartwig Singularities

We now discuss why the strong asymptotics in Lemma 15 (i) continue to hold when we allow singularities $\theta _1, \dots , \theta _k$ to possibly merge at a a rate not faster than $N^{-(1-\delta )}$. Since this requires an inspection of the Riemann-Hilbert analysis that is largely independent of the main text of the current article, we shall follow the notations in [16, 17] as closely as possible in this appendix unless otherwise specified. Much of the following content is rather standard in this kind of study, and we shall only briefly explain the arguments with heavy reference to existing literature where interested readers can find more details.

To begin with, it is helpful to have a quick recap of the proof of the strong asymptotics of Toeplitz determinant with Fisher–Hartwig singularities.^16^ Letting^17^

$$\begin{aligned} f(z) = \prod _{j=1}^m |z-z_j|^{2\alpha _j} \quad \text {where } z_j = e^{i\theta _j}, \quad j = 1, \dots , m, \quad 0 \le \theta _1< \dots< \theta _m < 2\pi \end{aligned}$$

where $\Re (\alpha _j) > -\frac{1}{2}$ for $j = 1, \dots , m$, and

$$\begin{aligned} f_j = \frac{1}{2\pi }\int _0^{2\pi } f(e^{i\theta }) e^{-ij\theta }d\theta , \qquad j \in \mathbb {Z}, \end{aligned}$$

we are interested in the asymptotics for *n*-dimensional Toeplitz determinant with symbol *f*, i.e. $D_n(f) = D_n(\alpha _1, \dots , \alpha _m) := \det \left( f_{j-k}\right) _{j,k=0}^{n-1}$, as $n \rightarrow \infty$. This is closely related to Haar-distributed unitary matrices since the Heine-Szegö identity (see e.g. [17, Equation (2.10)]) implies that

$$\begin{aligned} \mathbb {E}_{\mathrm {U(N)}}\left[ \prod _{j=1}^k |\det (I - U_Ne^{-i\theta _j})|^{2s}\right] = D_{N-1}(\underbrace{s, \dots , s}_{\text {{ k} terms}}). \end{aligned}$$

Furthermore, the Toeplitz determinants are connected to orthogonal polynomials on the unit circle in the following way: there exists a dual pair of orthogonal polynomials^18^

$$\begin{aligned} \phi _k(z) = \chi _k z^k + \dots \qquad \text {and} \qquad {\widehat{\phi }}_k(z) = \chi _k z^k + \dots \end{aligned}$$

of degree *k* with positive leading coefficient $\chi _k$ for each $k \in \mathbb {N}$ such that (writing $z=e^{i\theta }$)

$$\begin{aligned}&\frac{1}{2\pi }\int _0^{2\pi } \phi _k(z) z^{-j} f(z) d\theta = \chi _k^{-1} \delta _{jk},\\&\qquad \frac{1}{2\pi }\int _0^{2\pi } {\widehat{\phi }}_k(z) z^{j} f(z) d\theta = \chi _k^{-1} \delta _{jk}, \qquad j = 0, 1, \dots , k \end{aligned}$$

and it can be shown that (see e.g. [17, Section 2])

$$\begin{aligned} \chi _k = \sqrt{\frac{D_k(f)}{D_{k+1}(f)}} \qquad \text {so that} \qquad D_n(f) := \prod _{j=1}^{n-1} \chi _j^{-2}. \end{aligned}$$

The Riemann-Hilbert analysis kicks in when we try to encode these objects in some matrix-valued functions: for each $k \in \mathbb {N}$, one can define

$$\begin{aligned} Y(z) = Y^{(k)}(z) = \begin{pmatrix} \chi _k^{-1} \phi _k(z) &{} \chi _k^{-1} \int _C \frac{\phi _k(\xi )}{\xi - z} \frac{f(\xi )d\xi }{2\pi \xi ^k}\\ -\chi _{k-1} z^{k-1}{\widehat{\phi }}_{k-1}(z^{-1}) &{} -\chi _{k-1} \int _C \frac{{\widehat{\phi }}_{k-1}(\xi ^{-1})}{\xi - z} \frac{f(\xi )d\xi }{2\pi \xi } \end{pmatrix} \quad C = \text {unit circle} \end{aligned}$$

and show that it is the unique solution to the so-called *Y* Riemann-Hilbert problem (RHP), see [17, Equations (2.5-2.9)]. One can then show that (see [17, Equation (5.3)] and the references therein) for $\gamma \in \cup _{j=1}^m \{\alpha _j\}$, we have

68 $$\begin{aligned} \frac{\partial }{\partial \gamma } \log D_n(f)&= -2\frac{\frac{\partial \chi _n}{\partial \gamma }}{\chi _n} \left( n + \sum _{j=1}^m \alpha _j \left\{ 1 - {\widetilde{Y}}_{12}^{(n)}(z_j) Y_{21}^{(n+1)}(z_j) - z_j Y_{11}^{(n)}(z_j) {\widetilde{Y}}_{22}^{(n+1)}(z_j)\right\} \right) \nonumber \\&\qquad - 2\sum _{j=1}^m \alpha _j \left( {\widetilde{Y}}_{12}^{(n)}(z_j) \frac{\partial }{\partial \gamma }Y_{21}^{(n+1)}(z_j) - z_j \frac{\partial }{\partial \gamma }Y_{11}^{(n)}(z_j) {\widetilde{Y}}_{22}^{(n+1)}(z_j)\right) \end{aligned}$$

where $Y^{(k)}_{ij}$ is the *ij*-th entry of the solution to the $Y^{(k)}$-RHP, and ${\widetilde{Y}}^{(k)}_{ij}$ are the regularised versions.^19^ In particular, the RHS of (68) only depends on the ‘last two’ RHPs $Y^{(n)}$ and $Y^{(n+1)}$. Provided that we can establish their asymptotics (including any derivatives that have appeared in the differential identity) as $n \rightarrow \infty$, we can obtain our desired formula by integrating both sides with respect to each variable $\gamma = \alpha _j$, $j = 1, \dots , m$, using the fact that $D_n(0, \dots , 0) = 1$.

So far everything we have discussed applies regardless of the behaviour of the singularities, and it all boils down to the analysis of the asymptotics for *Y*- RHP. This relies on the method of non-linear steepest descent, first applied in the random matrix context in [18], which involves the following steps:

- The *Y*-RHP is transformed to the so-called *T*-RHP which ‘normalises the behaviour at infinity’, see e.g. [17, Equation (4.1)].
- The *T*-RHP is further transformed to a new *S*-RHP, a procedure known as opening of lenses, see e.g. [17, Equation (4.2)] and the discussion that follows it.
- The *S*-RHP is approximated by two sets of functions:
  - the global parametrix *N*(*z*) in [17, Equations (4.7-4.10)] when *z* is away from the unit circle and not close to any of the singularities;
  - a collection of functions $P_{z_j}(z)$, known as the the local parametrices (see [17, Equation (4.16)] and other related definitions), each of which is defined on a small neighbourhood $U_{z_j}$ of $z_j = e^{i\theta _j}$, say $U_{z_j} := \{z \in \mathbb {C}: |z - z_j| < \epsilon \}$ for some $\epsilon > 0$ chosen such that $U_{z_j}$’s are non-overlapping.
- The *R*-RHP [17, Equation (4.26)], which measures the ‘multiplicative error’ when the *S*-RHP is approximated by the global/local parametrices, is shown to be close to the identity matrix with other ‘nice properties’ as $n \rightarrow \infty$.

When the singularities $z_1, \dots , z_m$ are fixed, the parameter $\epsilon > 0$ in the definition of $U_{z_j}$ may be chosen as some fixed number, and one can easily verify the ‘jump condition’ for the *R*-RHP in [17, Equation (4.27-4.29)] is of the form $R_+(z) = R_-(z)\Delta (z)$ with $\Delta (z) = I + \mathcal {O}(n^{-1})$. Standard analysis then shows that *R* has a unique solution and that $R-I$ has an asymptotic expansion in powers of $n^{-1}$, see more details in [17, Section 4.1].

When the singularities are allowed to vary but remain $n^{-(1-\kappa )}$ (with $\kappa > 0$) separated from each other,^20^ the first three steps of the steepest descent will still go through except that the local parametrices $P_{z_j}$ will now be defined on $U_{z_j}$ with e.g. radius $\epsilon = \epsilon _n = \frac{1}{10} n^{-(1-\kappa )}$ in order to satisfy the non-overlapping condition. The analysis of *R*-RHP with shrinking contours already appeared in [16, 17] for the ‘extension to smooth *V*(*z*)’, even though in the context of merging singularities they were perhaps first analysed in [14] and more recently in [12, 23] The consequence of shrinking contours (in the notations of [16, 17]) are as follows:

- The jump conditions [17, Equations (4.27-4.28)] remain true, but now the jump matrix
  $$\begin{aligned} \Delta (z) := I + N(z) \begin{pmatrix} 0 &{} 0 \\ f(z)^{-1} z^{\mp n} &{} 0\end{pmatrix} N(z)^{-1} \end{aligned}$$
  (where the exponent for *z* is negative if $z \in \Sigma _j^{\mathrm {out}}$ and positive if $z \in \Sigma _j^{''\mathrm {out}}$ for $j=1, \dots , m$) is no longer $I+\mathcal {O}(e^{-c n})$ for some small $c > 0$, but rather $I + \mathcal {O}(e^{-c n^c})$ where $c>0$ may possibly depend on $\kappa$ but can be made uniform in compact sets of $\alpha _1, \dots , \alpha _m$.
- The jump condition [17, Equation (4.29)] is also true, but now the matching condition for the global/local parametrices is weakened to
  $$\begin{aligned} \Delta (z) := P_{z_j}(z) N(z)^{-1} = I + \mathcal {O}(1/n\epsilon _n) = I + \mathcal {O}(n^{-\kappa }), \qquad \forall z \in \partial U_{z_j} \setminus \{ \text {intersection points} \} \end{aligned}$$
  for each $$j=1, \dots , m$$.

While $R(z) - I$ can no longer be expanded in an asymptotic series in powers of $n^{-1}$, the *R*-RHP is still a ‘small-norm problem’ and so for *n* sufficiently large it may still be solved using the Neumann series construction.^21^ In particular, one can still verify that

$$\begin{aligned} R(z) = I + o(1) \qquad \text { and } \qquad \frac{\partial }{\partial \gamma } R(z) = o(1) \end{aligned}$$

and so all the leading order asymptotics for $\chi _n$, $Y^{(n)}$ and $Y^{(n+1)}$ and its regularised versions (as well as their derivatives) appearing in (68) will remain identical to what was observed in the fixed singularity case, and hence the strong asymptotics for Toeplitz determinants with singularities may be extended to cover the slightly more general situation with a separation distance of $n^{-(1-\kappa )}$ for any $\kappa > 0$.

## Footnotes

1 Except the relatively trivial case $k = s = 1$, where the moment of moment is simply reduced to $\mathbb {E}_{\mathrm {U}(N)}\left[ |P_N(\theta )|^2\right]$.

2 Some of the results hold for complex-valued *s*, but this is beyond the scope of the discussion here.

3 See Appendix A for further discussion.

4 By which we mean $\int _D g > 0$.

5 By mollifiers we mean functions $\nu \in C_c^\infty (\mathbb {R}^d)$ such that $\nu \ge 0$ and $\int \nu = 1$.

6 By $\mathrm {Sp}(2N)$ we mean the collection of unitary symplectic matrices of size $2N \times 2N$.

7 The main reasoning is that blow-up of the ‘critical moment’ should be attributed to very positive covariance (i.e. near the diagonal), which is opposite to the ‘anti-diagonal’ behaviour, i.e. that the covariance tends to minus infinity when $\theta \rightarrow -\phi$.

8 For definiteness one may assume that dyadic points are represented by finite-length binary expansions.

9 It is expected that the leading order coefficients obtained in this model do not correspond to the analogous constants in GMC/random matrix theory, regardless of the regime under consideration.

10 The case with unit drift $\mu = 1$ was stated in [46, Theorem 2.2] but this can be extended to arbitrary $\mu > 0$ by a suitable space–time rescaling

$$\begin{aligned} (B_t^1)_{t \ge 0} \overset{d}{=} \left( \mu B_{t / \mu ^2}^\mu \right) _{t \ge 0} \qquad \text {and} \qquad (\mathcal {B}_t^1)_{t \ge 0} \overset{d}{=} \left( \mu \mathcal {B}_{t / \mu ^2}^\mu \right) _{t \ge 0}, \end{aligned}$$

which can be verified by e.g. checking the infinitesimal generators of these processes.

11 We have abused the notation in $d=2$ when we identify $\mathbb {R}^2 \cong \mathbb {C}$ and represent a point in the unit ball as a complex number.

12 This may also be obtained directly by performing a similar radial–lateral decomposition on a log-correlated field with covariance $-\log |x-y| +1_{\{|x| \ge 1\}} \log |x|+1_{\{|y| \ge 1\}} \log |y|$ on $\mathbb {R}^2$, which coincides with that of the exact field when restricted to the unit ball.

13 This can be made rigorous by integrating ${\widehat{X}}$ against test functions which we omit here.

14 In this particular lemma we do not need $\{X_\epsilon \}_\epsilon$ to be constructed via mollification of one underlying field $X(\cdot )$ as long as the covariance assumption is satisfied.

15 We suppress the fact that all our dummy variables lie in $[0, 2\pi )$ to simplify our notations in the proof.
